# Design, Synthesis, and Molecular Docking of Paracyclophanyl-Thiazole Hybrids as Novel CDK1 Inhibitors and Apoptosis Inducing Anti-Melanoma Agents

**DOI:** 10.3390/molecules25235569

**Published:** 2020-11-27

**Authors:** Ashraf A. Aly, Stefan Bräse, Alaa A. Hassan, Nasr K. Mohamed, Lamiaa E. Abd El-Haleem, Martin Nieger, Nesrin M. Morsy, Mohammed B. Alshammari, Mahmoud A. A. Ibrahim, Elshimaa M. N. Abdelhafez

**Affiliations:** 1 Department of Chemistry, Faculty of Science, Minia University, El Minia 61519, Egypt; alaahassan2001@mu.edu.eg (A.A.H.); nasrmohamed603@yahoo.com (N.K.M.); m.ibrahim@compchem.net (M.A.A.I.); 2 Institute of Organic Chemistry, Karlsruhe Institute of Technology, 76131 Karlsruhe, Germany; lamiaaelsayed2013@yahoo.com; 3 Institute of Biological and Chemical Systems–Functional Molecular Systems (IBCS-FMS), Karlsruhe Institute of Technology, Hermann-von-Helmholtz-Platz 1, D-76344 Eggenstein-Leopoldshafen, Germany; 4 Department of Chemistry, University of Helsinki, P.O. Box 55 (A. I. Virtasen aukio I), 00014 Helsinki, Finland; martin.nieger@helsinki.fi; 5 National Research Centre, Organometallic and Organometalloid Chemistry Department, Dokki, Cairo 12622, Egypt; nesrinmorsy@yahoo.com; 6 College of Sciences and Humanities, Prince Sattam bin Abdulaziz University, Alkharj 11942, Saudi Arabia; m.alshammari@psau.edu.sa; 7 Department of Medicinal Chemistry, Faculty of Pharmacy, Minia University, El Minia 61519, Egypt

**Keywords:** paracyclophane, thiazole, apoptosis, CDK1, 1,4-naphthoquinone, antitumor, melanoma, molecular docking

## Abstract

Three new series of paracyclophanyl-dihydronaphtho[2,3-*d*]thiazoles and paracyclophanyl-thiazolium bromides were designed, synthesized, and characterized by their spectroscopic data, along with X-ray analysis. One-dose assay results of anticancer activity indicated that **3a**–**e** had the highest ability to inhibit the proliferation of different cancer cell lines. Moreover, the hybrids **3c**–**e** were selected for five-dose analyses to demonstrate a broad spectrum of antitumor activity without apparent selectivity. Interestingly, **series I** compounds (*Z*)-*N*-substituted-4,9-dihydronaphtho[2,3-*d*]thiazol-3(2*H*)-yl)-4′-[2.2]paracyclophanylamide) that are carrying 1,4-dihydronaphthoquinone were more active as antiproliferative agents than their naphthalene-containing congeners (series II: substituted 2-(4′-[2.2]paracyclophanyl)hydrazinyl)-4-(naphth-2-yl)-thiazol-3-ium bromide hybrids) and (series III: 3-(4′-[2.2]paracyclophanyl)amido-2-(cyclopropylamino)-4-(naphth-2-yl)thiazol-3-ium bromide) toward the SK-MEL-5 melanoma cell line. Further antiproliferation investigations of **3c** and **3e** on the healthy, normal unaffected SK-MEL-5 cell line indicated their relative safety. Compound **3c** showed an inhibition of eight isoforms of cyclin-dependent kinases (CDK); however, it exhibited the lowest IC_50_ of 54.8 nM on CDK1 in comparison to Dinaciclib as a reference. Additionally, compound **3c** revealed a remarkable downregulation of phospho-Tyr15 with a level (7.45 pg/mL) close to the reference. **3c** mainly showed cell cycle arrest in the pre-G1 and G2/M phases upon analysis of the SK-MEL-5 cell line. The sequential caspase-3 assay for **3c** indicated a remarkable overexpression level. Finally, a molecular docking study was adopted to elucidate the binding mode and interactions of the target compounds with CDK1.

## 1. Introduction

**Figure 1:**
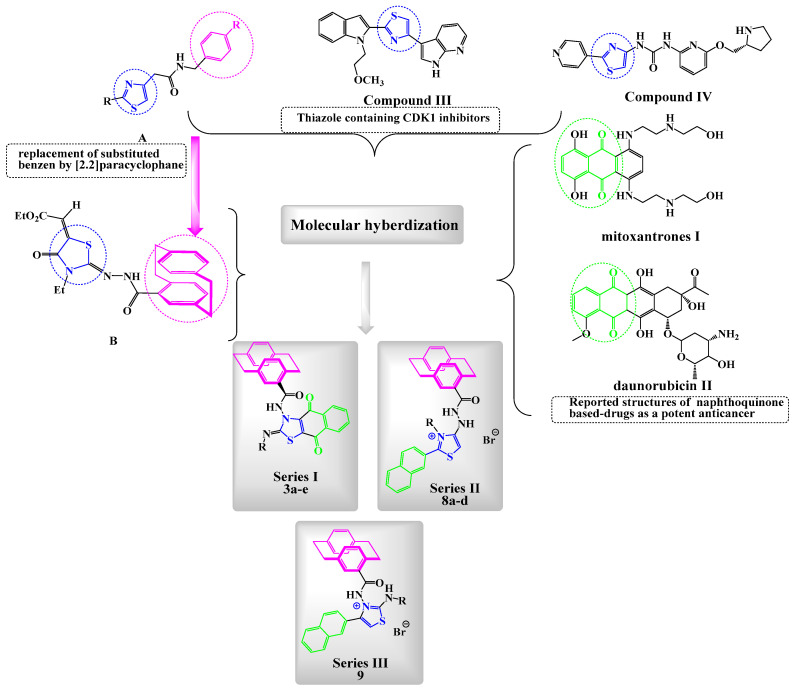
Reported antitumor 1,4-naphthoquinones, thiazoles, and [2.2]paracyclophane derivatives (**I**–**IV**), along with benzylic-thiazoles **A** and paracyclophanylthiazole **B** and the new designed compounds: series I (**3a**–**e**), series II (**8a**–**d**), and series III (**9**). CDK: cyclin-dependent kinases.

1,4-Naphthoquinones are an important scaffold common in the structure of various natural products [1,2,3,4,5], and are present in synthetic compounds that exert diverse biological and pharmaceutical applications [6]. 1,4-Naphthoquinone derivatives are used as antibacterial [7], antifungal [8], and antitumor agents [9]. The chemistry of quinone-annulated heterocycles is highly dependent on the substituents at the quinone showing potent antitumor activity, such as daunorubicin (**I**) and mitoxantrone (**II**) (Figure 1) [10,11]. Pterocaryquinone was isolated from *Pterocarya tonkinesis* and showed activity toward mouth cancer [12]. In searching for agents with better pharmacological properties, a wider reactivity range, and low side effects, it seemed quite promising to incorporate a heterocyclic ring with two heteroatoms (e.g., thiazole) [13]. The thiazole scaffold incorporated in variant therapeutic agents, such as compounds **III**, **IV,** and **B** (Figure 1), has been widely investigated for its antitumor biological effects [14,15], and it was reported that it exhibits antiproliferative activity against different human tumor cell lines, with GI_50_ in the range 0.03–2.38 μM [16]. Additionally, it confined viable cells in the G2/M phase and markedly inhibited the in vitro CDK1 activity [16]. Deregulation of the cell cycle is one of the hallmarks of tumor formation and progression [17]. Human kinases remain interesting targets in oncology; cyclin-dependent kinases (CDKs) are a class of serine/threonine-protein kinases that regulates the temporal progression of cells through the cell cycle [18]. To date, 21 different CDKs (1−11a and 11b−20) have been identified in the human genome, and they can be classified into two main categories based on their primary roles [19,20]. CDK1, CDK2, CDK4, and CDK6 have been discovered to regulate the cell cycle progression upon binding to cyclin proteins. CDK1 forms a complex with cyclin A/B and regulates the phosphorylation of cytoskeleton proteins involved in mitosis [15]. CDK1 is a potential therapeutic target using novel selective small molecule inhibitors of CDK1 [15].

The maturation of the synthetic methodologies for paracyclophanes (PC), with the possibilities for the fine-tuning of structural and functional properties, [2.2]paracyclophane (PC) chemistry has evolved as an interesting class of functional materials [21,22,23]. Aly et al. synthesized heterocycles conjugated to [2.2]paracyclophane, such as a five-membered ring (i.e., imidazolinone and pyrrole) [24,25] and a six-membered ring (i.e., pyridine) [26,27]. Additionally, his group reported on the synthesis of various paracyclophanyl-substituted thiosemicarbazones, thiocarbazones, and thioureas, then studied their complexation towards tridentate and bidentate copper complexes [28,29]. A series of naphthothiazoldiones were synthesized by the reaction of *N*-substituted thioureas with 2,3-dichloro-1,4-naphthoquinone [30,31]. Moreover, naphthothiazole-5-carboxamides were obtained from naphthalimides [32], as well as *N*-substituted-2-(methylamino)naphthoquinones that reacted with S_2_Cl_2_ and DABCO (1,4-diazobicyclooctane) to give 2,3-dihydronaphtho[2,3-*d*][1,3]thiazole-4,9-diones [33]. Despite continuous interest in 1,4-naphthoquinones fused with heterocycles, only a limited number of naphthoquinothiadiazines have been known so far. One of the most important methods in carbon–carbon bond formation is the Eschenmoser-coupling reaction [34]. The reaction of thioamides with mono-haloketones and the preparation of several heterocyclic rings, as well as natural products via the Eschenmoser method, has been also reported [35].

### Compound Design Rationale

Enlightened by the aforementioned information, and in continuation of our efforts in probing for novel effective anticancer agents, we designed and synthesized three series of novel thiazole/paracyclophane conjugates (Figure 1) and investigation them as new CDK1 inhibitors. Series **I** was designed to fuse a naphthoquinone moiety with the thiazole ring to explore more binding with the target enzyme, whereas series II and III bore a naphthyl moiety attached to thiazole with variant substitutions at *N*-thiazole (series II) or position 2 of thiazole (series III). The introduction of different substitutions for the three synthesized series was considered of interest to be a more suitable molecular skeleton aiming at varying the physicochemical properties of the molecules and allowing for a structure–activity relationship exploration study (Figure 1).

It has been demonstrated that the presence of a substituted benzyl ring (i.e., 4-fluoro-, 3,4-dichloro, or 4-methyl) at position 4 of the thiazole compounds increases the critical maximum anticancer activity of the thiazole molecules **A** [36], since the [2.2]paracyclophane molecule has two benzylic moieties and, accordingly, the products can be expected to have increased antitumor cell activity of the obtained products. An increased effect of the [2.2]paracyclophane moiety on the anticancer activity was reported with a series of methyl 2-(2-(4′-[2.2]paracyclophanyl)-hydrazinylidene)-3-substituted-4-oxothiazolidin-5-ylidene)acetates **B** (Figure 1) [37]. It has been reported that the ring composition of drugs containing aromatic rings in proportion to drugs containing one (or two or three or more) aromatic rings in all drugs possessing aromatic rings were elaborately analyzed [38]. The results indicate that, among the drugs possessing aromatic rings, most of the drugs having one/two aromatic rings (all drugs: 79.9%, oral drugs: 78.5%, central nervous system drugs: 86.4%, cardiovascular drugs: 78.9%, and anti-infective drugs: 81.9%), except the anticancer drugs (59.8%) [38]. The statistical results also indicate that candidate drugs with less than four aromatic rings (optimally, one or two, except anticancer drugs, which also tolerate up to three aromatic rings) may possess good drug-like properties and be likely to be developed into approved drugs [38]. Moreover, the *p*-xylene derivative is considered as a half in its structure to [2.2]paracyclophane, so that molecular docking calculations might help to make a comparison. Taking into consideration that the [2.2]paracyclophane moiety has been known by its transannular electronic interaction between its rings [39], however, which was not found in *p*-xylene. Accordingly, to investigate the possible antitumor mechanisms of the synthesized paracyclophanyl-dihydronaphthoquinone[2,3-*d*]thiazoles, CDK1, and other kinases, an enzymatic assay was performed. Docking calculations inside the active site of the CDK1 enzyme were done. A cell cycle analysis and apoptosis through the caspase-3 expression level were screened.

## 2. Results and Discussion

### 2.1. Chemistry

**Scheme 1:**
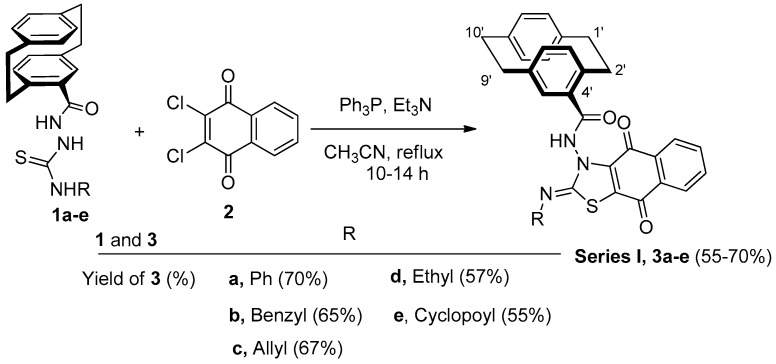
The reaction of **1a**–**f** with **2** under the Eschenmoser condition; synthesis of fused thiazoles **3a**–**e**.

On reacting compounds **1a**–**e** [40] with 2,3-dichloro-1,4-naphthoquinone (**2**) under the Eschenmoser contraction condition (Ph_3_P, Et_3_N, and CH_3_CN) under reflux, the reaction proceeded to give fused thiazoles **3a**–**e** in 55–70% yield (Scheme 1).

**Figure 2:**
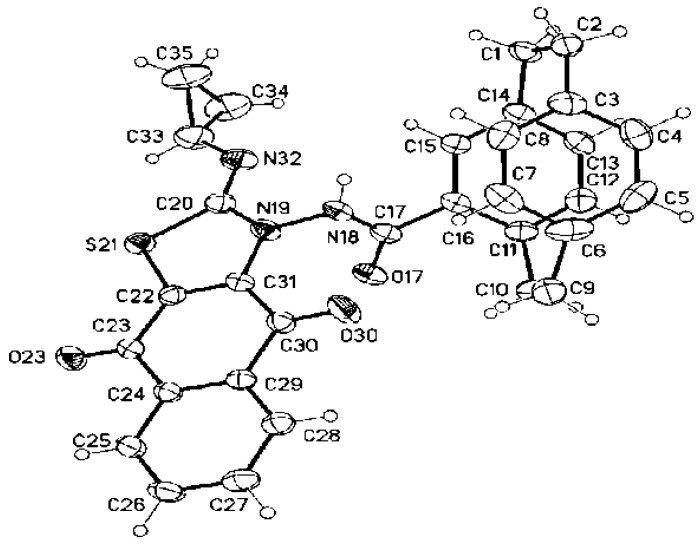
Molecular structure of compound **3e** identified according to IUPAC nomenclature as (*Z*)-*N*-(2-(cyclopropylimino)-4,9-dioxo-4,9-dihydronaphtho[2,3-*d*]thiazol-3(2*H*)-yl)-1,4(1,4)-dibenzo-cyclohexaphane-12-carboxamide.

The structure of the obtained products of **3a–e** was confirmed by IR, NMR, and mass spectra, in addition to an elemental analysis. For example, compound **3e** was obtained as red crystals in 55% yield. Both the mass spectroscopy and elemental analysis confirmed the molecular formula of **3e** as C_31_H_25_O_3_N_3_S. The ^1^H NMR spectrum showed one singlet at δ = 10.12 (amide-NH) and the cyclopropyl protons as three multiplets at δ = 2.58–2.54 (1H), 1.72–1.74 (1H), 1.20-0.47 ppm (3H). The ^13^C NMR spectrum revealed some distinctive carbon signals at δ = 179.4, 176.8 (naphthoquinone-CO), 173.6 (amide-CO), 167.8 (thiazole-C-2), 26.0 (cyclopropyl-CH), 7.9 (cyclopropyl-CH_2_), and 7.5 ppm (cyclopropyl-CH_2_). The X-ray structure analysis confirmed the structure of **3e**, as shown in Figure 2.

**Scheme 2:**
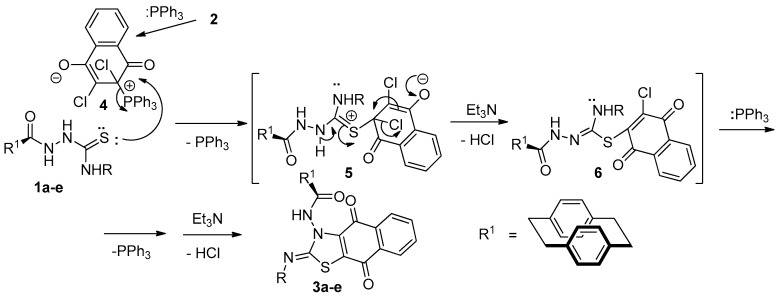
Mechanism describes the formation of compounds **3a–e** under the Eschenmoser contraction condition.

The mechanism would be an initial formation of Zwitter ion salt **4**. Thereafter, a sulfur lone pair in **1a**–**e** would attack the positive carbon in **4**, which would eliminate a molecule of Ph_3_P to give intermediate **5** (Scheme 2). The neutralization and elimination of one molecule of HCl would then initiate by the presence of Et_3_N to give intermediate **6**. Repeating the previous process by the attachment of a molecule of Ph_3_P and elimination of the second molecule of HCl initiated by Et_3_N would give compounds **3a**–**e** (Scheme 2).

#### Synthesis of Substituted Thiazoles **8a**–**d** and **9**

**Scheme 3:**
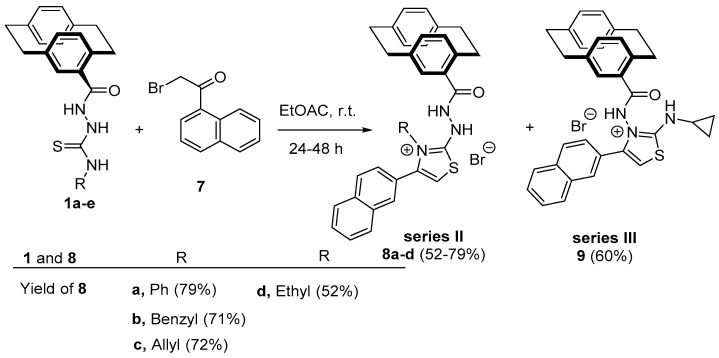
The reaction of **1a**–**e** with **7**; the synthesis of thiazole derivatives **8a–d** and **9**.

On reacting compounds **1a–e** with 2-bromo-1-(naphth-1-yl)ethanone (**7**) in ethyl acetate and at room temperature, the reaction proceeded to give compounds **8a–d** with the corresponding derivatives of **1a–d**. On the other hand, the reaction of compound **1e** with **7** gave the regioisomer **9** in 60% yield (Scheme 3).

**Figure 3:**
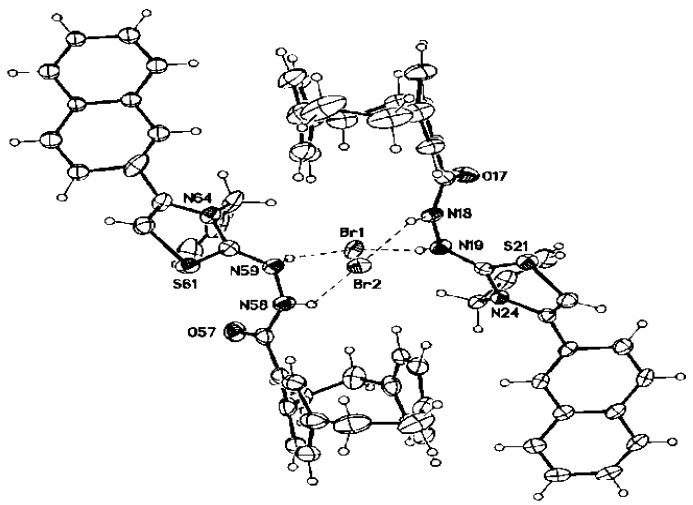
Molecular structure of the dimer of compound **8c** identified according to IUPAC nomenclature as the 2-(2-(1,4[1,4]-dibenzenacyclohexaphane-1^2^-carbonyl)hydrazinyl)-3-allyl-4-(naphth-2-yl)thiazol-3-ium bromide.

In the case of compound **8c**, the ^1^H NMR spectrum showed three singlets at δ = 11.23, 7.35, and 6.95 ppm corresponding to amide-NH, thiazole-CH-5, and hydrazinyl-NH, respectively. The allyl protons resonated as three multiplets at δ = 5.89–5.80, 5.32–5.00, and 4.84–4.70 ppm for allyl-CH=, allyl-CH_2_=, and allyl-CH_2_-, respectively (see the Experimental section). The ^13^C NMR spectrum confirmed the structure of **8c** by the appearance of carbon signals at δ = 173.8 (amide-CO), 168.2 (thiazole-C-2), 143.4 (thiazole-C4), 133.5 (allyl-CH=), 118.9 (allyl-CH_2_=), 108.3 (thiazole-CH-5), and 50.4 ppm (allyl-CH_2_). The structure of **8c** was confirmed by the X-ray structure analysis, as shown in Figure 3.

**Figure 4:**
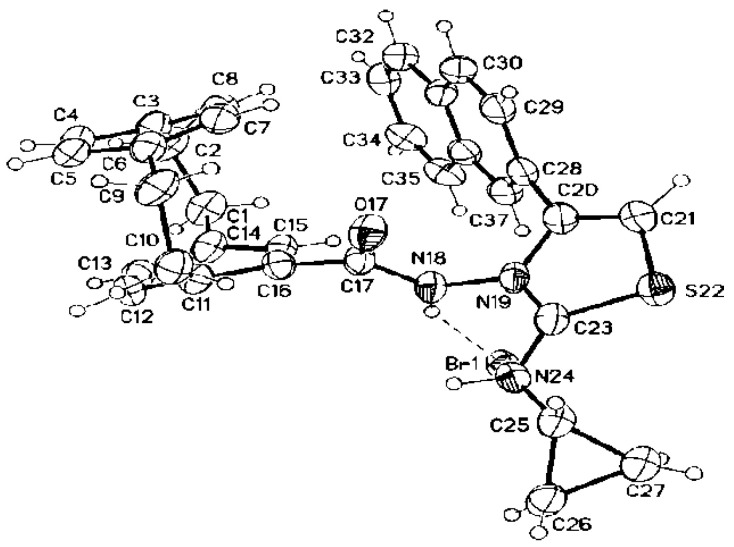
Molecular structure of compound **9** identified according to IUPAC nomenclature as 3-(1,4(1,4)-dibenzenacyclohexaphane-1^2^-carboxamido)-2-(cyclopropylamino)-4-(naphth-2-yl)thiazol-3-ium bromide.

In compound **9**, the ^1^H NMR spectrum revealed three singlets at δ = 11.79, 7.47, and 5.36 related to amide-NH, thiazole-CH, and NH-amine, respectively. The cyclopropyl protons resonated as three multiplets at δ = 2.74–2.64 (1H), 1.04–0.95 (cyclopropyl-CH_2_), and 0.81–0.75 (cyclopropyl-CH_2_). The ^13^C NMR spectrum showed the carbon signals of CO-amide, thiazole-C-2, thiazole-C-4, and thiazole-CH-5 at δ = 172.0, 166.1, 142.4, and 104.3 ppm, respectively. The cyclopropyl protons resonated at δ = 28.2 (cyclopropyl-CH), 7.3 (cyclopropyl-CH_2_), and 7.2 ppm (cyclopropyl-CH_2_). The structure of compound **9** was confirmed by the X-ray structure analysis, as shown in Figure 4.

**Scheme 4:**
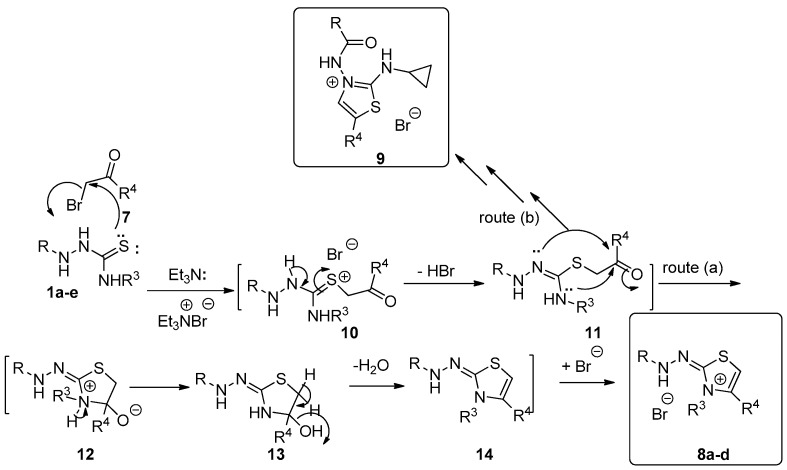
Mechanism describes the formation of compounds **8a**–**d** and **9**.

The mechanism that describes the formation of **8a**–**d** (Scheme 4) depends on an initial addition of the sulfur lone pair to the electrophilic carbon of **7** to produce Zwitter ion salt **10**. The elimination of a molecule of HBr from intermediate **10** would give **11**. Two routes were then proposed as routes (a) in which the cyclization process occurred from the amine-NH to the carbonyl carbon to give salt **12**. The neutralization of **12** would give intermediate **13** (Scheme 4). The elimination of water from **13** would give **14**, which stabilized by abstracting the liberating HBr to form products **8a**–**d** (Scheme 4). Route (b) describes the other type of cyclization process that occurred to intermediate **11** by the hydrazinyl-NH lone pair. On repeating the previously mentioned steps, compound **9** would be formed (Scheme 4).

### 2.2. Biological Activity Evaluation

#### 2.2.1. In Vitro Screening of One-Dose Anticancer Activity on 60 Cancer Cell Lines

Among the synthesized compounds, **3a**–**e** and **8a**–**d** were chosen by the National Cancer Institute (NCI) according to the protocol of the Drug Evaluation Branch of the National Cancer Institute, Bethesda, MD, USA for the in vitro anticancer screening on 60 cancer cell lines. The methodology of the NCI anticancer screening has been described in detail elsewhere (http://www.dtp.nci.nih.gov). Tested compounds were added to the culture at a single concentration (10^−5^ M), and the cultures were incubated for 48 h. Endpoint determinations were made with a protein-binding dye, sulforodamine B (SRB). The results for each tested compound were reported as the percentage of growth of the treated cells when compared to the untreated control cells. The percentage of growth was evaluated spectrophotometrically versus controls not treated with test agents. All experiments were repeated three times. The NCI results of the naphthothiazole/paracyclophane conjugates recorded in Table 1 indicate that five compounds **3a**–**e** displayed very potent anticancer activity with complete cell death (% of growth inhibition ≥ 100%) on most of the tested cancer cell lines. Interestingly, compounds **3c** and **3d** showed complete cell death on all nine cancer cell panels in a range of 103.65–198.49% and 103.66–197.05%, respectively. Additionally, compound **3e** showed complete cell death on three cancer cell panels (colon cancer, CNS cancer, and melanoma) of the range 109.10–193.24%. Compounds **3a** and **3b**, as well as **8c**, had complete cell death on only one panel (melanoma) of the range 110.14–172.49%, while they exhibited moderate cytotoxic activity toward the other tested cell lines. On the other hand, compounds **8a**, **8b**, and **8d** possessed lesser activity. Compound **8d** had significant cytotoxicity against melanoma SK-MEL-5 and breast cancer T-47D, with inhibition % of 83.18% and 75.60%, respectively. However, it showed moderate activity against leukemia RPMI-8226 and colon cancer HCT-15, with cell growth inhibition (%) of 62.22% and 59.89%, respectively. Moreover, compound **8b** had moderate inhibition against leukemia K-562, colon cancer HCT-15, and prostate cancer PC-3, with an inhibition percent of 54.53%, 63.36%, and 54.49%, respectively. Meanwhile, compound **8a** displayed weak cell growth inhibition activity against most of the tested cancer cell lines.

Regarding the results recorded in Table 1, it can be deduced that the replacement of the naphthyl group by the naphthoquinone group, as in compounds **3a**–**e**, gave the fascinating outstanding anticancer activity on the tested cancer cell lines. This could be attributed to increasing the binding to the target protein due to the presence of an extra two oxygen groups in the quinone moiety, and this encouraged us to further test them throughout the molecular docking study.

#### 2.2.2. In Vitro Five-Dose Full NCI 60 Cell Panel Assay

Compounds **3c**, **3d**, and **3e** achieved complete cell death at most cancer cell lines and were selected for advanced five-dose testing against the full panel of 60 human tumor cell lines. All the 60 cell lines representing nine tumor subpanels were incubated at five different concentrations (0.01, 0.1, 1, 10, and 100 µM). The outcomes were used to create log concentrations versus % growth inhibition curves, and three response parameters (GI_50_, TGI, and LC_50_) were calculated for each cell line. The GI_50_ value (growth inhibitory activity) corresponds to the concentration of the compound causing a 50% decrease in net cell growth, the TGI value (cytostatic activity) is the concentration of the compound resulting in the total growth inhibition (TGI), and the LC_50_ value (cytotoxic activity) is the concentration of the compound causing a net 50% loss of initial cells at the end of the incubation period of 48 h. From the results in Table 2, it is clear that compound **3d** exhibited remarkable anticancer activity against most of the tested cell lines, representing nine different subpanels. Compound **3d** showed high activity against most of the tested cell lines, with the GI_50_ ranging from 1.85 to 9.98 mM (Table 2). The criterion for selectivity of a compound depends upon the ratio obtained by dividing the full-panel MID (the average sensitivity of all cell lines toward the test agent) (µM) by their subpanel MID (µM). Ratios between 3 and 6 refer to moderate selectivity; ratios > 6 indicate high selectivity toward the corresponding cell line, while compounds not meeting either of these criteria rated nonselective. In this context, compound **3d** was found to have broad-spectrum antitumor activity against the nine tumor subpanels tested, with selectivity ratios ranging between 0.73 and 1.14 at the GI_50_ level. Furthermore, compound **3e** exhibited remarkable anticancer activity against most of the tested cell lines representing nine different subpanels and showed high activity against most of the tested cell lines, with the GI_50_ ranging from 1.31 to 9.66 mM (Table 2). Compound **3e** was found to have broad-spectrum antitumor activity against the tested nine tumor subpanels, with selectivity ratios ranging between 0.98 and 1.43 at the GI_50_ level. On the other hand, compound **3c** revealed broad-spectrum cell growth inhibition activity against the major of the tested tumor subpanels, with GI_50_ values ranging from 1.13 to 5.77 µM and the selectivity ratio ranging from 0.78 to 1.32. It can be deduced that compounds **3c**, **3d**, and **3e** possessed broad-spectrum antitumor agents against different tested tumor subpanels with no selectivity toward the tested cell lines.

#### 2.2.3. Evaluation of In Vitro Antiproliferative Activities against Melanoma SK-MEL-5 Cancer Cell Line and Nontumorigenic SK-MEL-5 Cell Line

**Figure 5:**
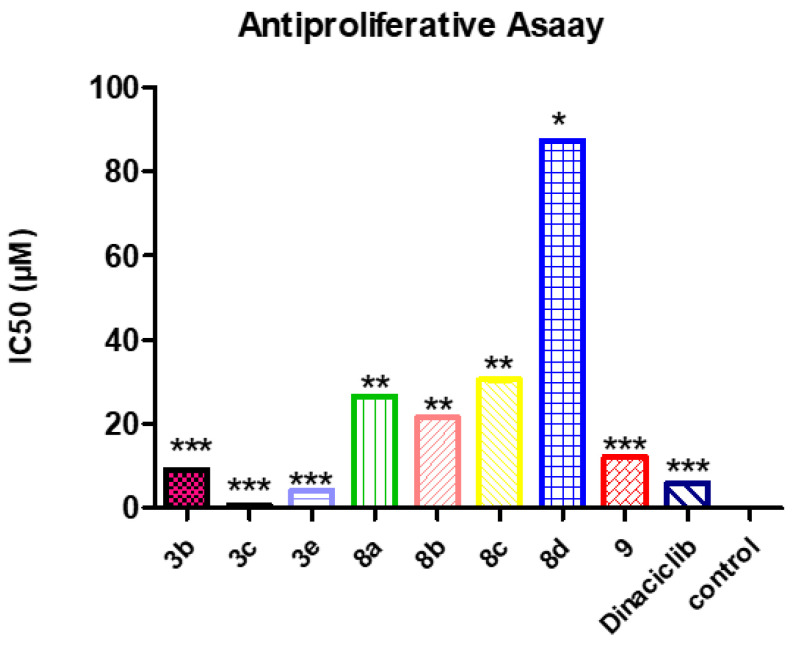
3-(4,5-Dimethylthiazol-2-yl)-2,5-diphenyltetrazolium bromide (MTT) assay of **3b, 3c, 3e, 8a**–**d, 9,** and Dinaciclib on the melanoma SK-MEL-5 cell line relative to the control. IC_50_: inhibitory concentration (50%). Results indicating at *, **, *** means less significant.

The synthesized compounds **3b**, **3c**, **3e**, and **8a**–**d**, as well as Dinaciclib (the reference compound), were evaluated for their antiproliferative activity by being treated at a concentration of 50 μM (Table 3). The antiproliferative assay was performed with melanoma SK-MEL-5 cells, whereas most of our goal compounds exhibited the highest potency; the assay was done for four days and, then, the 3-(4,5-dimethylthiazol-2-yl)-2,5-diphenyltetrazolium bromide (MTT) assay. GraphPad Prism software (GraphPad Software, San Diego, CA, USA) was used to calculate the median inhibition concentration (IC_50_) for the tested compounds (Figure 5). The difference in the results was considered significant when the values of *p* were less than 0.05.

As shown in Table 3, the most active three paracyclophane/thiazole conjugates bearing the naphthoquinone moiety, **3b**, **3c,** and **3e**, exhibited potent-to-remarkable proliferation inhibition of cancer cells, with IC_50_ of 9.11, 0.81, and 4.18 µM compared to Dinaciclib with an IC_50_ of 5.97 µM. On the other hand, all other compounds bearing the naphthyl moiety instead showed moderate activity against the growth of the cancer cell line. From these results, we can conclude that the presence of the naphthoquinone moiety improve binding with the target protein, in addition to substitution with the benzyl, allyl, or cyclopropyl groups, which would enhance the antiproliferative activity, especially upon increasing the flexibility of the compounds (as in compounds **3c** and **3e**); this extension increased the antiproliferation potency of these compounds against melanoma SK-MEL-5. It is interesting to mention that the proliferation inhibitory results were positively correlated with the anticancer results obtained from the NCI on the tested cancer cell lines.

According to the screening results, and to better explore the structure-activity relationship (SAR) of the compounds, besides the biochemical assay (IC_50_) on the SK-MEL-5 cancer cell line, we also evaluated the compound’s cellular antiproliferative activity, using the WI38 cell line normal lung cells of a three-month-gestation aborted female fetus to monitor the general cytotoxicity as well (Table 4). Independently, compounds **3c** and **3e** showed the lowest IC_50_ values among the tested compounds against the SK-MEL-5 leukemia cancer cells; therefore, we were encouraged to select **3c** and **3e** for further investigation for its antiproliferation on normal, healthy, unaffected cell lines by MTT assay (Table 4).

Compounds **3c** and **3e** achieved IC_50_ values of 32.59 and 39.86 µM, respectively, on the selected normal WI38 cell line, which is greater than of the reference Dinaciclib IC_50_ = 22.01 µM. The gained results indicated the relative safety of the tested compounds on normal cells; they also showed a good selectivity window between normal cells and cancer cells.

##### Structure-Activity Relationship

Based on the previous results, we can deduce that when the [2.2]paracyclophane/thiazole conjugated with the naphthoquinone moiety, as in series I, it exhibited better antitumor activity than their naphthyl-containing congeners (series II and series III). This could be attributed to the specific interaction with the proposed CDKK1 enzyme that is absent in series II and III, hence, revealed weak activity. Meanwhile, the antiproliferation toward the melanoma SK-MEL-5 cell line concerning series I showed the highest potency upon substitution of R on thiazole imine with small molecules such as allyl, ethyl, and cyclopropyl; however, when R was replaced with phenyl and benzyl, the activity was particularly attenuated, which may be probably due to their steric hindrance effects. Since the target compounds **3a**–**e** revealed interesting antitumor activity, this work underwent further enzymatic mechanistic investigations to prove exactly the explained hypothesis.

### 2.3. Selectivity Profiling of Compound **3c**

**Figure 6:**
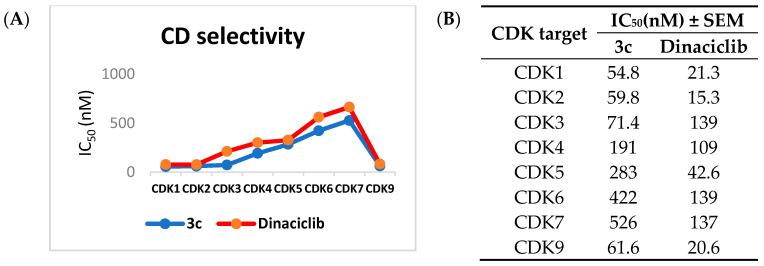
Selectivity profiling of compound **3c** and Dinaciclib. (**A**,**B**) Biochemical testing of **3c** against CDK isoforms on the SK-MEL-5 cell line. All data were obtained by triplet testing.

Given the fact that **3c** exhibited the best in vitro biochemical activity against SK-MEL-5, the antiproliferative efficacy in cancer cell lines, and to investigate whether the antiproliferative activities of **3c** was related to the interaction with CDK may play critical roles in the regulation of the cell cycle or/and transcription. We chose this compound for further selectivity evaluations toward different kinases. We first subjected **3c** to examine the selectivity among eight different CDK isoforms that are available (CDK1, 2, 3, 4, 5, 6, 7, and 9) in comparison to the reference Dinaciclib using a Kinase-Glo^®^ MAX kit and incubated the tested compounds at 30 °C for 45 min [36] (Figure 6).

Interestingly, **3c** potently inhibited the CDK kinase, showing IC_50_ in nanomolar values. Moreover, it exhibited the selectivity toward CDK1, 2, and 9 with an IC_50_ of 54.8, 59.8, and 61.6 nM in comparison to Dinaciclib with an IC_50_ of 21.3, 15.3, and 20.6 nM, indicating a more than 10-fold selectivity over CDK3, 4, 5, 6, 7. It is noteworthy that compound **3c** revealed the best selectivity toward CDK1, with the lowest IC_50_ of 54.8 nM (Figure 6).

### 2.4. Inhibition of Phospho-CDK1/CDC2 Cell-Based Phosphorylation in SK-MEL-5 Cancer Cells

As it is understood by those skilled in the drug discovery art, kinase inhibitors should possess both high affinities for the target kinase, as well as high selectivity versus other protein kinases. Therefore, we next investigated the cellular mode of action of the most potent tested inhibitor **3c** using an anti-CDC2 (phospho-Tyr15) antibody *via* the Enzyme-Linked Immunosorbent Assay (ELISA) assay method to show the capability of **3c** to downregulate the CDK1-phosphorylated substrate and the loss of cyclin expression in treated cells. The anti-CDC2 (phospho-Tyr15) antibody is a rabbit polyclonal antibody. The treatment of SK-MEL-5 cells with **3c** for a period of 24 h showed a reduction of phosphorylation at Tyr15.

**Figure 7:**
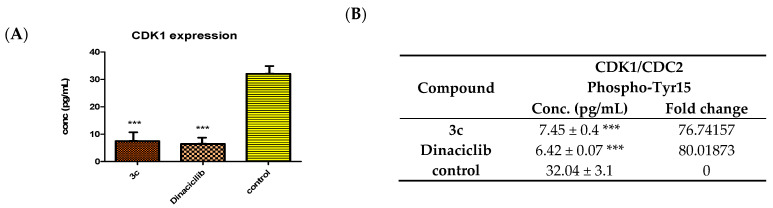
Effect of **3c** and Dinaciclib on CDK1/CDC2 phospho-Tyr15 regulation in the SK-MEL-5 cell line. (**A**) Enzyme-Linked Immunosorbent Assay (ELISA) for the immunogen phosphor-peptide for **3a** and Dinaciclib and nonphosphopeptide for the control group using the anti-CDC2 (phospho-Tyr15) antibody. (**B**) CDK1/CDC2 phospho-Tyr15 inhibition (conc. (pg/mL) ± SEM) of compound **3c** and Dinaciclib. All data were obtained by triplet testing. Results were significantly different from the control at *** *p* < 0.05.

Figure 7 illustrates that compound **3c** revealed a significant downregulation of phospho-Tyr15 with a level of 7.45 pg/mL, which is close to the reference inhibitor (6.42 pg/mL) in comparison to the control group (32.04 pg/mL). Compound **3c** caused a CDK1 phospho-Tyr15 down-expression level about 76.74-fold change comparable to the reference (80.02-fold change) relative to the control (Figure 7), which confirmed cellular CDK1 inhibition.

### 2.5. Cell Cycle Analysis

**Figure 8:**
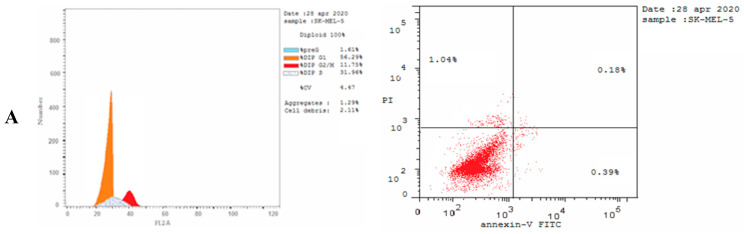

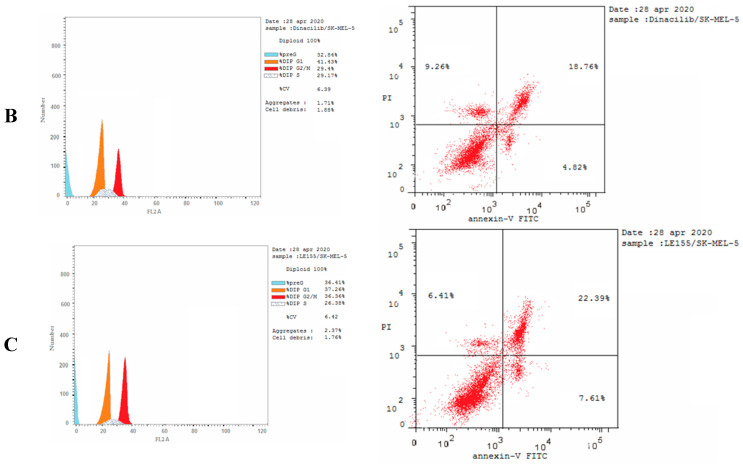
Cell cycle analysis and apoptosis induction analysis against SK-MEL-5 using annexin V/PI at IC_50_. Concentrations representing the growth arrest at the pre-G1 (G0) and G2/M phases. (**A**) Untreated cells, (**B**) treated cells with Dinaciclib, and (**C**) treated cells with **3c**. The test was repeated three times, and **3c** and the reference were incubated for 24 h (2 × 10^5^ cells/well) at 37 °C.

**Figure 9:**
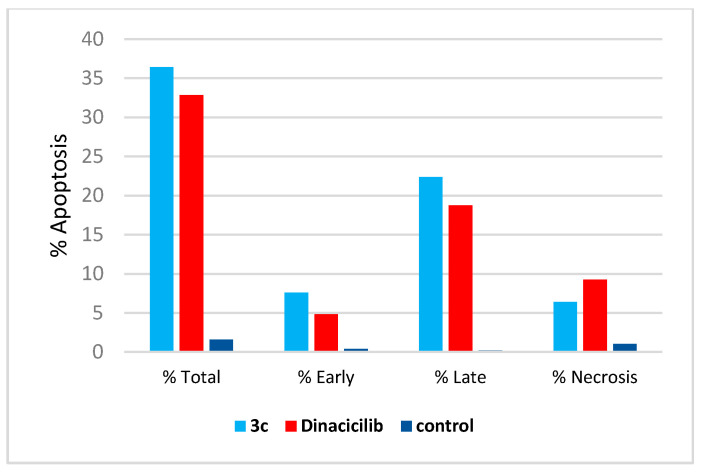
Percentage of apoptosis and necrosis for compound **3c** on **the** SK-MEL-5 melanoma cell line.

**Figure 10:**
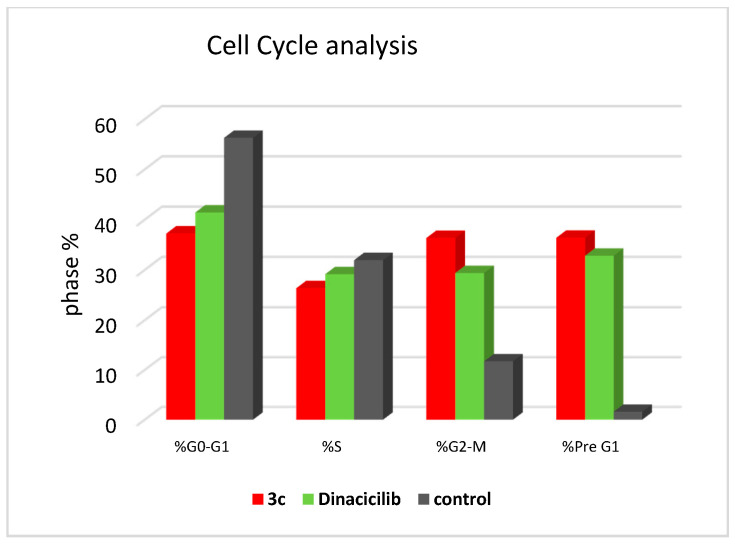
Cell cycle analysis on the SK-MEL-5 melanoma cell line treated with the compound.

The cell cycle analysis was carried out for the most potent compound **3c** against the SK-MEL-5 melanoma cancer cell line. The assay was carried out using cytometers, which are Becton Dickinson Immuno-Cytometry Systems, Beckman/Coulter Inc., DACO/cytomation, and PARTEC GmbH, Brea, CA 94043 USA. The results of the annexin V/PI flow cytometry of SK-MEL-5 cells were repeated three times after treatment with an IC_50_ value (0.81 µM) of **3c**. The results showed that the percentage of cells of SK-MEL-5 in the G0/G1 phase of the cell cycle in the control was 56.29%, which is remarkable to a Dinaciclib of 41.43%, and recorded a remarked decrease to 37.26% upon treatment with **3c** (see Table S1, Supplementary Data). The G2/M phase exhibited a noteworthy percent increase that reached 36.36% because of the cell accumulation at this phase. Moreover, the apoptotic cell percentage for the phase pre-G1 was raised from 1.61% for the untreated control to 36.41% and 32.84% in comparison to treated cells with compound **3c** and Dinaciclib, respectively, (upper right quadrant of the cytogram) (Figure 8). The gained results indicated that the late-apoptosis percent was greater than that of early apoptosis, which was considered a good sign for irreversible apoptosis (Figure 9 and Figure 10). According to the above results, it is clear that compound **3c** exhibited pre-G1 apoptosis and cell cycle arrest at the G2/M phase. Therefore, the results revealed that the tested compound was not cytotoxic but antiproliferative, causing programmed cell death and cell cycle arrest.

### 2.6. Caspase-3 Activation Assay

**Figure 11:**
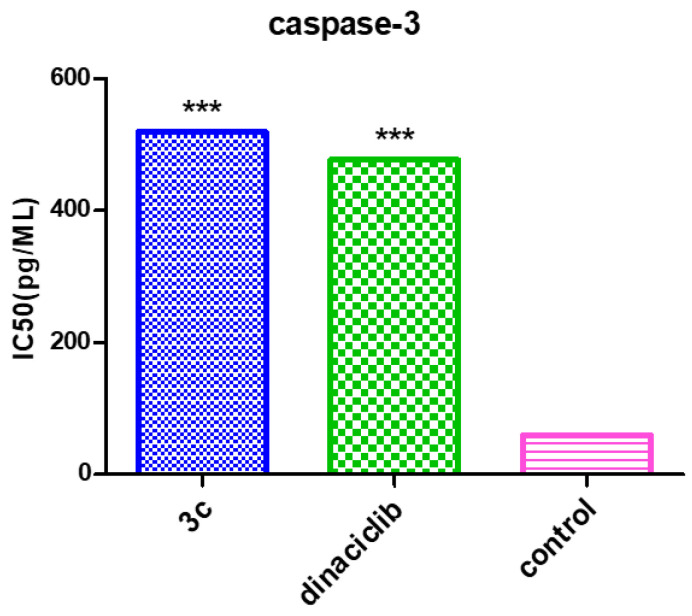
Caspase-3 levels of **3c** and Dinaciclib on the SK-MEL-5 line relative to the control. Results indicating at *** means less significant.

The intracellular caspase-3 signaling was studied using the ELISA analysis method and replicated three times to understand the molecular mechanisms by which **3c** induces G2/M phase arrest. Caspase-3 is essential for the apoptotic signal spreading after exposure to the antimitotic compounds [37], the effect of paracyclophane/thiazole conjugate **3c** on the caspase-3 activated enzyme. Compound **3c** was evaluated against the SK-MEL melanoma cancer cell line at concentrations of 2500, 1250, 625, 313, 156, 78, and 39 pg/mL. Table 5 displays the results that revealed that compound **3c** possessed a remarkable overexpression of the caspase-3 protein level (519.4 pg/mL), which is compared to the reference Dinaciclib (476.7 pg/mL). Compound **3c** caused overexpression of the caspase-3 protein level about 8.66-fold change higher than the reference (7.95-fold change) relative to the control (Figure 11). Hence, it could be deduced from the above results that apoptosis may be attributed to the overexpression of caspase-3, which was induced by the tested compounds.

### 2.7. Molecular Modeling

**Figure 12:**
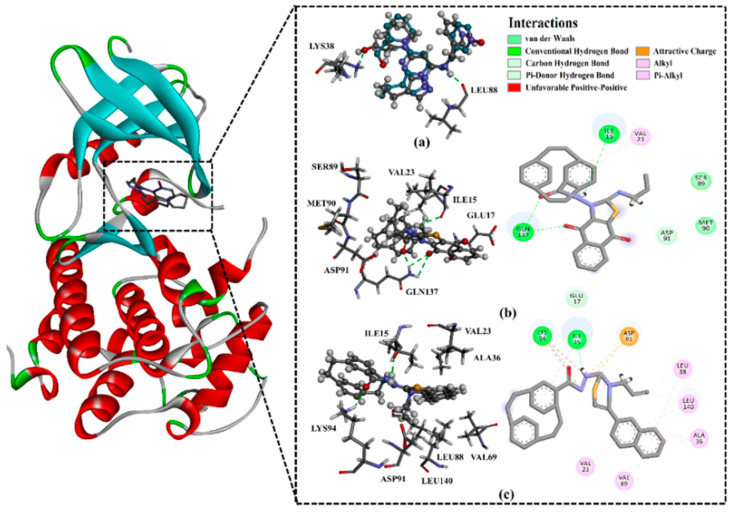
(**a**) 3D representation of the predicted binding mode (in grey) and experimental structure (in cyan) of Dinaciclib, (**b**) 2D and 3D representations of the binding pose and ligand interactions of **3c**, and (**c**) 2D and 3D representations of the binding pose and ligand interactions of **8c** with the CDK1 receptor (PDB: 6GU6).

Before docking calculations, validation of the employed Autodck4.2.6 parameters and protocol were first performed based on the available experimental data. The co-crystallized Dinaciclib in the complex with CDK1 (PDB code: 6GU6) was redocked, and the binding mode was predicted and compared to the experimental structure (Figure 12).

A comparison of the predicted docked structure with the corresponding resolved crystal structure revealed that the Autodock4.2.6 with the employed parameters accurately predicted the correct binding mode of Dinaciclib inside the active site of CDK1, forming two essential hydrogen bonds with LYS38 and LEU88 (Figure 12). The estimated root mean square deviation (RMSD) between the experimental and docked structures was 0.2 Å.

Utilizing the molecular docking, the binding modes and affinities of the synthesized compounds **3a**–**e, 8a**–**d**, and **9** with the CDK1 active site were then investigated. The calculated docking scores for the synthesized compounds are listed in Table S2. As can be seen from Table S2, all synthesized compounds gave good binding affinities towards CDK1, with values in the range −8.6 to −9.5 kcal/mol, relative to that of Dinaciclib (−10.6 kcal/mol), supporting that CDK1 inhibition is a plausible mechanism explaining the antitumor activity observed with those compounds. Compared to compounds **8a**–**d**, there were better binding affinities with CDK1 for compounds **3a**–**e** containing the naphthoquinone scaffold, showing the specific interaction of the oxygen atom with the amino acid residue GLN137 (Figure 12). This suggests the moderately higher antitumor activity than the other derivatives **8a**–**d** and **9** lacking the naphthoquinone scaffold.

Among the paracyclophane/thiazoles-naphthoquinones conjugates **3a**–**e**, compound **3c** showed the highest binding affinity, with a docking score of −9.5 kcal/mol, forming four hydrogen bonds with GLN137 (1.88, 2.65, and 2.95 Å) and ILE15 (2.78 Å) (Figure 12). Besides, pi-based and hydrophobic interactions between **3c** and the key amino acid residues inside the active site were also observed (Figure 12).

The contribution of the paracyclophane moiety to inhibit CDK1 is questionable. To address the answer, *p*-xylene analogs of compounds **3a**–**e** were modeled, and their binding scores with CDK1 were predicted (Table S3). According to the data listed in Table S3, the *p*-xylene analogs showed relatively lower docking scores, with values in the range of −8.1 to −8.7 kcal/mol, compared to paracyclophane-based **3a**–**e** (docking scores of −9.3 to −9.8 kcal/mol), confirming the favorable contribution of the paracyclophane moiety in pi-based interactions with the CDK1 active site.

## 3. Experimental

### 3.1. Chemistry

The IR spectra were recorded by the ATR technique (ATR = attenuated total reflection) with an FT device (FTIR Bruker IFS 88, Middlesex County, MA, USA), Institute of Organic Chemistry, Karlsruhe Institute of Technology, Karlsruhe, Germany. The NMR spectra were measured in DMSO-*d_6_*, chloroform-*d*, and acetone-*d_6_* on a Bruker AV-400 spectrometer 400 MHz for ^1^H and 100 MHz for ^13^C, and the chemical shifts were expressed in δ (ppm) versus internal tetramethylsilane (TMS) = 0 for ^1^H and ^13^C and external liquid ammonia = 0 at in the Chemistry Department, Florida Institute of Technology, 150WUniversity Blvd, Melbourne, FL 32901, USA. The description of the signals includes: s = singlet, d = doublet, t = triplet, q = quartet, m = multiplet, dd = doublet of doublet, and ddd = doublet of dd. Mass spectra were recorded on a FAB (fast atom bombardment) Thermo Finnigan Mat 95 (70 eV) and ESI (electrospray ionization-mass spectrometry, Florida Institute of Technology, 150WUniversity Blvd, Melbourne, FL 32901, USA) Thermo-Fisher QExactive Plus. For the high-resolution mass (HRMS), the following abbreviations were used: calc. = theoretically calculated mass and found = mass found in the analysis, Institute of Organic Chemistry, Karlsruhe University, Karlsruhe, Germany. TLC was performed on analytical Merck 9385 silica aluminum sheets (Kieselgel 60)Aldrich, USA with a Pf_254_ indicator; TLC were viewed at λ_max_ = 254 nm. Crude products were purified by flash chromatography with Silica gel 60 (0.040 × 0.063 mm; Geduran^®^) (Merck, Kenilworth, NJ, USA).

#### 3.1.1. Starting Materials

*N*-Substituted [2.2]paracyclophenylhydrazinecarbothioamides **1a-e** were prepared according to the literature [40], whereas compounds **2** and **4** were bought from Aldrich (St. Louis, MO, USA).

#### 3.1.2. Reactions of Hydrazinecarbothioamides **1a**–**e** with DCHNQ (**2**); Preparation of Compounds **3a**–**e**

2,3-Dichloro-1,4-naphthoquinone 0.250 g (**2**, 1.1 mmol, 1.10 equiv.) was added to a stirred solution of **1a**–**e** (1.0 mmol, 1.00 equiv.) in 25 mL dry CH_3_CN. The resulting solution was stirred at room temperature for 16 h. After *S*-alkylation was completed, and the dried salt was dissolved in dry CH_3_CN; after which, triethylamine (1.1 mmol) and triphenylphosphine (1.1 mmol) were added. The resulting mixture was left under reflux for about 8–10 h. The reaction mixture was then left to cool at room temperature; then, H_2_O (50 cm^3^) was added. The resulting solution was extracted with CH_2_Cl_2_. The organic extracts were dried over anhydrous CaCl_2_, filtered, and evaporated under reduced pressure. The crude was purified by flash chromatography by using cyclohexane/ethyl acetate (10:4) as the eluent to afford **3a**–**e**.

#### 3.1.3. (*Z*)-*N*-(4,9-Dioxo-2-(phenylimino)-4,9-dihydronaphtho[2,3-*d*]thiazol-3(2*H*)-yl)-4′-[2.2]paracyclophanylamide (**3a**)

Red crystal (methanol), yield: 0.390 g (70%). ^1^H NMR (400 MHz, acetone-*d*_6_, ppm) δ = 10.41 (s, 1H, amide-NH), 8.20–8.09 (m, 2H, Ar-H), 7-92–7-87 (m, 2H, Ar-H), 7.48–7.42 (m, 1H, Ph-H), 7.23–7.05 (m, 4H, Ph-H), 6.85–6.77 (m, 2H, PC-H), 6.70–6.56 (m, 5H, PC-H), 3.87–3.79 (m, 1H, PC-CH_2_), 3.27–3.20 (m, 1H, PC-CH_2_), 3.14–3.05 (m, 3H, PC-CH_2_), 2.98–2.85 (m, 3H, PC-CH_2_). ^13^C NMR (100 MHz, acetone-*d*_6_, ppm) δ = 177.4, 174.2 (naphthoquinone-CO), 167.9 (amide-CO), 154.3 (thiazole-C2), 150.5 (Ph-C), 142.1 (PC-C-6′), 141.8 (PC-C-11′), 141.1 (PC-C-14′), 140.9 (PC-C-3′), 140.7, 140.2 (Ar-C), 140.1 (PC-CH), 137.2 (Ar-C), 136.9, 135.2, 134.9, 133.9 (Ar-CH), 133.7 (PC-C-4′), 133.6, 133.3, 133.1, 132.9 (PC-CH), 132.8, 132.7, 130.7 (Ph-CH), 127.7, 126.7 (PC-CH), 125.4, 121.5 (Ph-CH), 108.3 (Ar-C), 35.8 (PC-CH_2_-1′), 35.6 (PC-CH_2_-10′), 35.5 (PC-CH_2_-9′), 35.3 (PC-CH_2_-2′). IR (ATR, cm^−1^) *ṽ* = 3325–3165 (w, NH), 2925 (s, Ar-CH), 2847 (s, aliph-CH) 1669, 1632, 1587 (CO), 1567 (C=N). MS (FAB) *m*/*z* (%) = 556.2 [M + H]^+^ (50). HRMS (FAB; [M + H]^+^, C_34_H_26_O_3_N_3_^32^S_1_) Calc.: 556.1695, Found: 556.1694.

#### 3.1.4. (*Z*)-*N*-(2-(Benzylimino)-4,9-dioxo-4,9-dihydronaphtho[2,3-*d*]thiazol-3(2*H*)-yl)-4′-[2.2]paracyclophanylamide (**3b**)

Red crystal (methanol), yield: 0.370 g (65%). ^1^H NMR (400 MHz, DMSO-*d_6_*, ppm) δ = 11.26 (s, 1H, amide-NH), 8.10–8.01 (m, 2H, Ar-H), 7.90–7.84 (m, 2H, Ar-H), 748–7.02 (m, 5H, Ph-H), 6.78–6.37 (m, 7H, PC-H), 4.12 (s, 2H, CH_2_Ph), 3.26–2.67 (m, 8H, PC-CH_2_). ^13^C NMR (100 MHz, DMSO-*d_6_*, ppm) δ = 178.8, 176.2 (naphthoquinone-CO), 174.7 (amide-CO), 173.0 (thiazole-C2), 140.5 (Ph-C), 139.5 (PC-C-6′), 139.0 (PC-C-11′), 138.9 (PC-C-14′), 138.2 (PC-C-3′), 135.7, 135.6 (Ar-C), 134.6 (Ar-C), 134.5, 134.2, 132.7, 132.5 (Ar-CH), 132.4 (PC-C-4′), 132.0, 131.6, 131.5, 131.4, 131.3 (PC-CH), 128.2, 128.1, 127.8, 127.3, 127.2 (Ph-CH), 127.0, 126.6 (PC-CH), 126.1 (Ar-C), 57.3 (CH_2_Ph), 34.8 (PC-CH_2_-1′), 34.6 (PC-CH_2_-10′), 34.5 (PC-CH_2_-9′), 34.4 (PC-CH_2_-2′). IR (ATR, cm^−1^) *ṽ* = 3407–3288 (w, NH), 2917 (s, Ar-CH), 2851 (s, aliph-CH) 1681, 1649, 1585 (CO), 1511 (C=N). MS (FAB) *m*/*z* (%) = 570.3 [M + H]^+^ (65). HRMS (FAB; [M + H]^+^, C_35_H_28_O_3_N_3_^32^S_1_) Calc.: 570.1851, Found: 570.1854.

#### 3.1.5. (*Z*)-*N*-(2-(Allylimino)-4,9-dioxo-4,9-dihydronaphtho[2,3-*d*]thiazol-3(2*H*)-yl)-4′-[2.2]paracyclophanylamide (**3c**)

Red crystal (methanol), yield: 0.350 g (67%). ^1^H NMR (400 MHz, DMSO-*d*_6_, ppm) δ = 11.28 (s, 1H, amide-NH), 8.42–7.76 (m, 4H, Ar-H), 7.03–6.70 (m, 2H, PC-H), 6.64–6.35 (m, 5H, PC-H), 6.06–5.65 (m, 1H, allyl-CH=), 5.43–4.97 (m, 2H, allyl-CH_2_), 4.05–3.78 (m, 2H, allyl-CH_2_=), 3.65–3.54 (m, 1H, PC-CH_2_), 3.18–2.73 (m, 7H, PC-CH_2_). ^13^C NMR (100 MHz, DMSO-*d*_6_, ppm) δ = 178.6, 176.0 (naphthoquinone-CO), 174.6 (amide-CO), 166.6 (thiazole-C2), 139.4 (PC-C-6′), 139.3 (PC-C-11′), 139.1 (PC-C-14′), 138.8 (PC-C-3′), 135.6, 135.4 (Ar-C), 134.4, 134.2, 134.0, 133.6 (PC-CH), 132.5 (Ar-2CH), 132.3 (Ar-C), 131.8 (allyl-CH=), 131.3 (PC-C-4′), 131.2, 131.1, 126.7 (PC-CH), 126.5, 126.0 (Ar-CH), 116.4 (allyl-CH_2_=), 115.3 (Ar-C), 45.8 (allyl-CH_2_), 34.6 (PC-CH_2_-1′), 34.5 (PC-CH_2_-10′), 34.4 (PC-CH_2_-9′), 34.3 (PC-CH_2_-2′). IR (ATR, cm^−1^) *ṽ* = 3425–3165 (w, NH), 2921 (s, Ar-CH), 2847 (m, aliph-CH) 1672, 1650, 1589 (CO), 1564 (C=N). MS (FAB) *m*/*z* (%) = 520.2 [M + H]^+^ (60). HRMS (FAB; [M + H]^+^, C_31_H_26_O_3_N_3_^32^S_1_) Calc.: 520.1695, Found: 520.1693.

#### 3.1.6. (*Z*)-*N*-(2-(Ethylimino)-4,9-dioxo-4,9-dihydronaphtho[2,3-*d*]thiazol-3(2*H*)-yl)4′-[2.2]paracyclophanylamide (**3d**)

Red crystal (methanol), yield: 0.290 g (57%). ^1^H NMR (400 MHz, acetone-*d*_6_, ppm) δ = 10.17 (s, 1H, amide-NH), 8.10–7.84 (m, 4H, Ar-H), 7.16–6.85 (m, 1H, PC-H), 6.74–6.54 (m, 4H, PC-H), 6.52–6.39 (m, 2H, PC-H), 3.21–2.87 (m, 8H, PC-CH_2_), 1.58–1.16 (m, 2H, ethyl-CH_2_), 0.99 (t, *J* = 7.2 Hz, 3H, ethyl-CH_3_). ^13^C NMR (100 MHz, acetone-*d*_6_, ppm) δ = 179.9, 177.3 (naphthoquinone-CO), 175.9 (amide-CO), 141.1 (thiazole-C2), 140.9 (PC-C-6′), 140.8 (PC-C-11′), 140.2 (PC-C-14′), 140.1 (PC-C-3′), 136.6 (PC-CH), 135.4 (Ar-2C), 135.0, 134.8, 133.9 (PC-CH), 133.8 (Ar-2CH), 133.2 (Ar-C), 133.1, 132.9, 132.8 (PC-CH), 128.0 (PC-C-4′), 127.6, 127.2 (Ar-CH), 126.7 (Ar-C), 50.1 (ethyl-CH_2_), 36.0 (PC-CH_2_-1′), 35.9 (PC-CH_2_-10′), 35.8 (PC-CH_2_-9′), 35.7 (PC-CH_2_-2′), 14.2 (ethyl-CH_3_). IR (ATR, cm^−1^) *ṽ* = 3435–3150 (w, NH), 2925 (s, Ar-CH), 2847 (m, aliph-CH) 1667, 1647, 1589 (CO), 1562 (C=N). MS (FAB) *m*/*z* (%) = 508.2 [M + H]^+^ (50). HRMS (FAB; [M + H]^+^, C_30_H_26_O_3_N_3_^32^S_1_) Calc.: 508.1695, Found: 508.1693.

#### 3.1.7. (*Z*)-*N*-(2-(Cyclopropylimino)-4,9-dioxo-4,9-dihydronaphtho[2,3-*d*]thiazol-3(2*H*)-yl)-4′-[2.2]paracyclophanylamide (**3e**)

Red crystal (methanol), yield: 0.285 g (55%). ^1^H NMR (400 MHz, CDCl_3_-*d*, ppm) δ = 10.12 (s, 1H, amide-NH), 8.13–8.05 (m, 2H, Ar-H), 7.76–7.69 (m, 2H, Ar-H), 7.12–6.91 (m, 1H, PC-H), 6.76–6.43 (m, 6H, PC-H), 3.22–2.96 (m, 8H, PC-CH_2_), 2.58–2.54 (m, 1H, cyclopropyl-CH), 1.72–1.74 ppm (m, 1H, cyclopropyl-CH_2_), 1.20-0.47 (m, 3H, cyclopropyl-CH_2_). ^13^C NMR (100 MHz, CDCl_3_-*d*, ppm) δ = 179.5, 176.8 (naphthoquinone-CO), 173.6 (amide-CO), 167.8 (thiazole-C2), 141.1 (PC-C-6′), 140.3 (PC-C-11′), 139.3 (PC-C-14′), 136.4 (PC-C-3′), 135.8, 134.9 (Ar-C), 134.4 (PC-CH), 134.2 (Ar-C), 133.9, 133.8, 133.2, 132.7 (Ar-CH), 132.3 (PC-C-4′), 132.0, 131.6, 127.7, 127.3, 127.1, 126.6 (PC-CH), 126.3 (Ar-C), 35.6 (PC-CH_2_-1′), 35.4 (PC-CH_2_-10′), 35.2 (PC-CH_2_-9′), 34.9 (PC-CH_2_-2′), 26.0 (cyclopropyl-CH), 7.9 (cyclopropyl-CH_2_), 7.5 (cyclopropyl-CH_2_). IR (ATR, cm^−1^) *ṽ* = 3306 (w, NH), 2925 (s, Ar-CH), 2856 (m, aliph-CH) 1672, 1664, 1587 (CO), 1561 (C=N). MS (FAB) *m*/*z* (%) = 520.2 [M + H]^+^ (60). HRMS (FAB; [M + H]^+^, C_31_H_26_O_3_N_3_^32^S_1_) Calc.: 520.1695, Found: 520.1693.

#### 3.1.8. Reactions of Hydrazinecarbothioamide Derivatives **1a–f** with 2-bromo-2′-acetonaphthone (**4**); Preparation of Compounds **8a–d** and **9**.

A solution of **1a**–**e** (1.0 mmol, 1.00 equiv.) dissolved in 50 mL EtOAc was added to a solution of **4** (0.0249 g, 1.0 mmol, 1.00 equiv.) dissolved in 20-mL ethyl acetate. The resulting solution was stirred at room temperature for about 24–48 h (the reaction was monitored by thin-layer chromatography). The formed precipitate was filtered and washed with ethyl acetate several times (3 × 20 mL) to afford the target product. After removal of the solvent under reduced pressure, the crude was purified by flash chromatography using cyclohexane/ethyl acetate (10:8) as the eluent to afford compounds **8a**–**d** and **9**.

#### 3.1.9. 2-(2-(4′-[2.2]Paracyclophonyl)hydrazinyl)-4-(naphth-2-yl)-3-phenylthiazol-3-ium Bromide (**8a**)

Colorless crystal (ethanol), yield: 0.5 g (79%). ^1^H NMR (400 MHz, DMSO-*d*_6_, ppm) δ = 12.05 (s, 1H, amide-NH), 8.55 (s, 1H, Ar-H), 8.27–8.12 (m, 3H, Ar-H), 7.91–7.85 (m, 1H, Ar-H), 7.76–7.69 (m, 2H, Ar-H), 7.64–7.53 (m, 5H, Ph-H), 7.52 (s, 1H, thiazole-CH), 7.45 (s, 1H, NH), 6.64–6.57 (m, 1H, PC-H), 6.51–6.27 (m, 6H, PC-H), 3.53–3.42 (m, 1H, PC-CH_2_), 3.11–2.64 (m, 7H, PC-CH_2_). ^13^C NMR (100 MHz, DMSO-*d*_6_, ppm) δ = 165.6 (amide-CO), 142.0 (thiazole-C2), 141.4 (thiazole-C4), 139.3 (Ph-C), 139.0 (PC-C-6′), 138.4 (PC-C-11′), 138.3 (PC-C-14′), 138.2 (PC-C-3′), 136.5, 135.8 (Ar-C), 133.6, 132.7, 132.4 (PC-CH), 132.3 (Ar-C), 132.2, 131.5, 131.3 (Ar-CH), 130.4 (Ar-2CH), 130.3, 129.9 (Ar-CH), 129.4 (PC-C-4′), 128.6 128.5, 129.6, 128.9 (PC-CH), 128.3, 127.9, 127.3, 126.4, 124.5 (Ph-CH), 123.5 (thiazole-CH), 34.4 (PC-CH_2_-1′), 34.2 (PC-CH_2_-10′), 34.1 (PC-CH_2_-9′), 34.0 (PC-CH_2_-2′). IR (ATR, cm^−1^) *ṽ* = 3264–3058 (w, NH), 2925 (w, Ar-CH), 2857 (w, aliph-CH) 1694 (s, CO), 1560 (s, C=N). MS (ESI) *m*/*z* (%) = 552.2 [M]^+^ (100). HRMS (ESI, [M]^+^, [C_36_H_30_ON_3_^32^S_1_]^+^) Calc.: 552.2104, Found: 552.2085.

#### 3.1.10. 2-(2-(4′-[2.2]Paracyclophonyl)hydrazineyl)-3-benzyl-4-(naphth-2-yl)-thiazol-3-ium Bromide (**8b**)

Colorless crystal (ethanol), yield: 0.460 g (71%). ^1^H NMR (400 MHz, DMSO-*d*_6_, ppm) δ = 11.04 (s, 1H, amide-NH), 8.03–7.89 (m, 4H, Ar-H), 7.65–7.50 (m, 3H, Ar-H), 7.34–7.28 (m, 3H, Ph-H), 7.21 (s, 1H, thiazole-CH), 7.06 (d, *J* = 8.2 Hz, 2H, Ph-H), 6.92 (s, 1H, NH), 6.74 (dd, *J* = 7.8, 1.8 Hz, 1H, PC-H), 6.70–6.53 (m, 6H, PC-H), 5.38 (s, 2H, CH_2_Ph), 3.75–3.69 (m, 1H, PC-CH_2_), 3.21–2.93 (m, 7H, PC-CH_2_). ^13^C NMR (100 MHz, DMSO-*d*_6_, ppm) δ = 172.8 (amide-CO), 166.8 (thiazole-C2), 142.2 (thiazole-C4), 140.4 (PC-C-6′), 140.1 (PC-C-11′), 139.5 (PC-C-14′), 139.4 (PC-C-3′), 136.3 (Ph-C), 136.1, 134.5 (Ar-C), 133.3, 133.0, 132.9, 132.7 (PC-CH), 132.5 (Ar-C), 132.4 (PC-C-4′), 131.9, 131.6, 129.9, 129.0, 128.6, 128.5, 128.1 (Ar-CH), 127.9, 127.8, 127.3 (PC-CH), 126.8 (Ph-CH), 126.6 (Ph-2CH), 126.2, 126.1 (Ph-CH), 106.2 (thiazole-CH), 50.2 (CH_2_Ph), 35.1 (PC-CH_2_-1′), 34.9 (PC-CH_2_-10′), 34.7 (PC-CH_2_-9′), 34.6 (PC-CH_2_-2′). IR (ATR, cm^−1^) *ṽ* = 3254–3060 (w, NH), 2930 (w, Ar-CH), 2870 (w, aliph-CH) 1690 (s, CO), 1566 (s, C=N). MS (ESI) *m*/*z* (%) = 566.2 [M]^+^ (100). HRMS (ESI, [M]^+^, [C_37_H_32_ON_3_^32^S_1_]^+^) Calc.: 566.2261, Found: 566.2246.

#### 3.1.11. 3-Allyl-2-(2-(4′-[2.2]Paracyclophonyl)hydrazineyl)-4-(naphth-2-yl)-thiazol-3-ium Bromide (**8c**)

Colorless crystal (ethanol), yield: 0.430 g (72%). ^1^H NMR (400 MHz, DMSO-*d*_6_, ppm) δ = 11.23 (s, 1H, amide-NH), 8.20 (s, 1H, Ar-H), 8.12–8.02 (m, 3H, Ar-H), 7.70–7.63 (m, 3H, Ar-H), 7.35 (s, 1H, thiazole-CH), 6.95 (s, 1H, NH), 6.79–6.63 (m, 3H, PC-H), 6.62–6.51 (m, 4H, PC-H), 5.89–5.80 (m, 1H, allyl-CH=), 5.32–5.00 (m, 2H, allyl-CH_2_), 4.84–4.70 (m, 2H, allyl-CH_2_=), 3.73–3.67 (m, 1H, PC-CH_2_), 3.22–2.93 (m, 7H, PC-CH_2_). ^13^C NMR (100 MHz, DMSO-*d*_6_, ppm) δ = 173.8 (amide-CO), 168.2 (thiazole-C2), 143.4 (thiazole-C4), 141.5 (PC-C-6′), 141.1 (PC-C-11′), 140.4 (PC-C-14′), 140.3 (PC-C-3′), 137.3, 137.2 (Ar-C), 134.4, 133.9, 133.8, 133.6 (PC-CH), 133.6 (Ar-C), 133.5 (allyl-CH=), 133.4, 132.4, 132.2, 131.1, 130.9, 129.7, 129.5 (Ar-CH), 128.9 (PC-C-4′), 128.3, 127.2, 126.6 (PC-CH), 118.9 (allyl-CH_2_=), 108.3 (thiazole-CH), 50.4 (allyl-CH_2_), 36.0 (PC-CH_2_-1′), 35.8 (PC-CH_2_-10′), 35.6 (PC-CH_2_-9′), 35.5 (PC-CH_2_-2′). IR (ATR, cm^−1^) *ṽ* = 3255–3029 (w, NH), 3003–2866 (w, Ar-CH), 2819 (m, aliph-CH) 1683 (s, CO), 1581 (s, C=N). MS (ESI) *m*/*z* (%) = 516.2 [M]^+^ (100). HRMS (ESI, [M]^+^, [C_33_H_30_ON_3_^32^S_1_]^+^) Calc.: 516.2104, Found: 516.2091.

#### 3.1.12. 2-(2-(4′-[2.2]Paracyclophonyl)hydrazineyl)-3-ethyl-4-(naphth-2-yl)-thiazol-3-ium Bromide (**8d**)

Colorless crystal (ethanol), yield: 0.305 g (52%). ^1^H NMR (400 MHz, DMSO-*d*_6_, ppm) δ = 11.20 (s, 1H, amide-NH), 8.25 (s, 1H, Ar-H), 8.15–8.06 (m, 3H, Ar-H), 7.75–7.66 (m, 3H, Ar-H), 7.27 (s, 1H, thiazole-CH), 6.95 (s, 1H, NH), 6.77 (dd, *J* = 7.8, 1.8 Hz, 1H, PC-H), 6.66–6.53 (m, 6H, PC-H), 4.10 (q, *J* = 7.2 Hz, 2H, ethyl-CH_2_), 3.73–3.68 (m, 1H, PC-CH_2_), 3.22–2.94 (m, 7H, PC-CH_2_), 1.23 (t, *J* = 7.1 Hz, 3H, ethyl-CH_3_). ^13^C NMR (100 MHz, DMSO-*d*_6_, ppm) δ = 173.3 (amide-CO), 167.5 (thiazole-C2), 142.2 (thiazole-C4), 140.5 (PC-C-6′), 140.2 (PC-C-11′), 139.5 (PC-C-14′), 139.4 (PC-C-3′), 136.4, 136.3 (Ar-C), 133.5, 133.0, 132.9, 132.7 (PC-CH), 132.6 (Ar-C), 132.5, 131.5, 131.4, 130.3, 128.9, 128.6, 128.0 (Ar-CH), 127.9 (PC-C-4′), 127.4, 126.5, 125.9 (PC-CH), 107.3 (thiazole-CH), 43.1 (ethyl-CH_2_), 35.1 (PC-CH_2_-1′), 34.9 (PC-CH_2_-10′), 34.7 (PC-CH_2_-9′), 34.6 (PC-CH_2_-2′), 13.1 (ethyl-CH_3_). IR (ATR, cm^−1^) *ṽ* = 3264–3012 (w, NH), 3012 (w, Ar-CH), 2927–2744 (m, aliph-CH) 1683 (s, CO), 1584 (s, C=N). MS (ESI) *m*/*z* (%) = 504.2 [M]^+^ (100). HRMS (ESI, [M]^+^, [C_32_H_30_ON_3_^32^S_1_]^+^) Calc.: 504.2104, Found: 504.2088.

#### 3.1.13. 3-(4′-[2.2]Paracyclophan)amido-2-(cyclopropylamino)-4-(naphth-2-yl)thiazol-3-ium Bromide (**9**)

Colorless crystal (ethanol), yield: 0.300 g (60%). ^1^H NMR (400 MHz, DMSO-*d*_6_, ppm) δ = 11.79 (s, 1H, amide-NH), 8.45 (s, 1H, Ar-H), 8.28–8.01 (m, 3H, Ar-H), 7.89–7.61 (m, 3H, Ar-H), 7.47 (s, 1H, thiazole-CH), 6.92–6.60 (m, 3H, PC-H), 6.52–6.26 (m, 4H, PC-H), 5.36 (s, 1H, NH), 3.72–3.66 (m, 1H, PC-CH_2_), 3.19–2.78 (m, 7H, PC-CH_2_), 2.74–2.64 (m, 1H, cyclopropyl-CH), 1.04–0.95 (m, 2H, cyclopropyl-CH_2_), 0.81–0.75 (m, 2H, cyclopropyl-CH_2_). ^13^C NMR (100 MHz, DMSO-*d*_6_, ppm) δ = 172.0 (amide-CO), 166.1 (thiazole-C2), 142.4 (thiazole-C4), 142.3 (PC-C-6′), 139.7 (PC-C-11′), 139.5 (PC-C-14′), 138.8 (PC-C-3′), 137.1, 136.6 (Ar-C), 136.4, 134.1, 133.2, 132.9 (PC-CH), 132.7 (Ar-C), 131.8, 130.3, 129.9, 129.1, 128.8, 128.4, 127.9 (Ar-CH), 127.4 (PC-C-4′), 126.9, 126.3, 126.1 (PC-CH), 104.3 (thiazole-CH), 35.0 (PC-CH_2_-1′), 34.8 (PC-CH_2_-10′), 34.6 (PC-CH_2_-9′), 34.5 (PC-CH_2_-2′), 28.2 (cyclopropyl-CH), 7.3 (cyclopropyl-CH_2_), 7.2 (cyclopropyl-CH_2_). IR (ATR, cm^−1^) *ṽ* = 3260–3043 (w, NH), 3015–2886 (w, Ar-CH), 2819 (m, aliph-CH) 1679 (CO), 1588 (C=N). MS (ESI) *m*/*z* (%) = 516.2 [M]^+^ (65). HRMS (ESI, [M]^+^, [C_33_H_30_ON_3_^32^S_1_]^+^) Calc.: 516.2104, Found: 516.2155.

#### 3.1.14. Crystal Structure Determinations of **3e**, **8c**, and **9**

The single-crystal X-ray diffraction studies were carried out on a Bruker D8 Venture diffractometer with PhotonII detector at 123(2) K using Cu-Kα radiation (INCOATEC microfocus sealed tube, *λ* = 1.54178 Å, Middlesex County, MA, USA). Dual-space methods (SHELXT) [38] were used for structure solution, and refinement was carried out using SHELXL-2014 (full-matrix lowest-squares on *F^2^*) [39]. Hydrogen atoms were localized by difference electron density determination and refined using a riding model (H(N, O)-free). Semi-empirical absorption corrections were applied. For **3e**, an extinction correction was/were applied. The absolute structure of **8c** was determined by refinement of the Parsons x-parameter [41].

**3e**: red crystals, C_31_H_25_N_3_O_3_S·0.625(CH_4_O)·0.375(C_2_H_6_O), *M*_r_ = 556.90, crystal size 0.16 × 0.06 × 0.03 mm, monoclinic, space group *P*2_1_/c (No. 14), *a* = 7.2104(3) Å, *b* = 14.3984(6) Å, *c* = 26.4331(12) Å, *β* = 97.291(2)°, *V* = 2722.0(2) Å^3^, *Z* = 4, *ρ* = 1.359 Mg/m^−3^, *µ*(Cu-K_α_) = 1.428 mm^−1^, *F*(000) = 1172, *2θ*_max_ = 140-2°, 51,610 reflections, of which 5279 were independent (*R*_int_ = 0.039), 367 parameters, 43 restraints, *R*_1_ = 0.058 (for 5006 I > 2σ(I)), w*R*_2_ = 0.175 (all data), *S* = 1.10, largest diff. peak/hole = 0.40/−0.44 e Å^−3^. The structure was refined as a 2-component twin. There is a solvent disorder (MeOH vs. EtOH). In addition, the methylene moieties in C_2_H_4_-bridges were disordered (see the cif-file for details).

**8c**: colorless crystals, C_33_H_30_N_3_OS·Br, *M*_r_ = 596.57, crystal size 0.20 × 0.06 × 0.02 mm, orthorhombic, space group *P*na2_1_ (No. 33), *a* = 17.5892(5) Å, *b* = 25.1597(7) Å, *c* = 12.9433(4) Å, *V* = 5727.9(3) Å^3^, *Z* = 8, *ρ* = 1.384 Mg/m^−3^, *µ*(Cu-K_α_) = 2.87 mm^−1^, *F*(000) = 2464, *2θ*_max_ = 144.4°, 43,557 reflections, of which 10,648 were independent (*R*_int_ = 0.028), 698 parameters, 94 restraints, *R*_1_ = 0.033 (for 10,402 I > 2σ(I)), w*R*_2_ = 0.083 (all data), *S* = 1.07, largest diff. peak/hole = 0.64/−0.53 e Å^−3^, x = −0.017(6). One naphthalene moiety was disordered (see the cif-file for details).

**9**: colorless crystals, C_33_H_30_N_3_OS·Br, *M*_r_ = 596.57, crystal size 0.08 × 0.04 × 0.01 mm, monoclinic, space group *P*2_1_/c (No. 14), *a* = 16.9166(12) Å, *b* = 9.1979(6) Å, *c* = 18.6636(12) Å, *β* = 102.929(4)°, *V* = 2830.4(3) Å^3^, *Z* = 4, *ρ* = 1.400 Mg/m^−3^, *µ*(Cu-K_α_) = 2.92 mm^−1^, *F*(000) = 1232, *2θ*_max_ = 144.4°, 22,667 reflections, of which 5534 were independent (*R*_int_ = 0.152), 359 parameters, 2 restraints, *R*_1_ = 0.078 (for 2904 I > 2σ(I)), w*R*_2_ = 0.271 (all data), *S* = 1.01, largest diff. peak/hole = 0.59/−0.51 e Å^3^.

CCDC-1998918 (**3e**), 1998919 (**8c**), and 1998920 (**9**) contained the supplementary crystallographic data for this paper. These data can be obtained free of charge from The Cambridge Crystallographic Data Centre via www.ccdc.cam.ac.uk/data_request/cif.

### 3.2. Biological Evaluation

#### 3.2.1. Sixty Cancer Cell Lines Screening at the NCI

The NCI anticancer screening methodology has been described elsewhere in detail at https://dtp.cancer.gov/discovery_development/nci-60/methodology.htm. A summary of the experimental assay methodology is found in the Supplementary Data.

#### 3.2.2. Cytotoxic Activity Using the MTT Assay and Evaluation of IC_50_

MTT assay was performed to investigate the effect of the synthesized compounds on melanoma SK-MEL-5 to explore the antiproliferative potential of the compounds. MTT assay was performed according to the reported data [42] (see Supplementary Data).

#### 3.2.3. CDK Inhibitory Assay

A cell-free assay was used to explore the mechanism of inhibition of the CDK kinase of the most active compound according to the reported method (see Supplementary Data).

#### 3.2.4. Cell Cycle and Annexin-V FITC Apoptotic Study

Generally, the Annexin V-FITC/PI (fluorescein-isothiocyanate/propidium iodide) Apoptosis Detection Kit (ab#139418) (BioVision Research Products, 980 Linda Vista Avenue, MountainView, CA, 94043, USA) was used for the apoptosis assay according to the manufacturer’s instructions. Firstly, NCI-H522 cells (4 × 106 cells/well) were incubated with compound **3c** (at conc. IC_50_ = 0.81 μM) for 24 h. A control experiment (with untreated NCI-H522 cells) was carried out for comparison. Cells were washed three times with ice-cold PBS, resuspended in PBS, and then stained for 15 min with 5-mL annexin V-FITC and 5-mL PI binding buffer in a dark place at ambient temperature. Finally, the BD FACS CALIBER flow cytometer was used for the analysis of stained cells and the measurement of the extent of apoptosis [43].

#### 3.2.5. Inhibition of Phospho-CDK1/CDC2 Cell-Based Phosphorylation in SK-MEL-5 Cancer Cells

The Colorimetric Cell-Based ELISA Kit (Durham, NC, USA) allowed for the qualitative determination of the target protein concentration achieved by an indirect ELISA format [44] (see Supplementary Data).

#### 3.2.6. Caspase-3 Activation Assay

Cell line cells of SK-MEL-5 were obtained from ATCC Camarillo, CA 93012, USA. RPMI 1640 containing 10% FBS was used to allow cells to grow at 37 °C and stimulated with the compounds to be tested for caspase-3 [45] (see Supplementary Data).

#### 3.2.7. Docking Study

A molecular docking study was performed for the most active synthesized compounds with CDK1. The docking of compounds **3a**–**e**, **8a**–**d**, **9**, and Dinaciclib was carried out using AutoDock4.2.6 software [46]. Target compounds were constructed, and their 3D structures were generated using Omega2 software [47,48]. Energy minimization was performed for the generated conformations by the MMFF94S force field using SZYBKI [49]. The partial charges were calculated using the Gasteiger method [50] X-ray. The crystallographic structure of the CDK1 in complex with Dinaciclib (PDB code: 6GU6 [51]) was downloaded from the Protein Data Bank (www.rcsb.org) and prepared for docking calculations by deleting the heteroatoms and adding hydrogen atoms with the help of the H++ server [52]. The AutoDock protocol was followed to prepare the pdbqt file for the receptor [53]. The receptor was fixed, and docking of the designed compounds was done into the catalytic site of the CDK1 enzyme. The maximum number of energy evaluations (eval) was set to 25,000,000, and the number of genetic algorithm (GA) runs was set to 250. The grid with a size of 60 Å × 60 Å × 60 Å was positioned at the center of the active site. The predicted binding poses for each compound were processed by the built-in clustering analysis (1.0 Å RMSD tolerance), with the conformation of the lowest energy with respect to the largest cluster selected as the representative.

#### 3.2.8. Statistical Analysis

The experimental results were quantified by GraphPad Prism 5 (Version 5.0, GraphPad Software, La Jolla, CA, USA). Comparisons between treatments were analyzed by a one-way ANOVA (and nonparametric) tests. *p*-values were labeled in the figures, and standard error of the mean was also shown.

## 4. Conclusions

In the current study, a novel three-series assembly of three bioactive entities: 1,4-dihydronaphthoquinone, thiazole, and [2.2]paracyclophane derivative in solid hybrid structures were synthesized and characterized. One-dose anticancer test results indicated that compounds **3a**–**e** exhibited the highest ability to inhibit the proliferation of different cancer cell lines. An in vitro five-dose full NCI 60-cell panel assay revealed that compounds **3c**–**e** exhibited a broad-spectrum antitumor activity against the nine tumor subpanels tested without pronounced selectivity. Compounds **3a**–**e** exhibited the potent inhibition of melanoma SK-MEL-5 cancer cell growth compared to Dinaciclib as a reference. Compound **3c** showed the lowest IC_50_ of 54.8 nM on the target enzyme CDK1 in comparison to Dinaciclib as a reference. Accordingly, compound **3c** was extensively investigated and showed a marked downregulation of phospho-Tyr15 with a level (7.45 pg/mL) comparable to the reference. Furthermore, the effect of compound **3c** on caspase-3 was evaluated and found to increase in its level by 8.66-fold. The effect of compound **3c** on the cell cycle arrest was also examined. Moreover, a molecular docking study was performed to explain the binding mode and affinity of the synthesized compounds. These results led to the discovery of promising novel hybrids of interesting thiazole/paracyclophanes as a starting point in the medicinal chemistry art that warrant further research and development as potential cancer candidates.

## Figures and Tables

**Table 1:** Growth percent of compounds **3a**–**e** and **8a**–**d** (at conc. 10^−5^ M) against different cell lines.

Panel/Cell Line		3a	3b	3c	3d	3e	8a	8b	8c	8d
Leukemia	CCRF-CEM	20.15	59.23	103.65	116.03	77.39	7.77	49.02	58.83	48.46
	HL-60(TB)	8.41	72.97	120.12	118.34	97.61	3.67	17.11	50.76	46.34
	K-562	22.24	51.17	112.34	128.49	74.12	14.31	54.53	65.03	58.59
	MOLT-4	16.68	78.95	115.46	132.80	91.47	11.35	32.30	54.75	43.07
	RPMI-8226	24.80	41.03	127.65	133.56	80.48	9.83	37.81	74.17	62.22
	SR	38.87	51.66	108.54	103.66	70.59	11.99	53.27	60.88	45.35
Non-Small Cell Lung Cancer	A549/ATCC	35.96	20.00	76.74	138.85	32.30	2.64	12.65	43.90	28.88
	EKVX	29.59	11.57	120.17	161.32	32.49	7.32	21.69	41.82	31.03
	HOP-62	33.63	22.46	80.49	158.60	45.90	6.03	22.55	24.84	19.99
	HOP-92	0	0	78.49	109.79	4.51	3.02	11.28	40.36	28.25
	NCI-H226	48.81	33.73	51.50	26.96	35.15	5.88	13.23	24.77	21.36
	NCI-H23	69.39	68.04	134.07	132.12	90.37	2.21	21.82	44.16	39.27
	NCI-H322M	0.21	0	32.21	19.52	3.95	1.65	5.69	2.44	4.81
	NCI-H460	28.14	25.07	87.28	90.38	46.08	0	39.19	47.70	29.96
	NCI-H522	31.97	47.13	120.20	134.81	62.67	7.64	18.21	23.62	22.73
Colon Cancer	COLO 205	0	0	80.85	57.45	3.67	0	15.44	55.51	39.55
	HCC-2998	13.21	0	133.50	137.87	38.63	0	2.86	10.89	0
	HCT-116	39.13	44.14	132.42	150.92	109.10	8.46	63.36	72.90	59.89
	HCT-15	48.69	44.66	168.35	168.11	97.21	0	57.16	50.52	33.91
	HT29	8.23	0.35	73.95	50.97	0	5.99	26.49	69.86	50.14
	KM12	21.19	25.15	93.96	129.88	64.80	0	18.15	43.43	17.79
	SW-620	24.10	22.49	159.75	156.56	81.66	4.29	20.05	27.07	18.93
CNS Cancer	SF-268	32.26	41.37	91.21	110.65	54.82	15.04	19.26	37.67	29.00
	SF-295	12.21	5.24	30.80	69.03	20.44	9.26	14.92	54.00	36.89
	SF-539	7.45	12.52	198.29	194.41	94.83	0	4.73	22.07	18.69
	SNB-19	39.25	25.88	95.41	174.30	66.30	1.20	9.35	33.22	17.91
	SNB-75	63.66	66.13	194.63	198.23	110.40	20.65	27.60	46.73	41.79
	U251	17.35	29.29	99.78	181.45	67.40	8.65	42.13	46.10	26.06
Melanoma	LOX IMVI	37.84	64.25	180.28	183.86	99.49	4.55	39.20	40.06	22.40
	MALME-3M	169.26	94.48	188.59	187.38	164.33	0	0.23	23.30	14.64
	M14	95.86	80.70	193.88	193.47	121.83	0	13.94	24.45	14.96
	MDA-MB-435	172.49	185.53	193.49	197.05	193.24	0	7.06	32.55	24.57
	SK-MEL-2	24.77	22.73	117.29	140.56	50.50	0	0.55	14.41	21.90
	SK-MEL-28	7.59	8.64	135.36	181.24	59.83	0	8.15	27.78	18.01
	SK-MEL-5	60.33	48.69	198.49	196.93	177.60	3.12	44.92	110.14	83.18
	UACC-257	37.58	47.42	172.55	190.50	128.61	0	5.58	51.31	33.52
	UACC-62	29.18	33.59	136.03	154.04	112.39	5.12	34.09	47.31	46.24
Ovarian Cancer	IGROV1	51.23	46.96	120.49	139.81	65.42	0	12.89	18.70	19.70
	OVCAR-3	35.24	48.92	126.39	118.83	98.89	3.35	30.37	46.79	39.78
	OVCAR-4	30.63	52.34	196.31	198.41	59.51	10.84	29.10	66.51	59.08
	OVCAR-5	0	0	165.70	177.37	0	0	0	0	0
	OVCAR-8	37.77	53.68	94.40	182.60	96.21	2.18	11.63	34.51	22.47
	NCI/ADR-RES	29.79	35.70	112.75	122.75	82.21	0.82	8.21	32.84	28.33
	SK-OV-3	23.68	27.13	44.32	38.03	40.91	4.34	19.80	17.91	21.63
Renal Cancer	786-0	19.04	17.33	121.83	190.37	31.40	0.60	12.69	29.94	22.26
	A498	6.32	13.32	44.03	48.42	10.55	26.71	13.08	37.26	27.25
	ACHN	57.35	57.98	195.25	190.19	74.36	0	11.65	27.66	15.91
	CAKI-1	67.87	56.64	79.67	91.15	69.16	9.75	42.98	39.11	32.21
	RXF 393	29.32	39.72	126.32	187.24	46.32	0	13.75	25.92	26.73
	SN12C	29.81	38.18	196.64	191.17	69.12	7.24	19.77	25.74	18.58
	TK-10	−8.43	0	41.72	11.39	0	0	8.81	20.32	20.52
	UO-31	66.37	61.82	135.24	173.08	78.47	32.71	42.83	57.68	49.11
Prostate Cancer	PC-3	33.67	42.21	99.63	102.79	69.33	14.03	54.49	62.23	51.20
	DU-145	28.33	11.20	190.66	191.42	66.79	0	7.12	25.02	12.24
Breast Cancer	MCF7	54.39	59.97	162.80	163.07	88.50	16.30	35.02	64.10	47.06
	MDA-MB-231/ATCC	60.00	59.61	106.76	107.49	84.61	0	10.63	7.06	4.91
	HS 578T	21.13	27.78	109.43	108.98	53.83	15.85	21.79	41.31	38.71
	BT-549	32.10	22.75	199.09	197.40	62.82	7.56	17.13	46.06	39.07
	T-47D	28.91	64.88	103.21	119.84	79.61	11.05	46.29	79.21	75.60
	MDA-MB-468	0	20.05	130.08	133.70	67.59	0	32.25	60.86	56.41

**Table 2:** Results of in vitro five-doses testing of nine human cancer types and selectivity for compounds **3c, 3d,** and **3e**.

Panel	Cell Line	3c					3d					3e				
		GI50			TGI	LC50	GI50			TGI	LC50	GI50			TGI	LC50
		Conc. Per Cell Line	Subpanel MID ^b^	Selectivity Ratio (MID ^a^: MID ^b^)			Conc. Per Cell Line	Subpanel MID ^b^	Selectivity Ratio (MID ^a^: MID ^b^)			Conc. Per Cell Line	Subpanel MID ^b^	Selectivity Ratio (MID ^a^: MID ^b^)		
leukemia	**CCRF-CEM**	**2.39**	**2.21**	**0.99**	**>100**	**>100**	9.98	7.87	0.73	3.33	>3.33	2.53	2.14	1.16	>100	>100
	HL-60(TB)	3.95			3.26	>100	7.70			2.05	>3.33	2.18			6.26	>100
	K-562	1.69			--	>100	8.79			3.33	>3.33	2.43			>100	>100
	MOLT-4	1.22			5.76	>100	5.99			1.72	>3.33	2.22			8.89	>100
	RPMI-8226	2.21			--	>100	7.17			2.01	>3.33	1.86			6.47	>100
	SR	1.78			>100	>100	7.57			2.95	>3.33	1.64			--	>100
Non-Small Cell Lung Cancer	A549/ATCC	2.26	1.69	1.03	5.61	>100	8.35	5.07	1.14	2.07	>3.33	2.42	2.31	1.07	6.07	>100
	EKVX	1.39			3.46	8.60	5.01			1.15	2.64	1.87			1.25	
	HOP-62	1.96			4.85	1.65	1.85			6.48	1.53	4.28			1.72	
	HOP-92	1.56			4.19	>100	7.41			2.60	1.56	2.26			1.44	
	NCI-H226	1.61			3.35	6.99	9.36			4.09	1.37	1.81			5.83	
	NCI-H23	1.46			3.76	9.72	5.50			1.50	>3.33	1.31			4.32	>100
	NCI-H322M	1.55			2.92	5.50	1.61			5.90	1.43	1.93			5.13	
	NCI-H460	1.94			3.97	8.11	1.06			3.60	1.50	3.35			1.22	
	NCI-H522	1.49			4.19	>100	5.51			1.37	4.64	1.60			4.69	>100
Colon Cancer	COLO 205	1.94	2.61	0.84	4.24	9.28	3.63	5.99	0.96	9.06	2.26	3.36	2.50	0.99	1.27	6.62
	HCC-2998	1.62			3.14	6.06	9.48			2.91	1.06	2.34			6.67	2.40
	HCT-116	5.77			2.58	9.31	6.67			1.70	6.89	1.94			6.45	2.73
	HCT-15	1.52			3.50	8.07	5.49			1.18	2.54	1.51			3.24	6.96
	HT29	3.09			>100	>100	1.15			3.18	>3.33	3.32			2.87	>100
	KM12	2.68			8.80	9.05	9.99			3.56	>3.33	3.12			1.37	8.18
	SW-620	1.63			3.45	7.30	5.51			1.20	2.62	1.92			4.32	9.71
CNS Cancer	SF-268	1.68	2.79	0.78	4.48	>100	6.15	5.12	1.13	1.65	>3.33	2.54	2.43	1.02	1.10	5.91
	SF-295	1.68			3.08	5.66	6.15			1.27	2.60	3.55			1.27	3.57
	SF-539	1.69			3.12	5.79	5.54			1.02	1.89	1.67			3.06	5.58
	SNB-19	1.67			3.44	7.05	6.45			1.64	5.31	3.09			1.17	3.42
	SNB-75	8.43			2.76	7.79	1.32			7.46	1.74	1.32			6.04	2.51
	U251	1.60			4.28	>100	5.12			1.12	2.43	2.41			1.11	>100
Melanoma	LOX IMVI	5.32	2.32	0.95	2.10	5.86	4.06	5.77	1.00	9.04	2.01	9.66	3.12	0.79	2.62	6.98
	MALME-3M	3.83			1.58	4.00	4.42			8.70	1.71	5.70			1.75	4.19
	M14	1.63			3.30	6.65	5.54			1.06	2.01	1.33			2.83	6.03
	MDA-MB-435	1.94			3.63	6.82	5.98			1.09	1.97	1.76			3.23	5.93
	SK-MEL-2	1.74			4.32	7.27	7.39			1.58	9.60	2.47			7.25	9.29
	SK-MEL-28	1.87			3.35	6.00	6.87			1.21	2.15	2.40			5.22	1.39
	SK-MEL-5	1.41			2.75	5.37	5.51			1.02	1.89	1.43			2.76	5.32
	UACC-257	1.55			3.15	6.39	5.93			1.14	2.20	1.69			3.41	6.88
	UACC-62	1.63			3.91	9.38	6.23			1.33	2.84	1.62			3.52	7.66
Ovarian Cancer	IGROV1	1.13	2.25	0.98	3.21	9.08	4.46	5.38	1.07	1.31	5.78	1.16	2.53	0.98	4.74	2.58
	OVCAR-3	3.28			1.43	5.03	5.22			1.28	3.16	1.53			4.42	1.59
	OVCAR-4	1.26			2.56	5.18	4.54			8.87	1.73	1.61			4.58	1.59
	OVCAR-5	2.03			4.37	9.42	5.86			1.15	2.26	1.83			3.53	6.79
	OVCAR-8	2.13			1.12	>100	5.81			1.24	2.63	2.62			1.16	4.10
	NCI/ADR-RES	2.24			--	>100	7.43			2.17	>3.33	1.93			6.77	>100
	SK-OV-3	3.70			2.34	>100	4.35			8.75	1.76	7.03			2.14	5.23
Renal Cancer	786-0	2.30	2.25	0.98	5.70	>100	7.48	4.82	1.20	1.70	>3.33	2.75	2.53	0.98	7.93	9.61
	A498	1.05			4.70	>100	6.93			2.90	>3.33	2.07			6.34	>100
	ACHN	1.42			2.91	5.96	4.92			9.65	1.89	1.39			3.03	6.60
	CAKI-1	1.18			2.86	6.92	4.80			2.14	8.98	2.48			1.49	3.87
	RXF393	1.35			2.71	5.43	5.02			9.86	1.94	1.85			4.75	1.50
	SN12C	1.22			2.79	6.42	5.06			1.07	2.24	1.33			2.61	5.11
	TK-10	4.48			8.14	4.13	1.03			1.71	2.85	1.84			3.47	6.54
	UO-31	4.96			2.09	4.60	3.34			8.60	2.21	8.50			2.77	7.85
Prostate Cancer	PC-3	1.49	1.67	1.32	8.81	>100	6.20	6.29	0.92	2.69	>3.33	1.62	1.74	1.43	1.07	>100
	DU-145	1.85			3.45	6.46	6.37			1.19	2.22	1.85			3.43	6.36
Breast Cancer	MCF7	1.23	1.73	1.27	3.36	9.17	5.83	6.67	0.87	1.28	2.82	1.42	1.90	1.31	3.29	7.62
	MDA-MB-231/ATCC	1.27			6.69	>100	6.52			2.69	>3.33	1.37			9.82	>100
	HS 578T	2.04			6.93	>100	7.82			2.05	>3.33	2.09			6.03	>100
	BT-549	1.53			2.91	5.54	5.22			9.83	1.85	1.50			2.91	5.64
	T-47D	2.03			7.29	>100	6.98			2.30	>3.33	2.49			1.44	>100
	MDA-MB-468	2.28			5.72	>100	7.66			2.04	>3.33	2.55			7.32	>100
MID ^a^	2.20						5.77					2.48				

MID ^a^ = Average sensitivity of all cell lines in mM and MID ^b^ = average sensitivity of all cell lines of a particular subpanel in µM.

**Table 3:** Antiproliferative activity of the target compounds **3b, 3c, 3e, 8a–d**, **9**, Dinaciclib, and the control.

Compound	Cytotoxicity IC_50_ (µM) ^a^ ± SEM
**3b**	9.11 ± 0.39 ***
**3c**	0.81 ± 0.03 ***
**3e**	4.18 ± 0.18 ***
**8a**	26.5 ± 1.13 **
**8b**	21.5 ± 0.92 **
**8c**	30.8 ± 1.32 **
**8d**	87.3 ± 3.73 *
**9**	12.1 ± 0.52 ***
**Dinaciclib**	5.97 ± 0.25 ***
**Control**	0

^a^ IC_50_ = compound concentration required to inhibit tumor cell proliferation by 50%. All data were obtained by triplet testing data are expressed as the mean ± SEM from the dose-response curves of at lowest three independent experiments. Results were significantly different from the control at *** *p* < 0.05. Results indicating at *, ** means less significant.

**Table 4:** Antiproliferative IC_50_ ± SEM (µM) activity of compounds **3c**, **3e**, and Dinaciclib.

Compound	Cytotoxicity IC_50_ ± SEM (µM) WI38
	32.59 ± 1.44
**3e**	39.86 ± 1.76
**Dinaciclib**	22.01 ± 0.97

The data given are mean values derived from at lowest three replicates ± SEM.

**Table 5:** Caspase-3 conc. (pg/mL) ± SEM and fold change levels for **3c** and Dinaciclib on the SK-MEL-5 cell line.

Compound	Caspase 3	
	Conc. pg/mL	Fold Change
**3c**	519.4 ± 5.8 ***	8.66
**Dinaciclib**	476.7 ± 8.4 ***	7.95
**Control**	59.95 ± 2.1	1

Results Significantly different from control at *** *p* < 0.05.

## Supplementary Materials

The supplementary data for this article can be found online. **1. Chemistry**, Figure S1. ^1^H NMR spectrum of compound **3a**. Figure S2. ^13^C NMR spectrum of compound **3a**. Figure S3. Mass spectrum of compound **3a**. Figure S4. HRMS spectrum of compound **3a**. Figure S5. ^1^H NMR spectrum of compound **3b**. Figure S6. ^13^C NMR spectrum of compound **3b**. Figure S7. Mass spectrum of compound **3b**. Figure S8. HRMS spectrum of compound **3b**. Figure S9. ^1^H NMR spectrum of compound **3c**. Figure S10. ^13^C NMR spectrum of compound **3c**. Figure S11. Mass spectrum of compound **3c**. Figure S12. HRMS spectrum of compound **3c**. Figure S13. ^1^H NMR spectrum of compound **3d**. Figure S14. ^13^C NMR spectrum of compound **3d**. Figure S15. Mass spectrum of compound **3d**. Figure S16. HRMS spectrum of compound **3d**. Figure S17. ^1^H NMR spectrum of compound **3e**. Figure S18. ^13^C NMR spectrum of compound **3e**. Figure S19. Mass spectrum of compound **3e**. Figure S20. HRMS spectrum of compound **3e**. Figure S21. ^1^H NMR spectrum of compound **8a**. Figure S22. ^13^C NMR spectrum of compound **8a**. Figure S23. HRMS and Mass spectrum of compound **8a**. Figure S24. ^1^H NMR spectrum of compound **8b**. Figure S25. ^13^C NMR spectrum of compound **8b**. Figure S26. HRMS and Mass spectrum of compound **8b**. Figure S27. ^1^H NMR spectrum of compound **8c**. Figure S28. ^13^C NMR spectrum of compound **8c**. Figure S29. HRMS and Mass spectrum of compound **8c**. Figure S30. ^1^H NMR spectrum of compound **8d**. Figure S31. ^13^C NMR spectrum of compound **8d**. Figure S32. HRMS and Mass spectrum of compound **8d**. Figure S33. 1H NMR spectrum of compound **9**. Figure S34. ^13^C NMR spectrum of compound **9**. Figure S35. HRMS and Mass spectrum of compound **8a**. 2. Biology Table S1. DNA content % using propidium iodide flow cytometry. Results Significantly different from control at *** *p* < 0.05. Table S2. Predicted binding scores for compounds **3a-e**, **8a-d**, **9** and Dinacicilib in CDK1 active site (PDB code: 6GU6). Table S3. Predicted binding scores for *p*-xylene analogs of compounds **3a-e** in CDK1 active site (PDB code: 6GU6). Figure S36. Dose Response Curves for all cell line for compound **3d**. Figure S37. Log 10 concentration of compound **10d**. Figure S38. Log 10 concentration of compound **3d**. Figure S39. Dose Response Curves for all cell line for compound **3d**. Figure S40. Log 10 concentration of compound **3e**. Figure S41. Log 10 concentration of compound **3e**. Figure S42. Dose Response Curves for all cell line for compound **3c**. Figure S43. Log 10 concentration of compound **3c**. Figure S44. Log 10 concentration of compound **3c**.
