# Supplementary material for: Design, Synthesis, and Molecular Docking of Paracyclophanyl-Thiazole Hybrids as Novel CDK1 Inhibitors and Apoptosis Inducing Anti-Melanoma Agents
Source: Molecules. 2020 Nov 27;25(23):5569. doi: 10.3390/molecules25235569 (PMC7729638; doi:10.3390/molecules25235569)
Supplement: Supplementary file 1 [file molecules-25-05569-s001.pdf]

# Design, Synthesis and Molecular Docking of Paracyclophanyl-Thiazole Hybrids as Novel CDK1 Inhibitors & Apoptosis-Inducing Anti-Melanoma Agents

**Ashraf A Aly,[1*] Stefan Bräse,[2,3*] Alaa A. Hassan,[1] Nasr K. Mohamed,[1] Lamiaa E. Abd El-Haleem,[2,3] Martin Nieger,[4] Nesrin M. Morsy,[5] Mohammed B Alshammari[6] , Mahmoud A. A. Ibrahim [1], and Elshimaa M. N. Abdelhafez[7]***

**Figure S1.** $^{1}$H NMR spectrum of compound **3a**

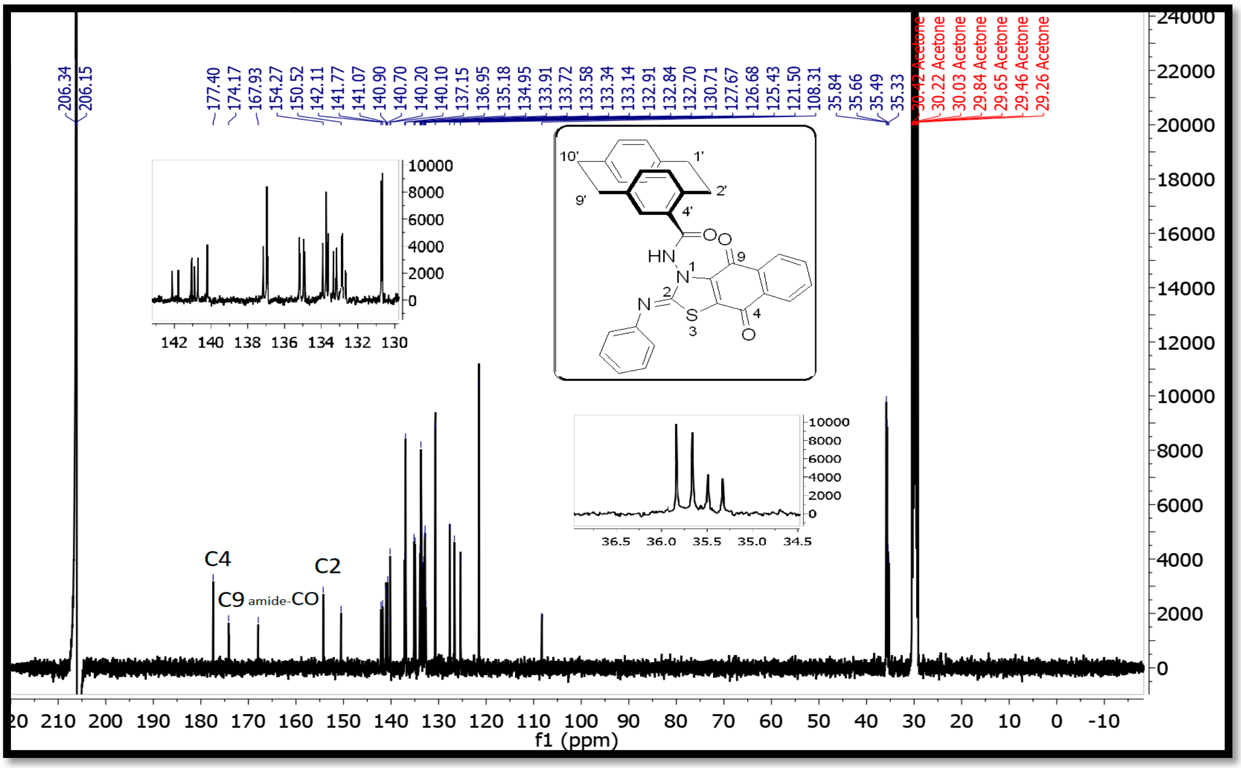


**Figure S2.** $^{13}C$ NMR spectrum of compound **3a**

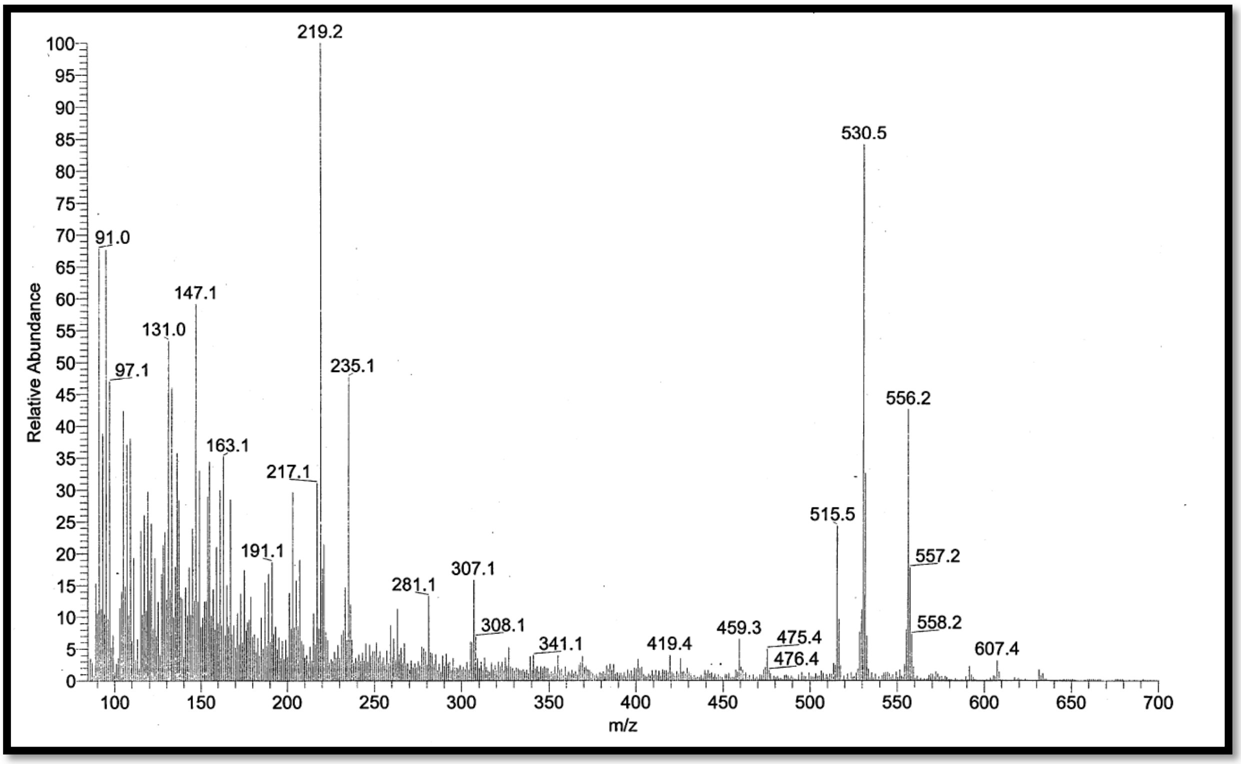


**Figure S3.** Mass spectrum of compound **3a**

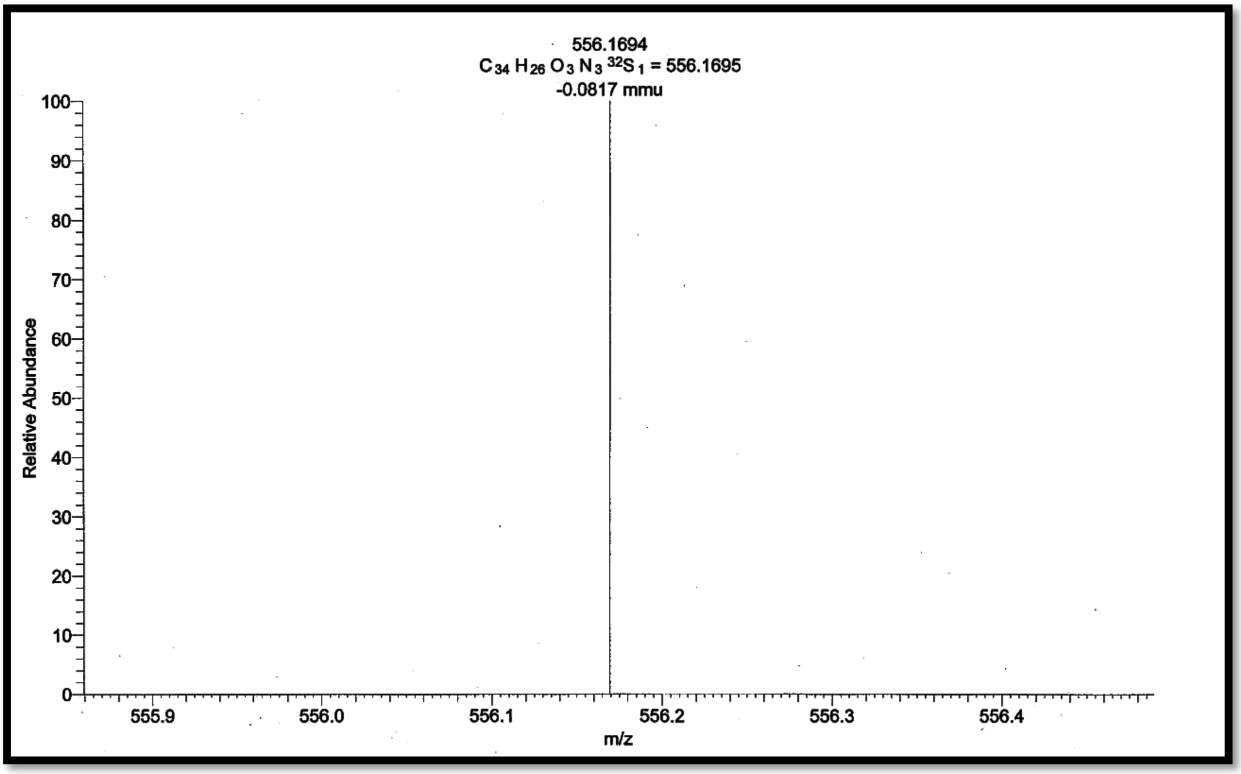


**Figure S4.** HRMS spectrum of compound **3a**

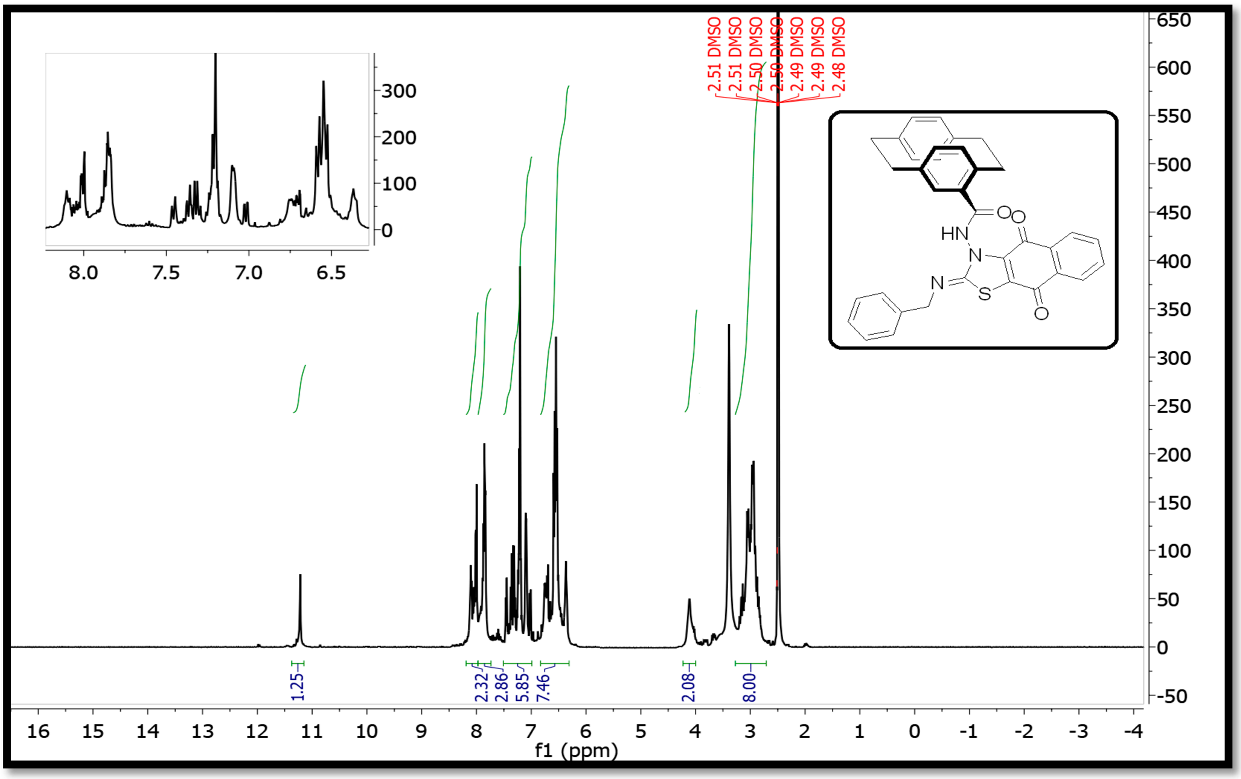


**Figure S5.** $^{1}$H NMR spectrum of compound **3b**

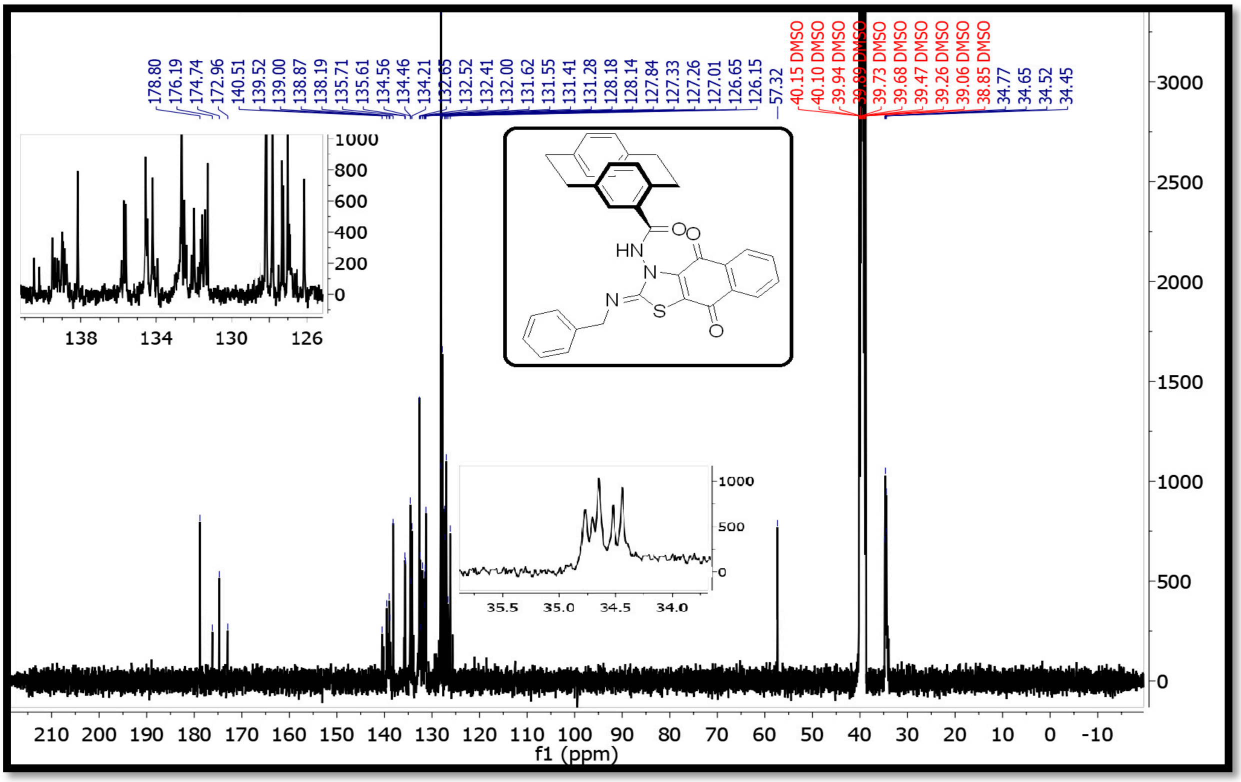


**Figure S6.** $^{13}C$ NMR spectrum of compound **3b**

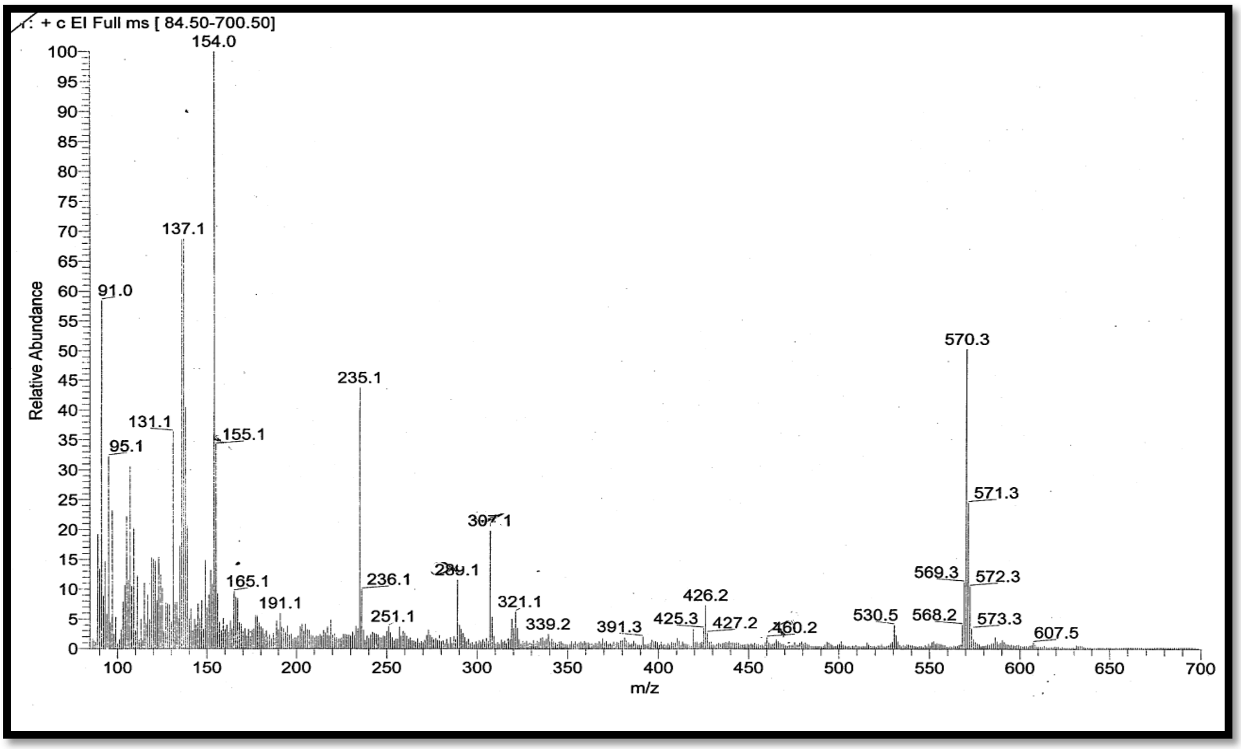


**Figure S7.** Mass spectrum of compound **3b**

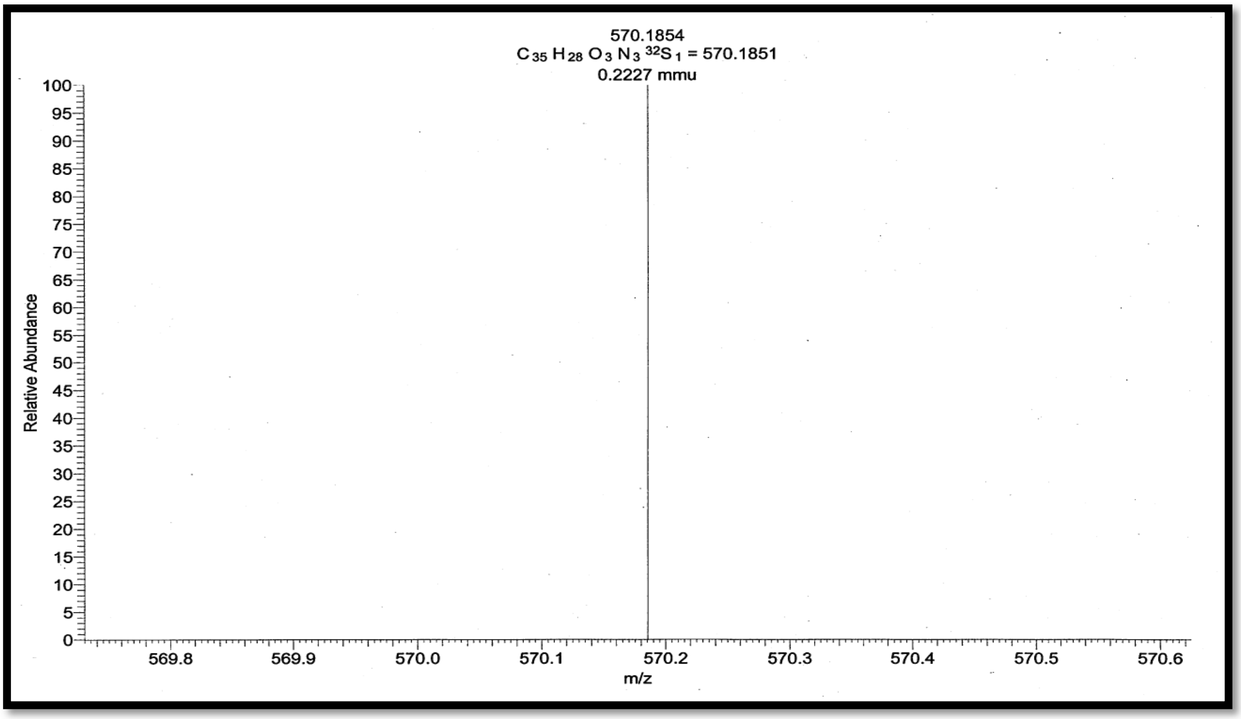


**Figure S8.** HRMS spectrum of compound **3b**

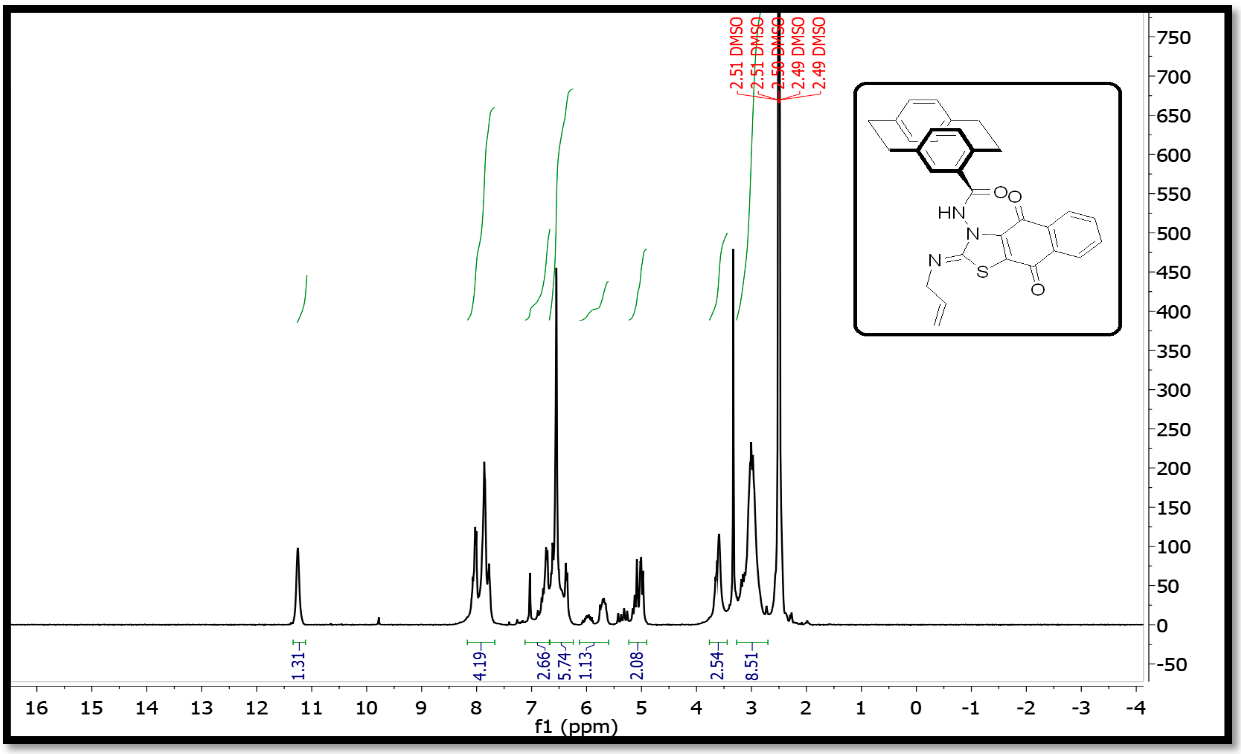


**Figure S9.** $^{1}$H NMR spectrum of compound **3c**

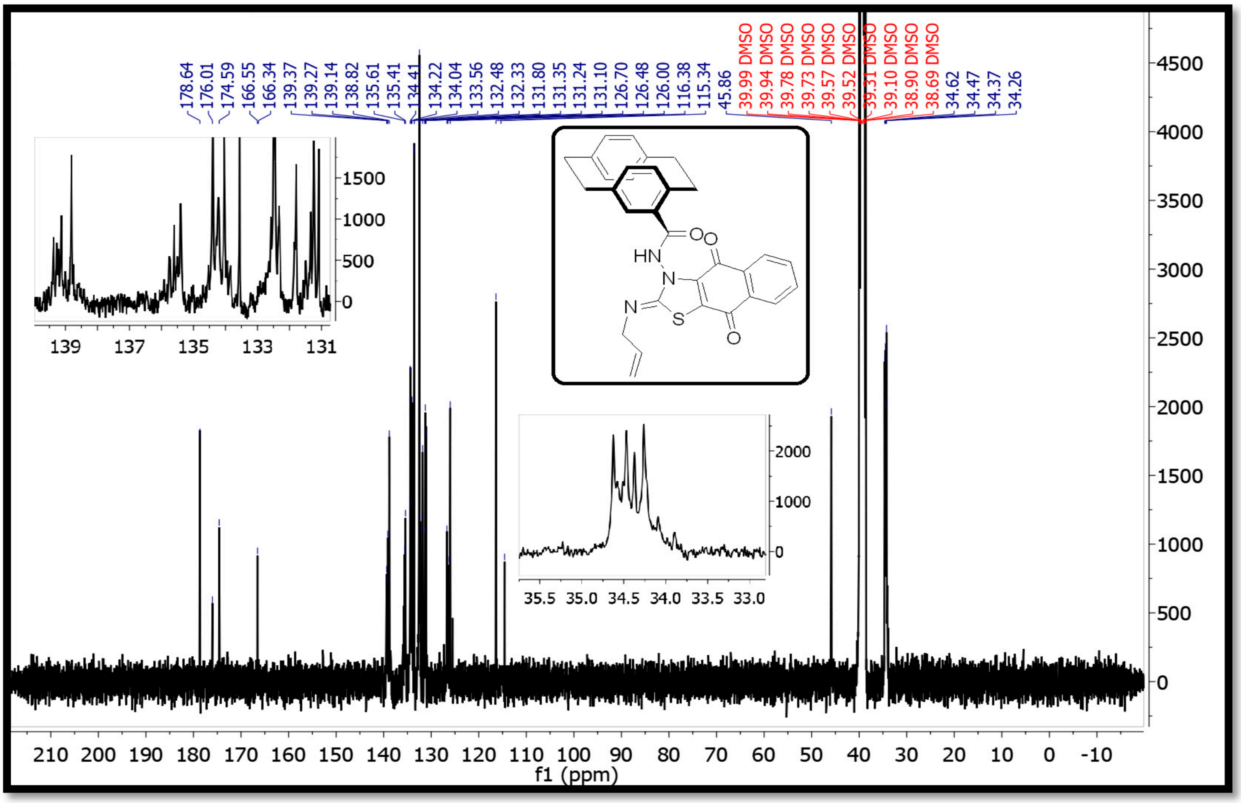

**Figure S10.** $^{13}$C NMR spectrum of compound **3c**

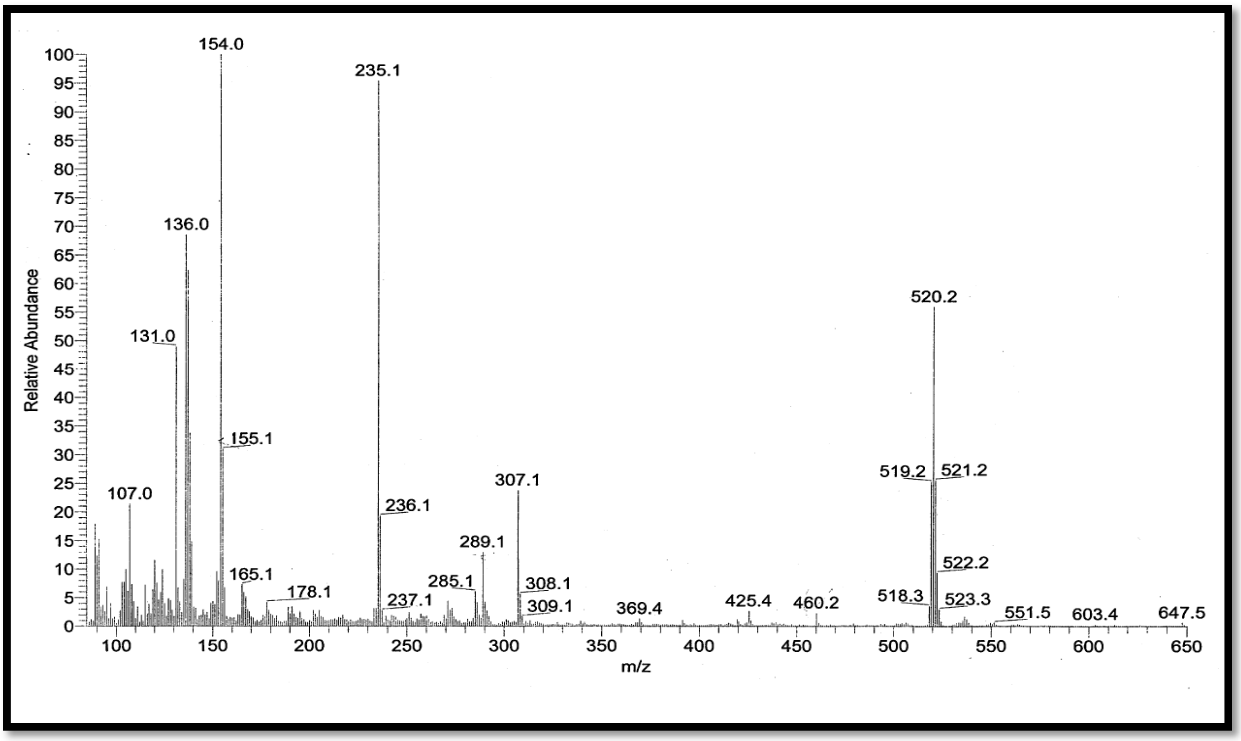

**Figure S11.** Mass spectrum of compound **3c**

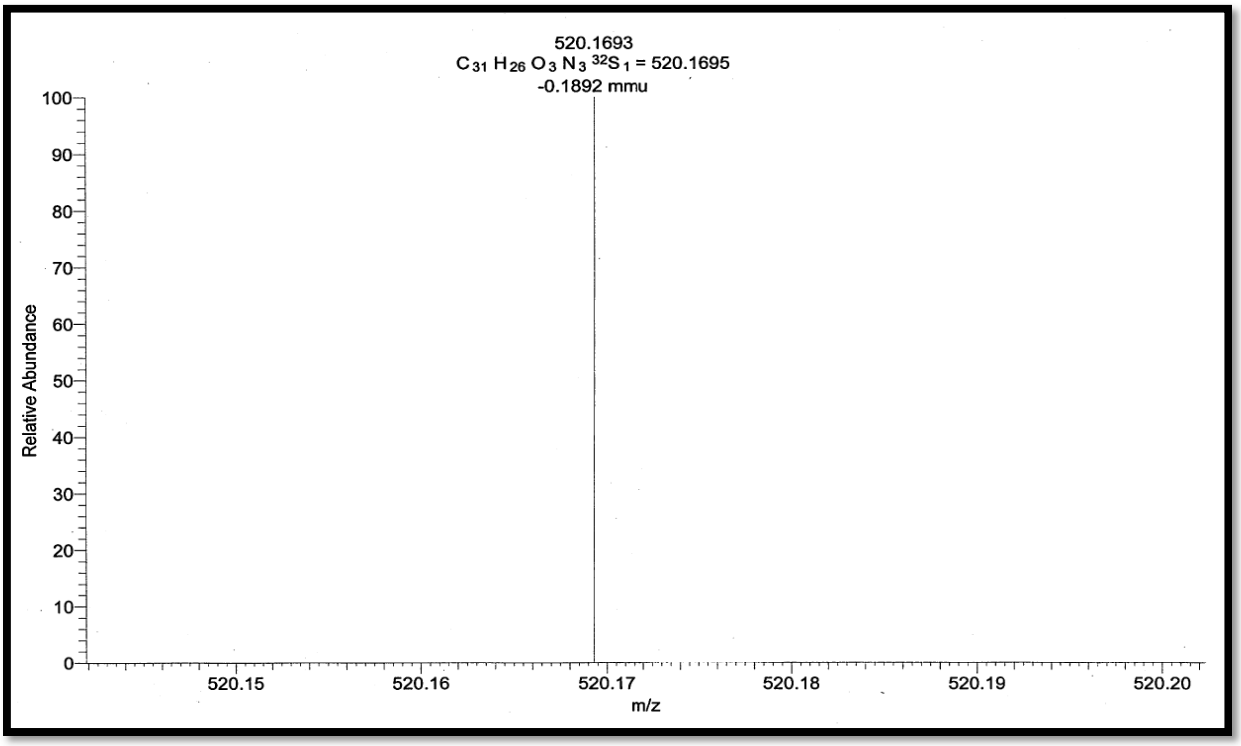


**Figure S12.** HRMS spectrum of compound **3c**

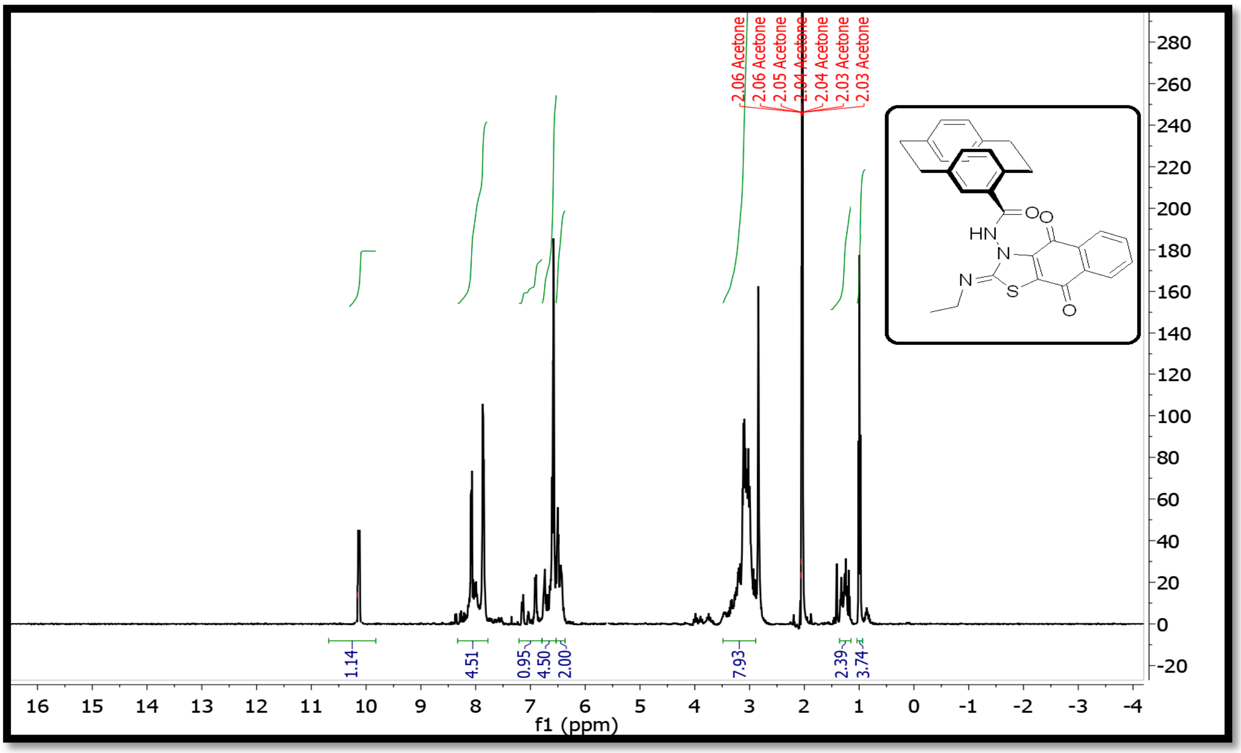


**Figure S13.** $^{1}$H NMR spectrum of compound **3d**

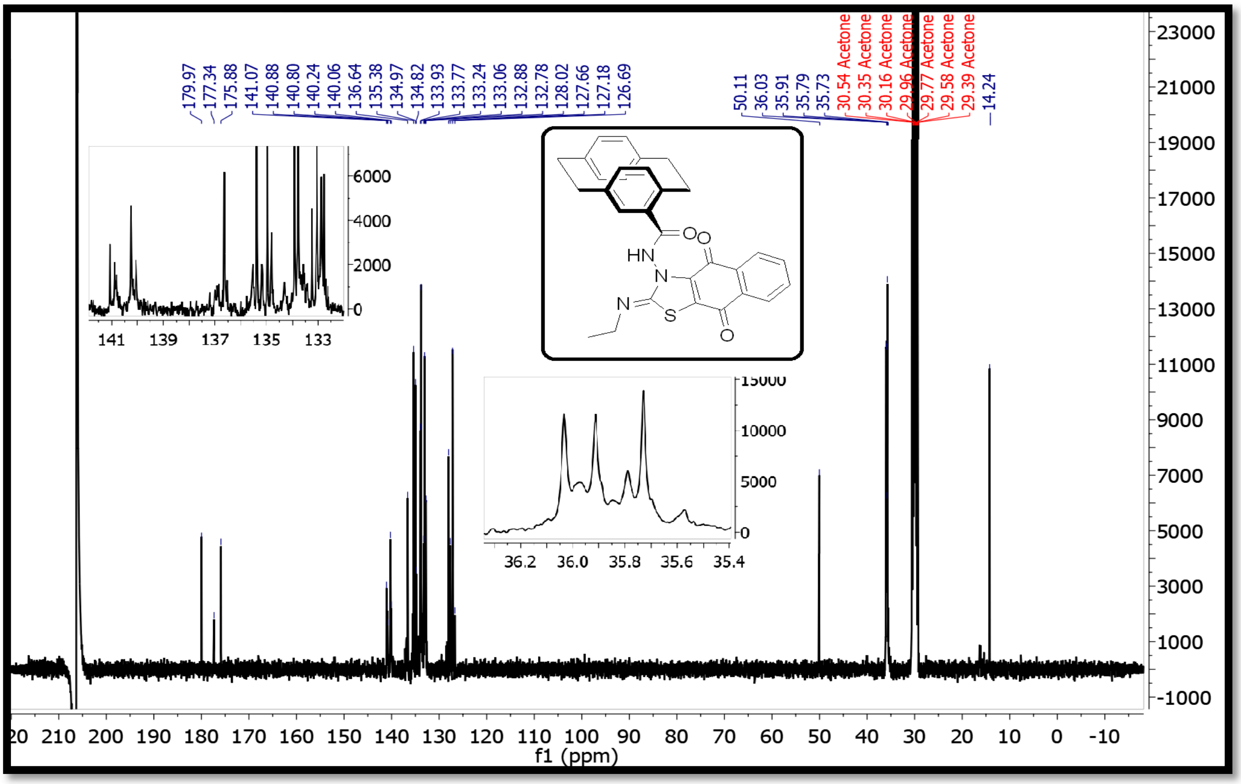

**Figure S14.** $^{13}$C NMR spectrum of compound **3d**

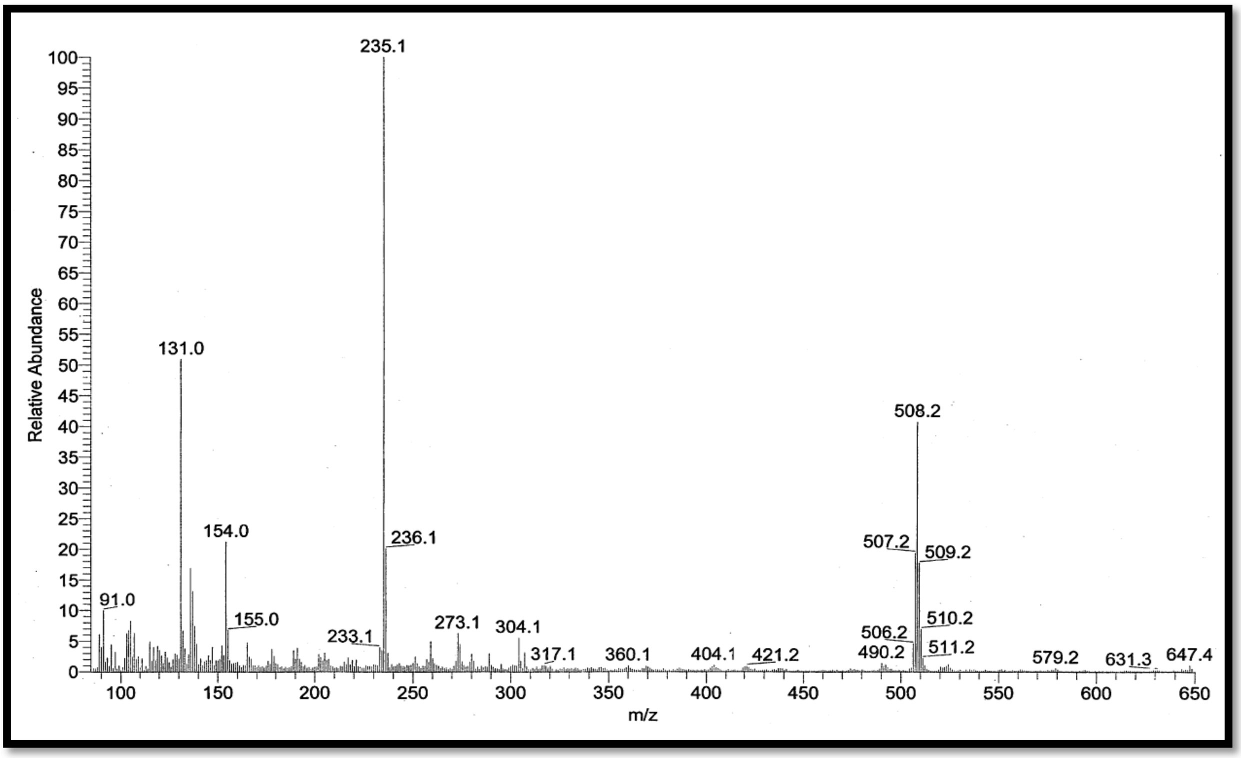

**Figure S15.** Mass spectrum of compound **3d**

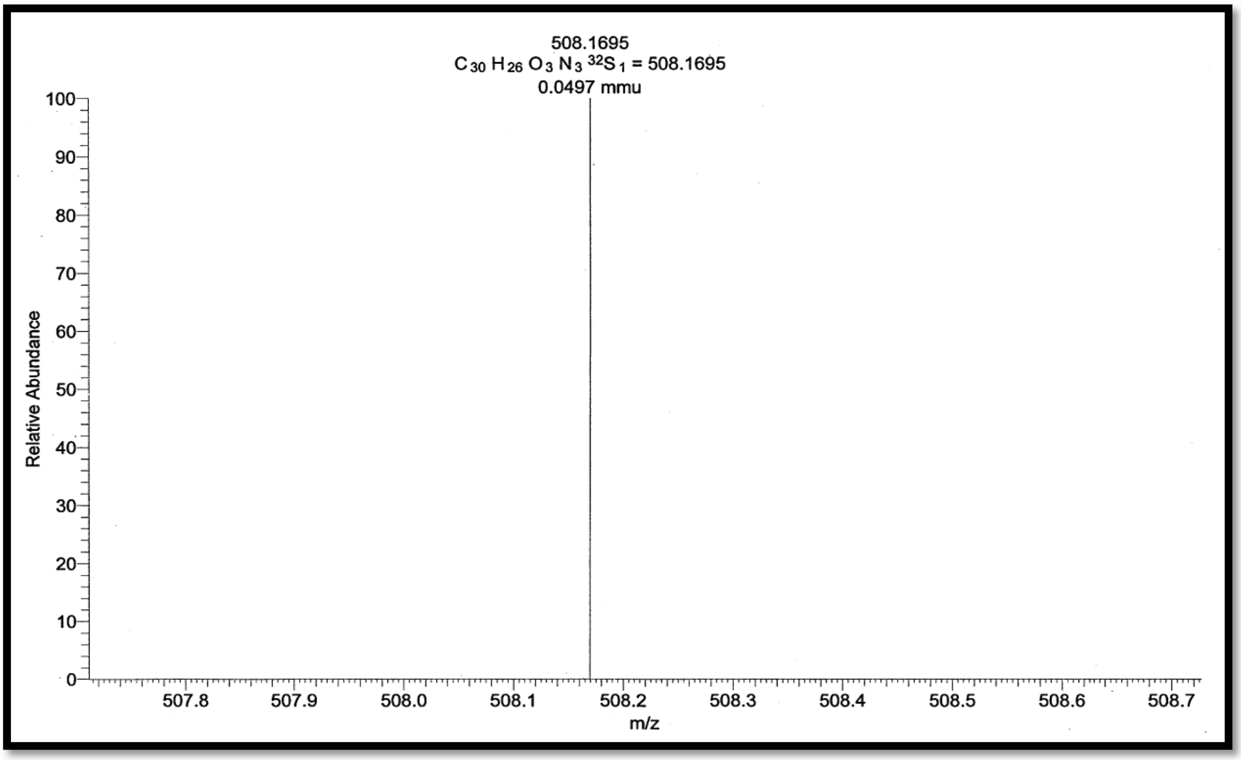


**Figure S16.** HRMS spectrum of compound **3d**

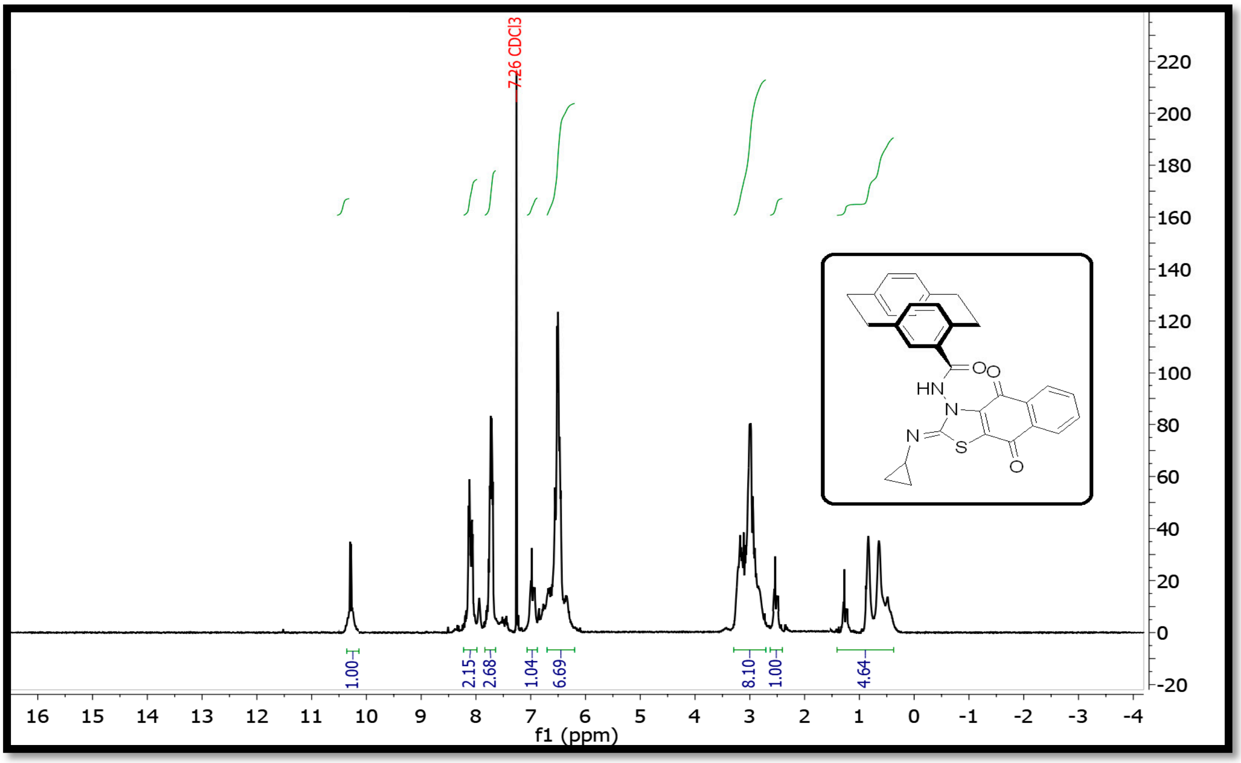


**Figure S17.** $^{1}$H NMR spectrum of compound **3e**

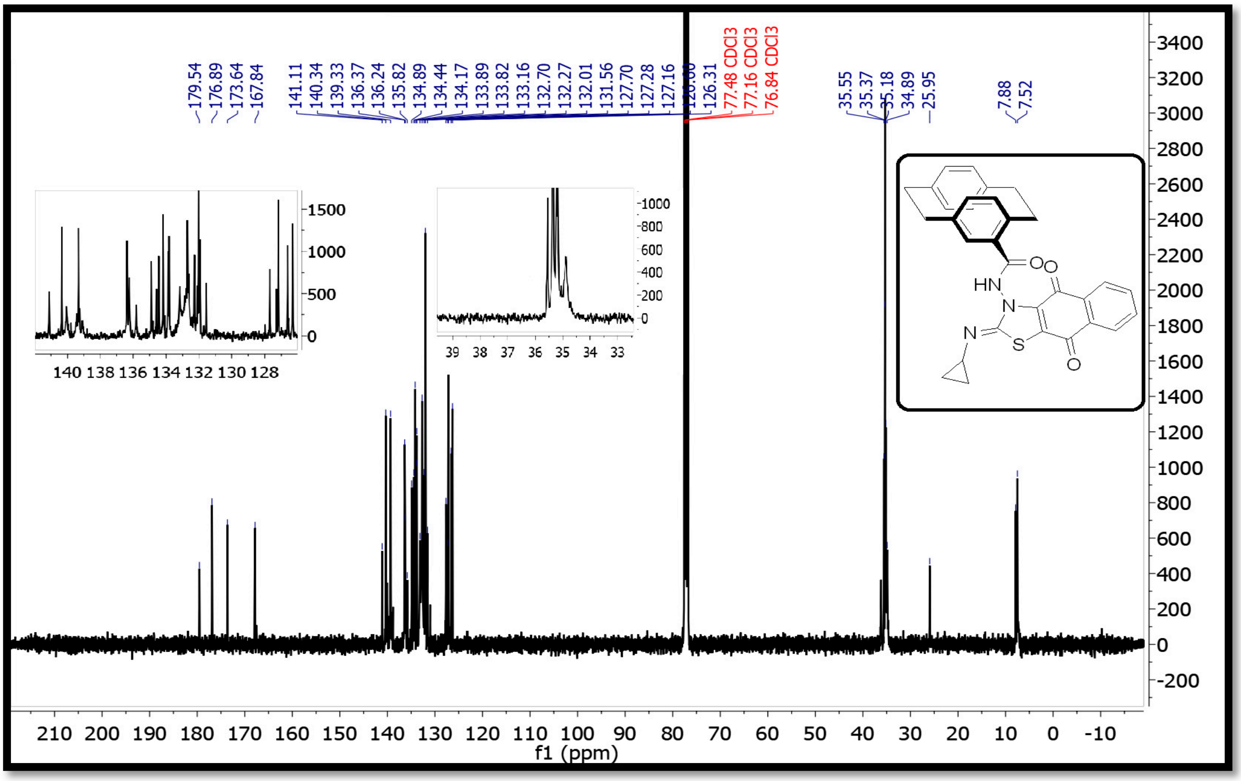


**Figure S18.** $^{13}$C NMR spectrum of compound **3e**

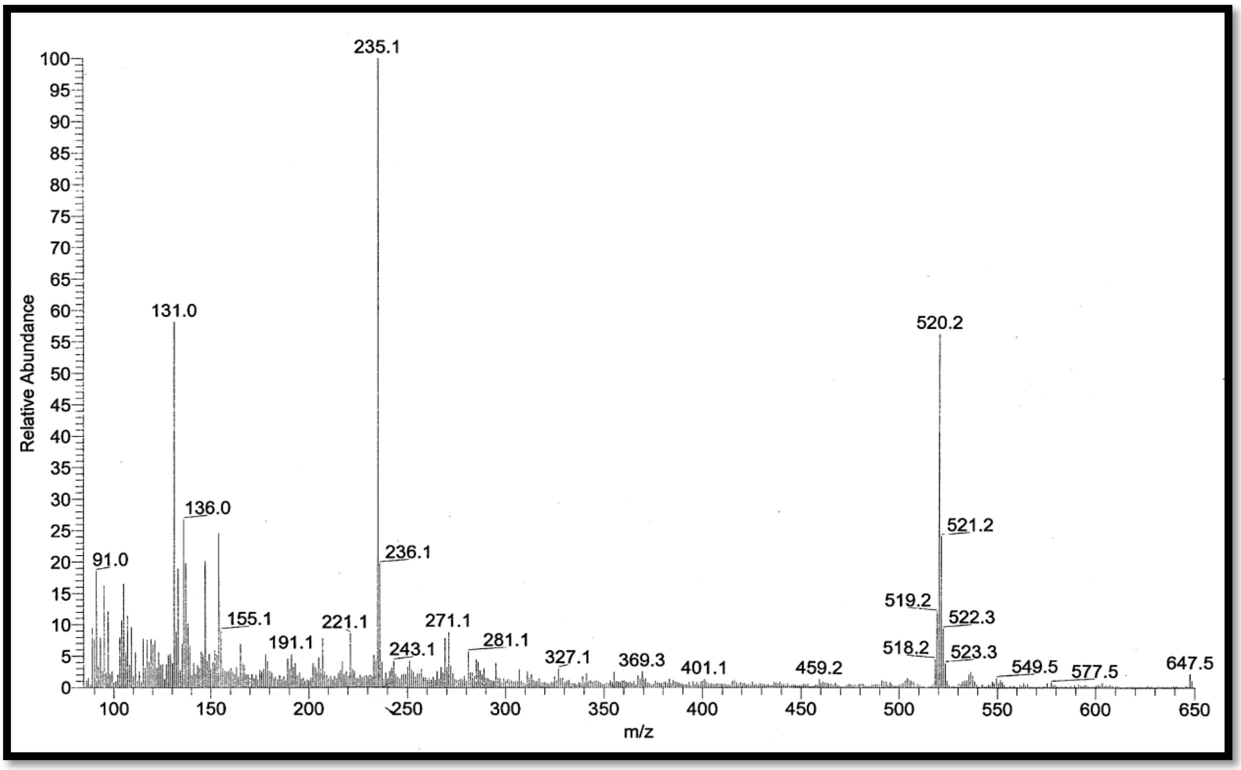


**Figure S19.** Mass spectrum of compound **3e**

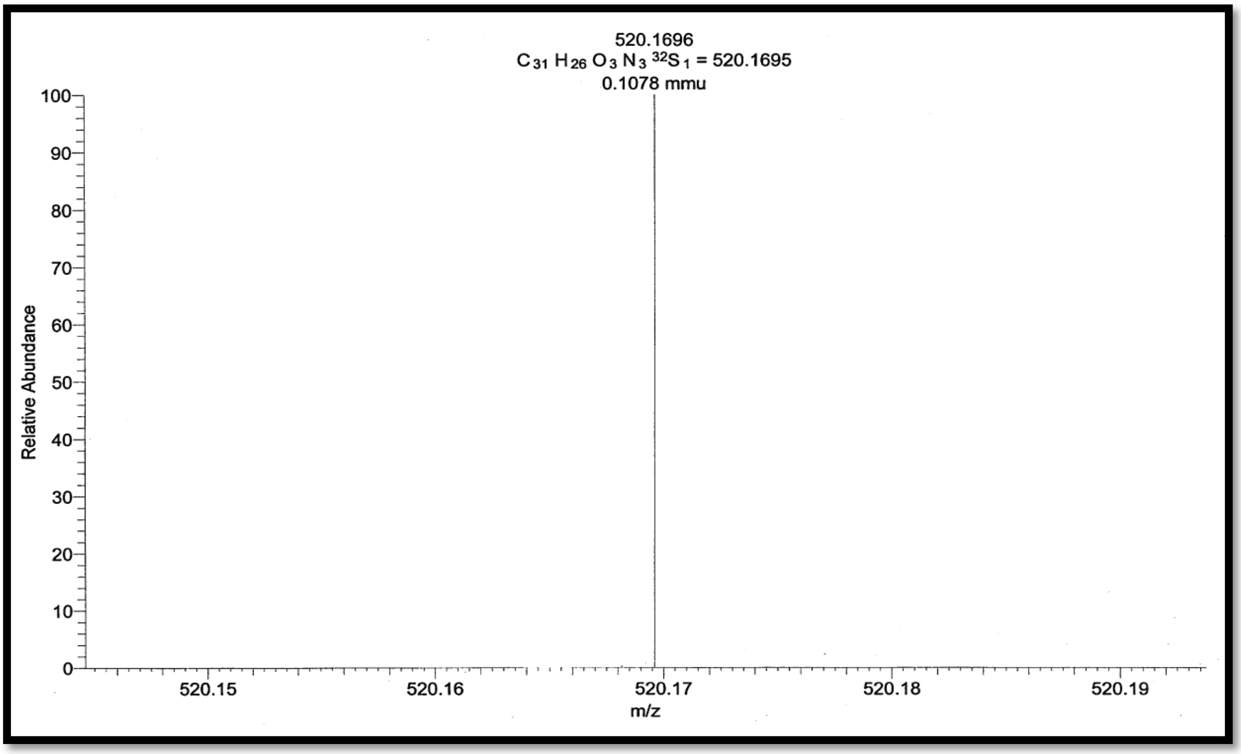


**Figure S20.** HRMS spectrum of compound **3e**

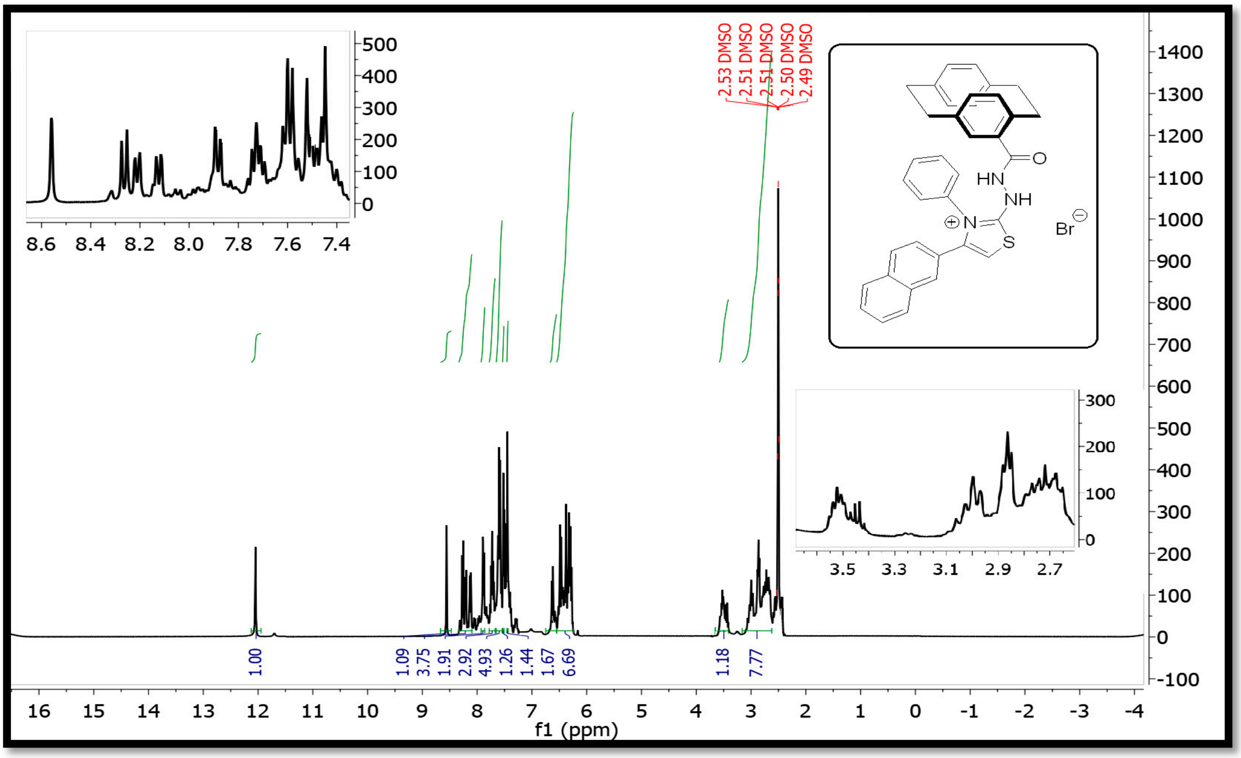


**Figure S21.** $^{1}$H NMR spectrum of compound **8a**

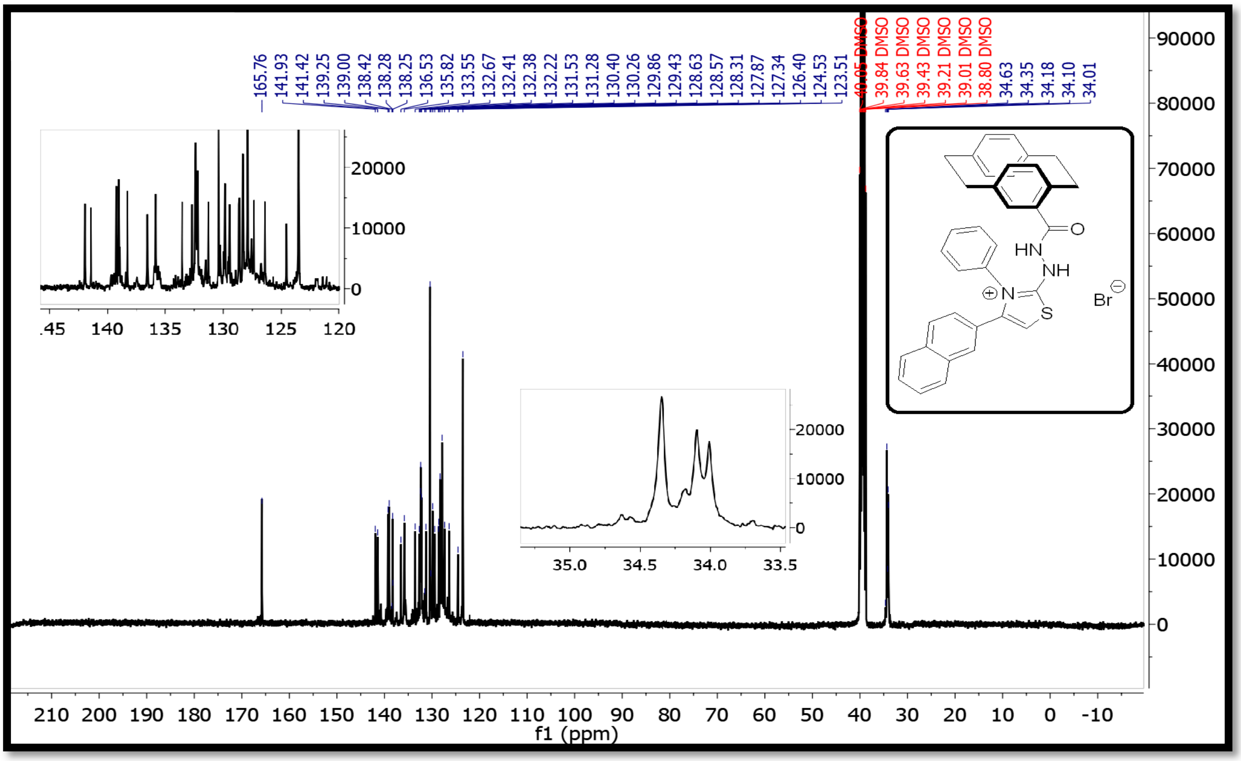


**Figure S22.** $^{13}$C NMR spectrum of compound **8a**

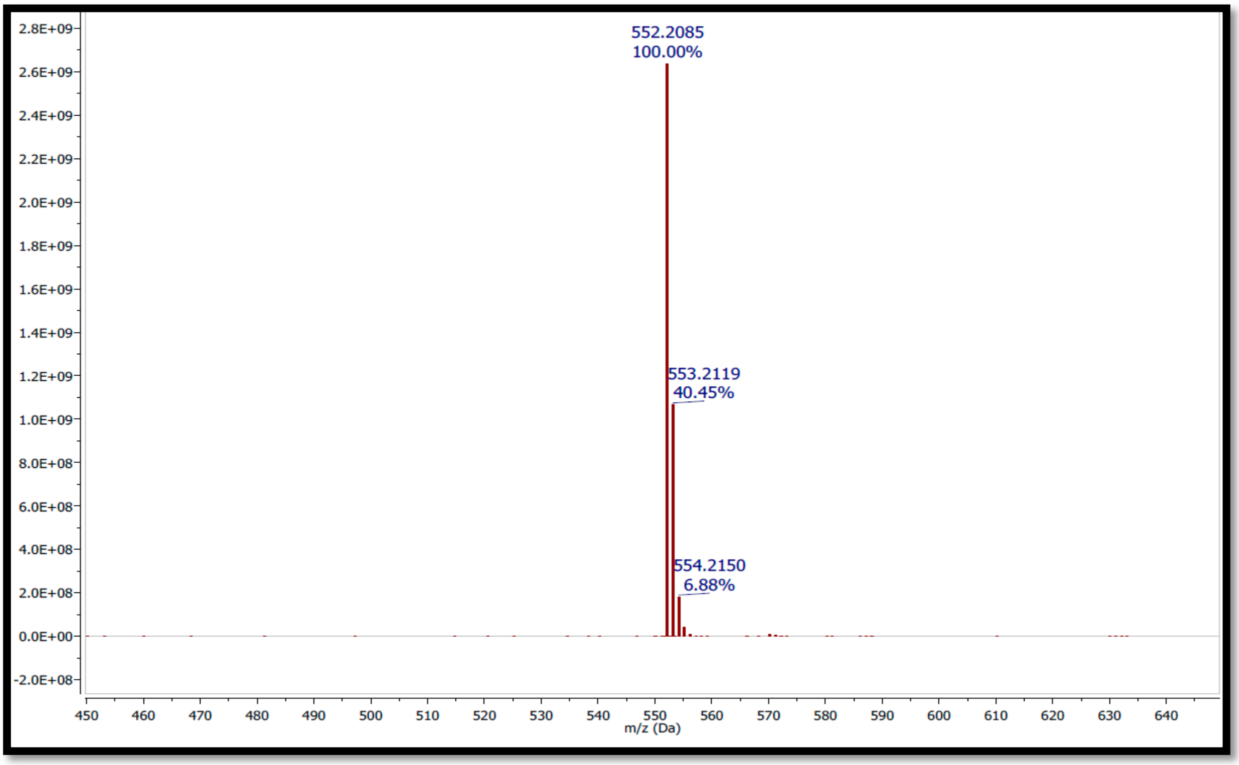


**Figure S23.** HRMS and Mass spectrum of compound **8a**

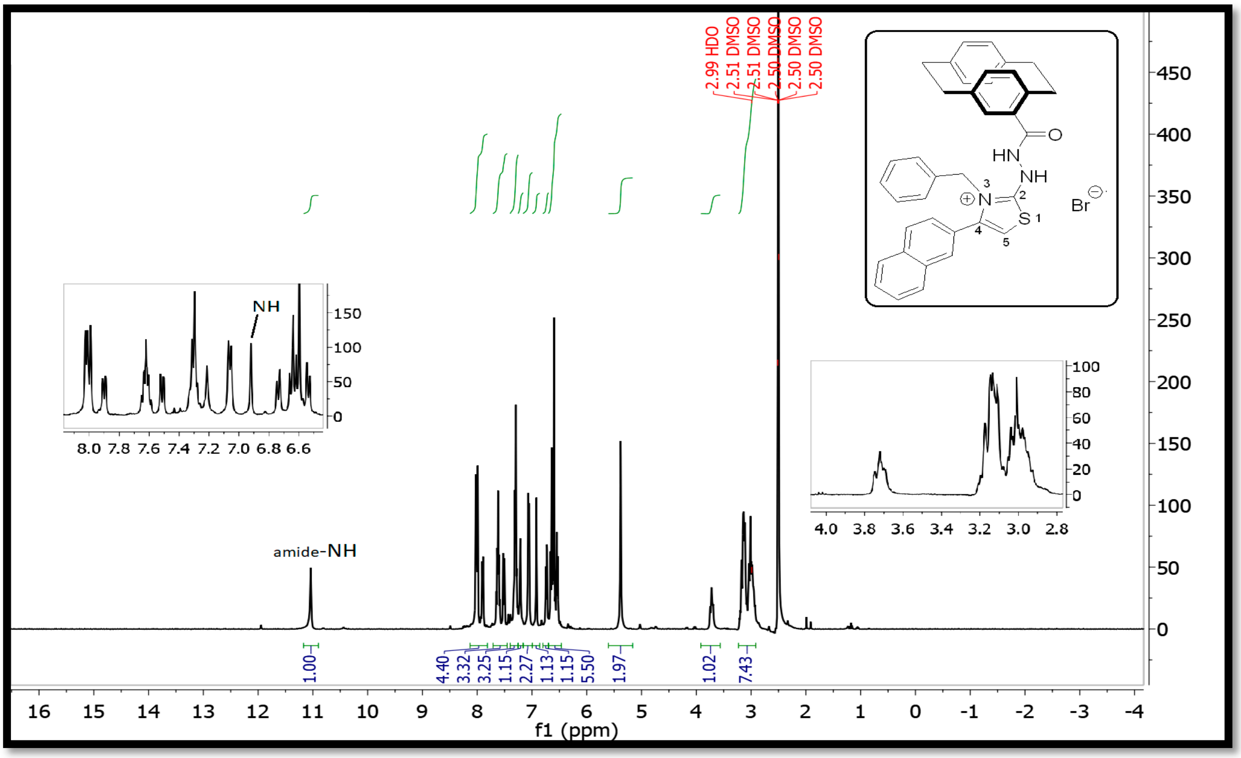


**Figure S24.** $^{1}$H NMR spectrum of compound **8b**

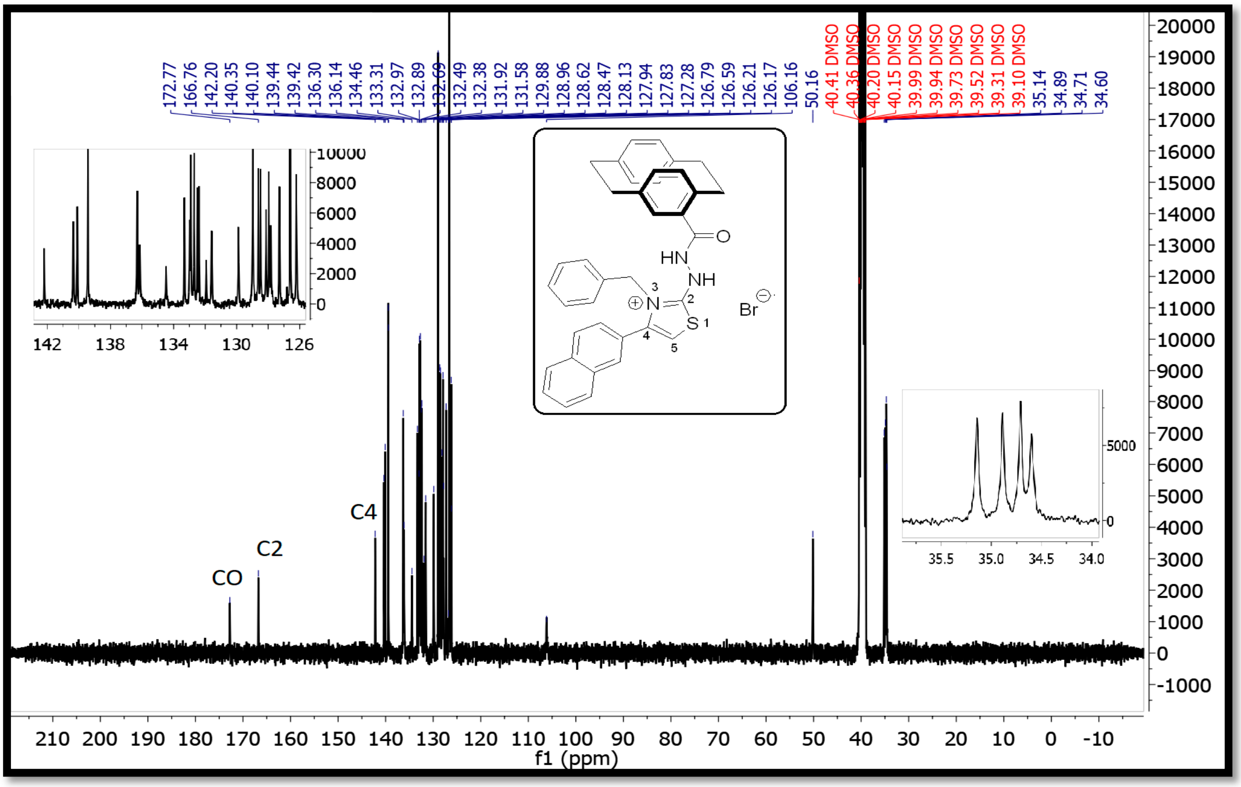


**Figure S25.** $^{13}$C NMR spectrum of compound **8b**

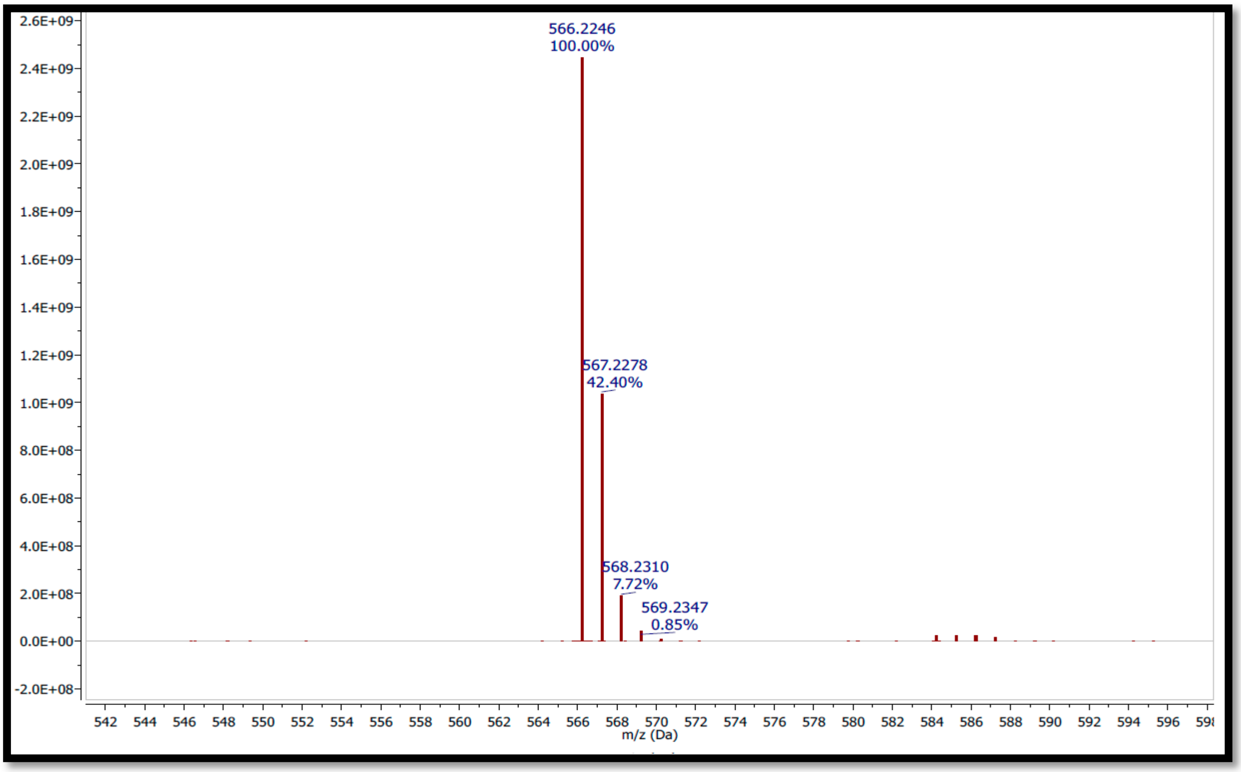


**Figure S26.** HRMS and Mass spectrum of compound **8b**

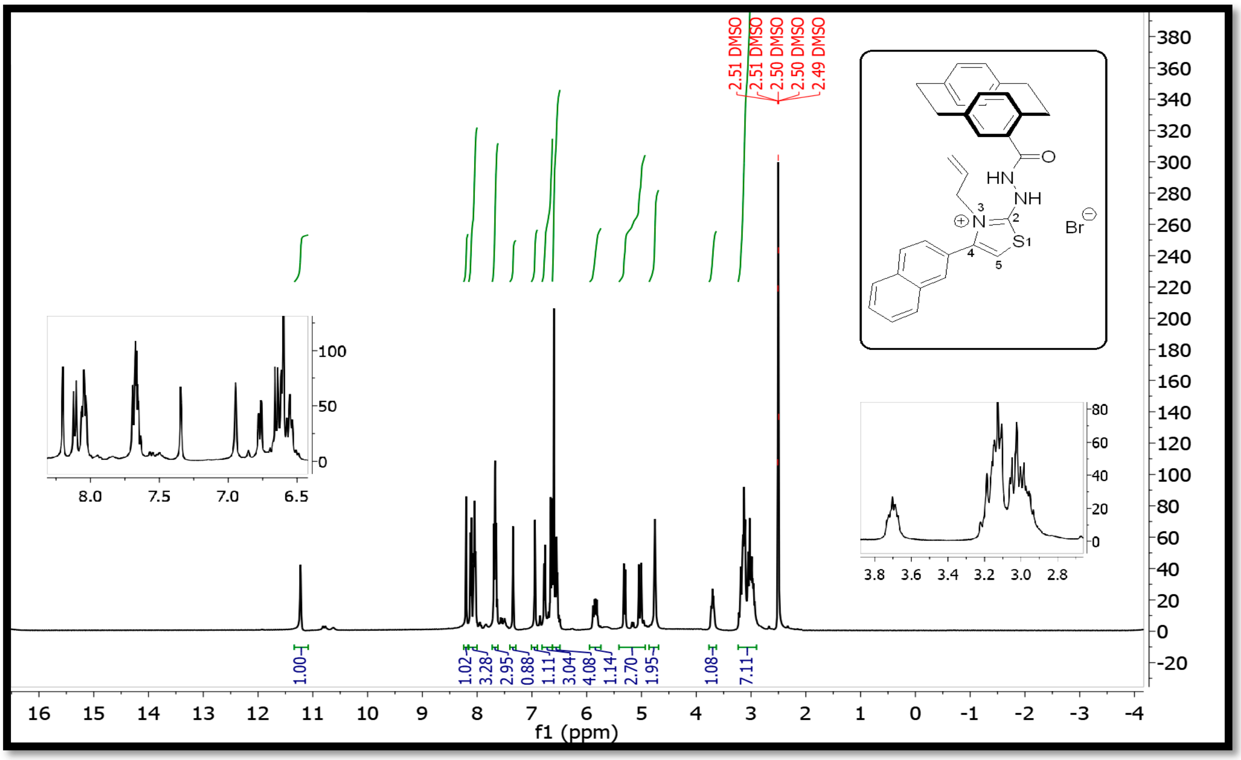


**Figure S27.** $^{1}$H NMR spectrum of compound **8c**

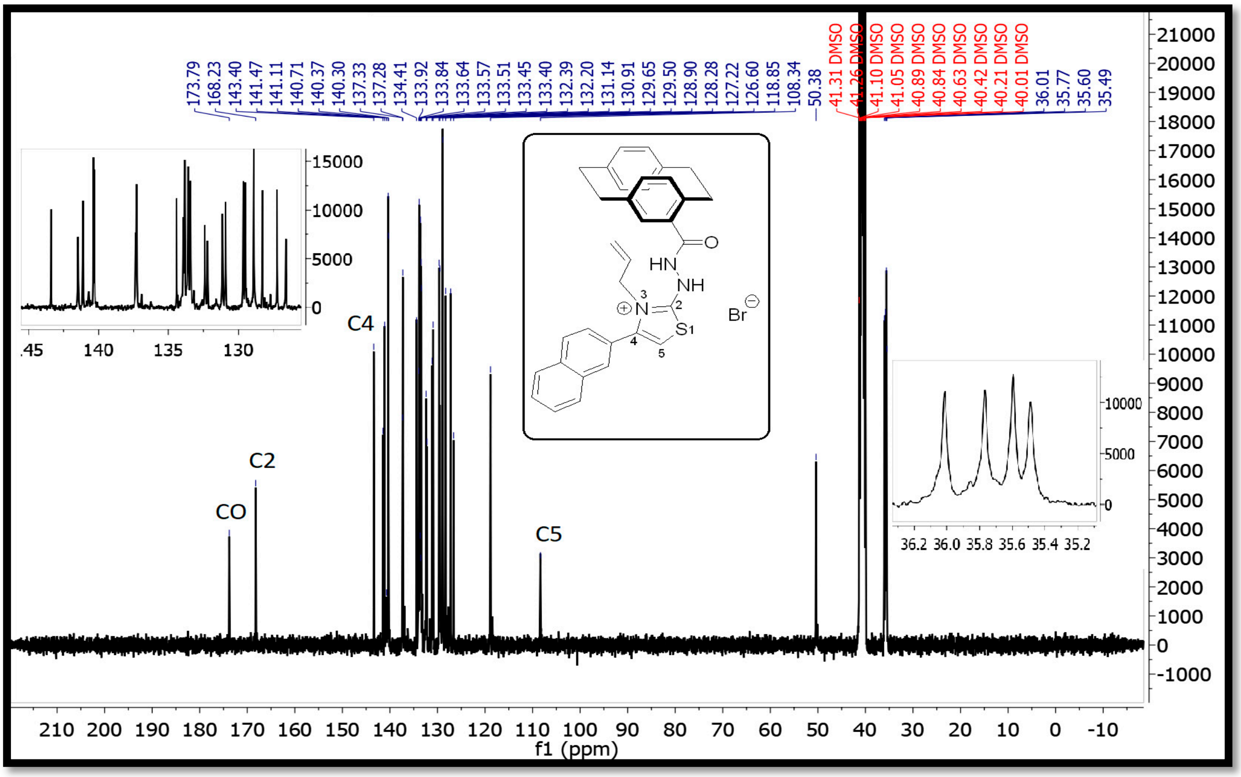


**Figure S28.** $^{13}$C NMR spectrum of compound **8c**

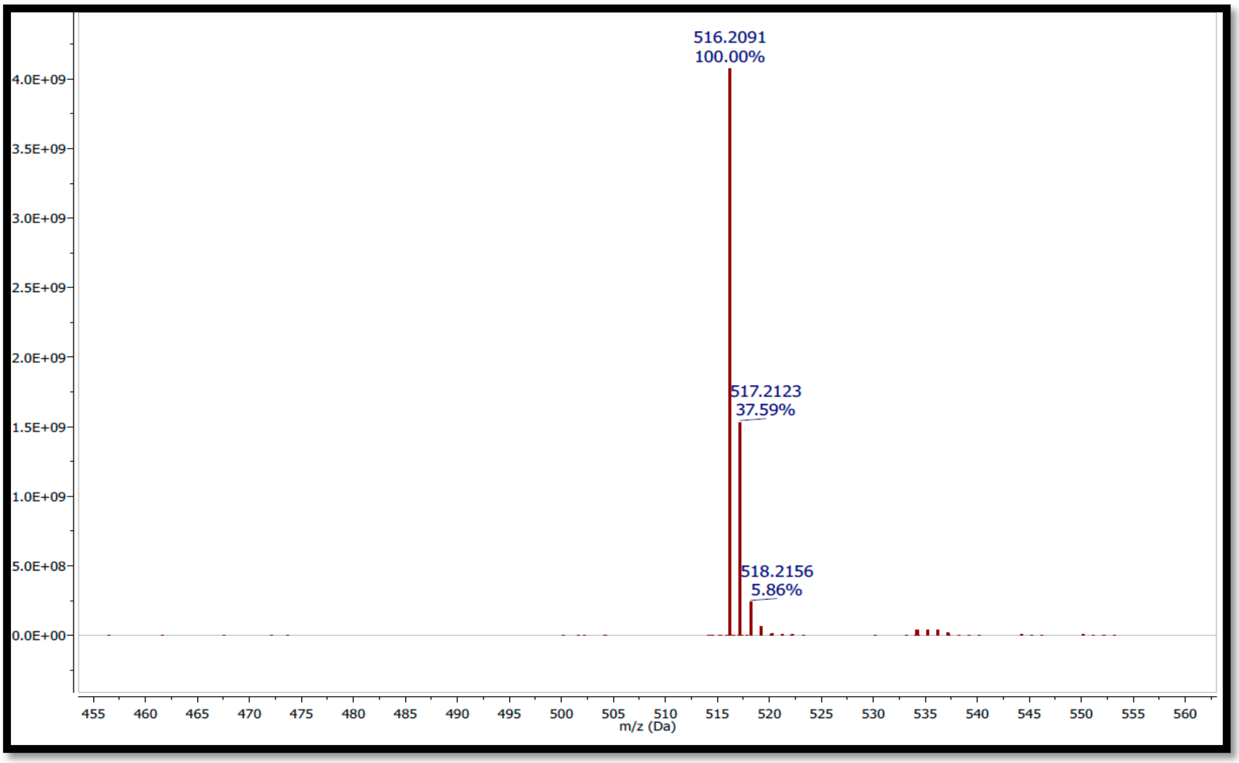


**Figure S29.** HRMS and Mass spectrum of compound **8c**

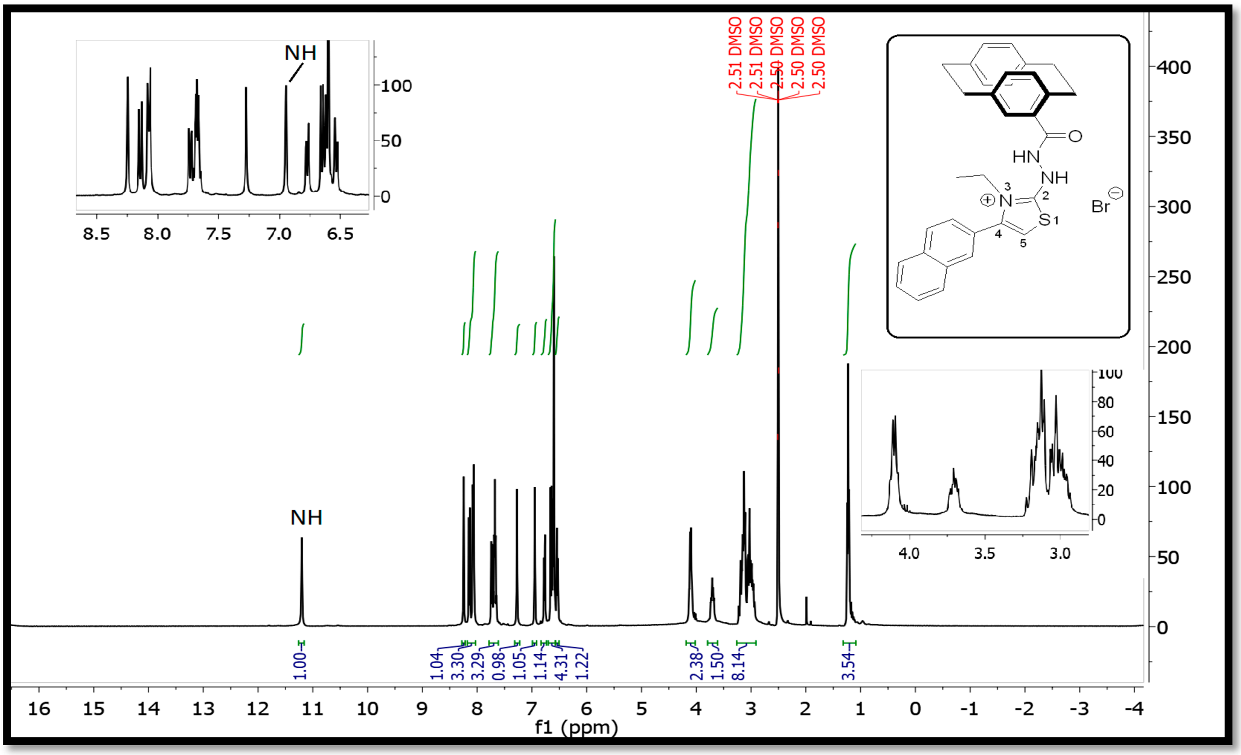

**Figure S30.** $^{1}$H NMR spectrum of compound **8d**

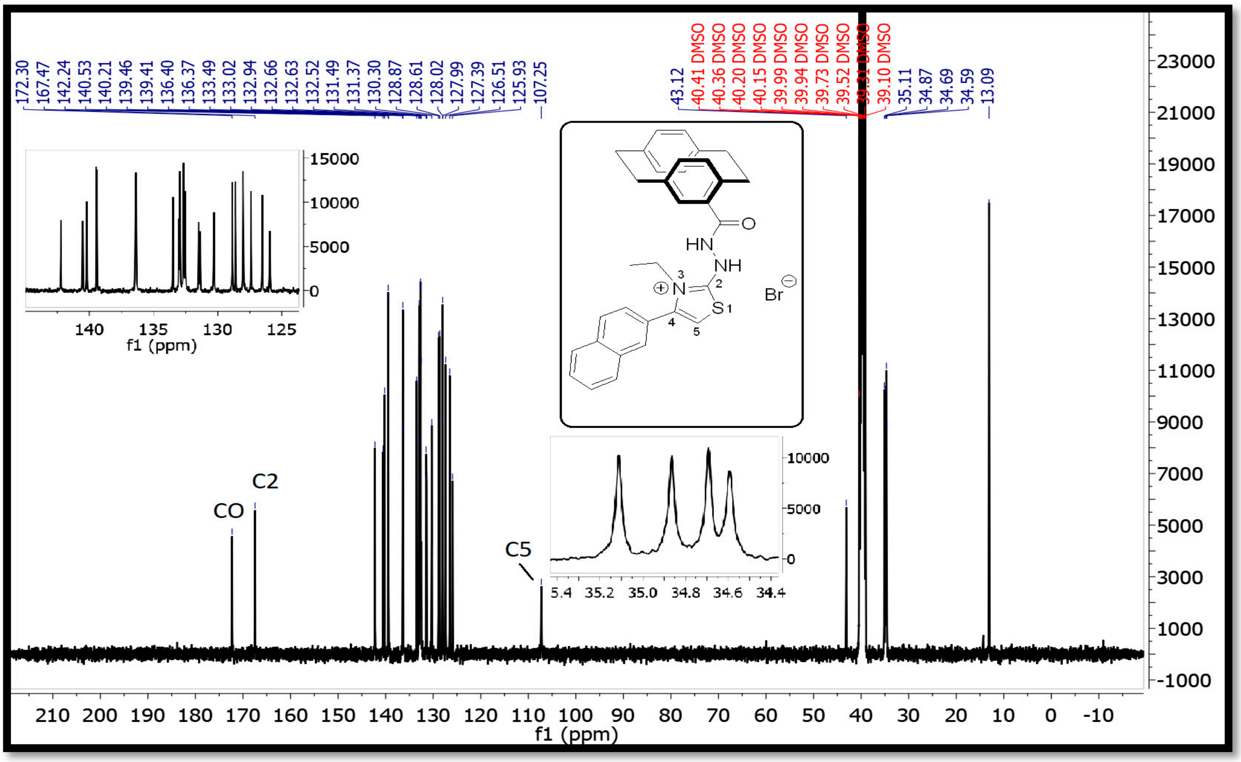

**Figure S31.** $^{13}$C NMR spectrum of compound **8d**

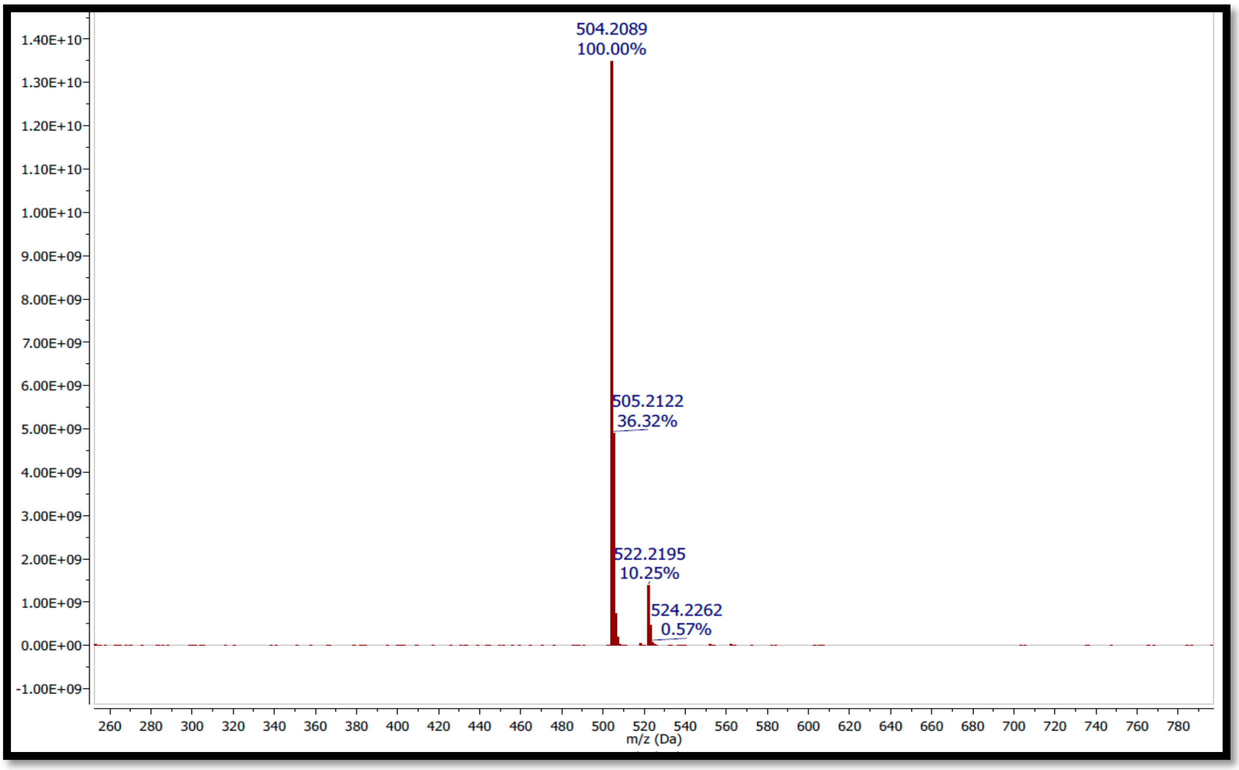


**Figure S32.** HRMS and Mass spectrum of compound **8d**

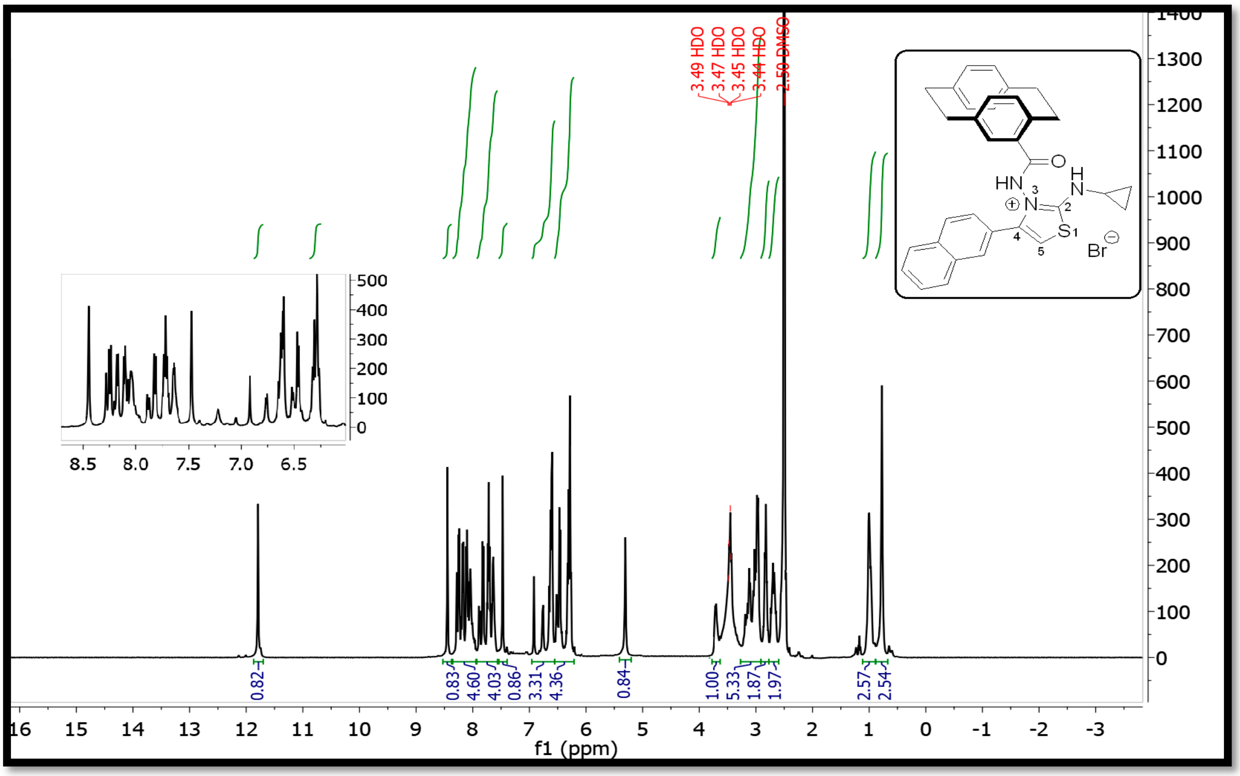


**Figure S33.** $^{1}$H NMR spectrum of compound **9**

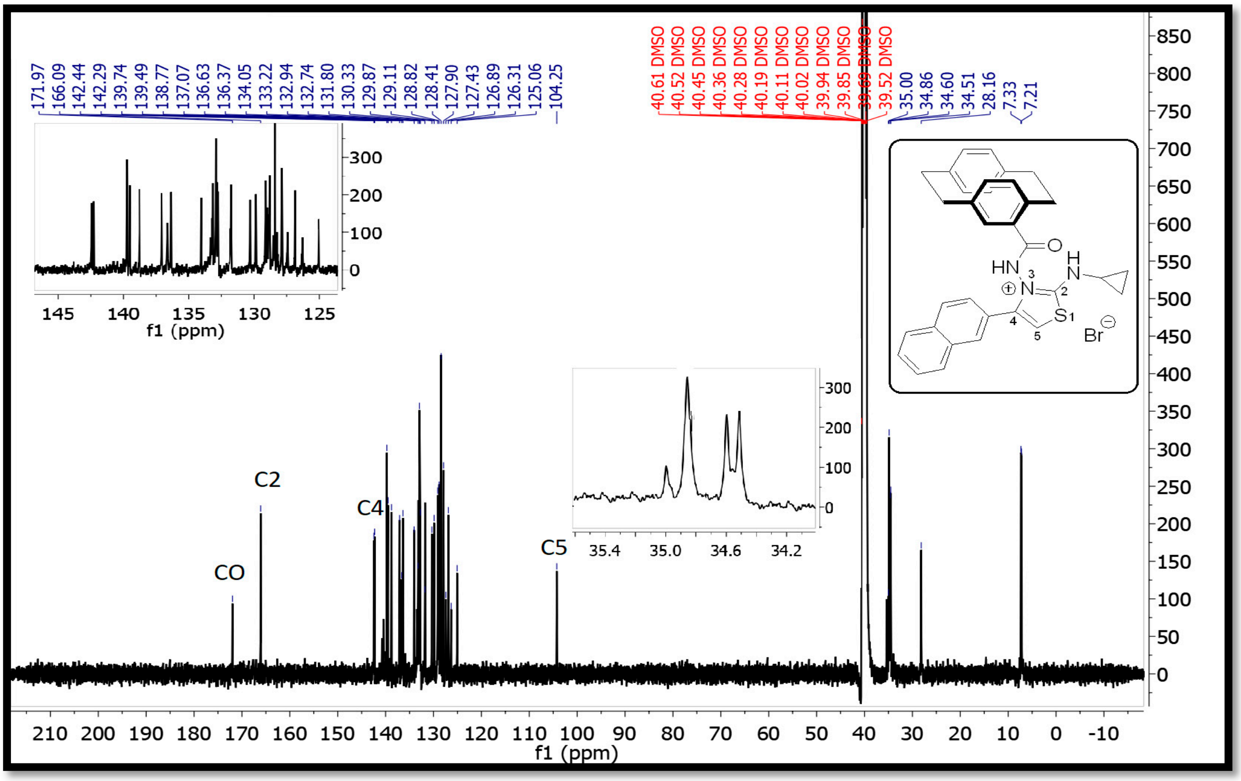


**Figure S34.** $^{13}C$ NMR spectrum of compound **9**

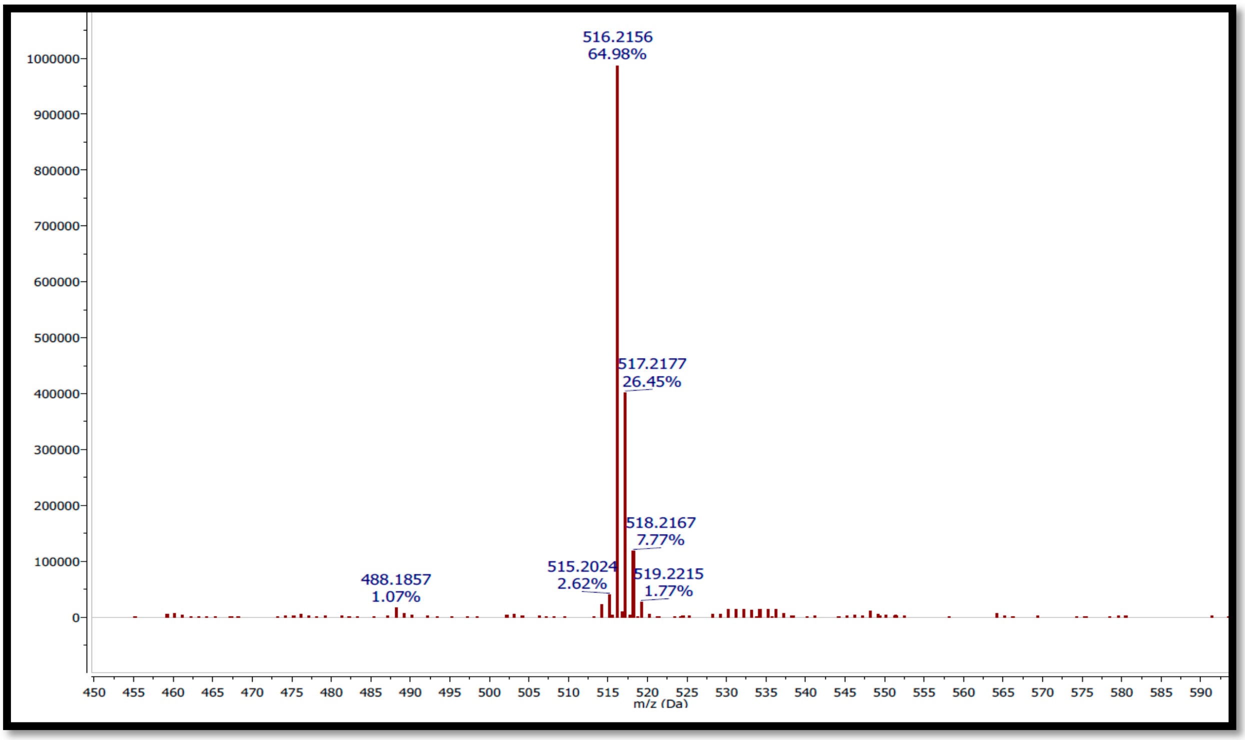


**Figure S35.** HRMS and Mass spectrum of compound **8a**

## 2. Biology

### I- Tables

**Table S1.** DNA content % using propidium iodide flow cytometry.

| **Phase** | **Phase %** | | |
|---|---|---|---|
| | **3c** | **Dinacicilib** | **control** |
| **%G0-G1** | 37.26 | 41.43 | 56.29 |
| **%S** | 26.38 | 29.17 | 31.96 |
| **%G2-M** | 36.36*** | 29.4*** | 11.75 |
| **%Pre G1** | 36.41*** | 32.84*** | 1.61 |

*Results Significantly different from control at ***p < 0.05.*

**Table S2**: Predicted binding scores for compounds **3a-e, 8a-d, 9** and Dinacicilib in CDK1 active site (PDB code: 6GU6).

| Compound | Docking Score (kca/mol) | Compound | Docking Score (kca/mol) |
|---|---|---|---|
| **Dinacicilib** | -10.6 | 8a | -8.8 |
| **3a** | -9.5 | 8b | -8.7 |
| **3b** | -9.4 | 8c | -8.9 |
| **3c** | -9.8 | 8d | -8.7 |
| **3d** | -9.4 | 9 | -8.6 |
| **3e** | -9.3 | | |

**Table S3**: Predicted binding scores for *p*-xylene analogs of compounds **3a-e** in CDK1 active site (PDB code: 6GU6).

| Compound | Docking Score (kca/mol) |
|---|---|
| *p*-xylene **3a** | -8.3 |
| *p*-xylene **3b** | -8.2 |
| *p*-xylene **3c** | -8.7 |
| *p*-xylene **3d** | -8.6 |
| *p*-xylene **3e** | -8.1 |

## II- Figure Ss

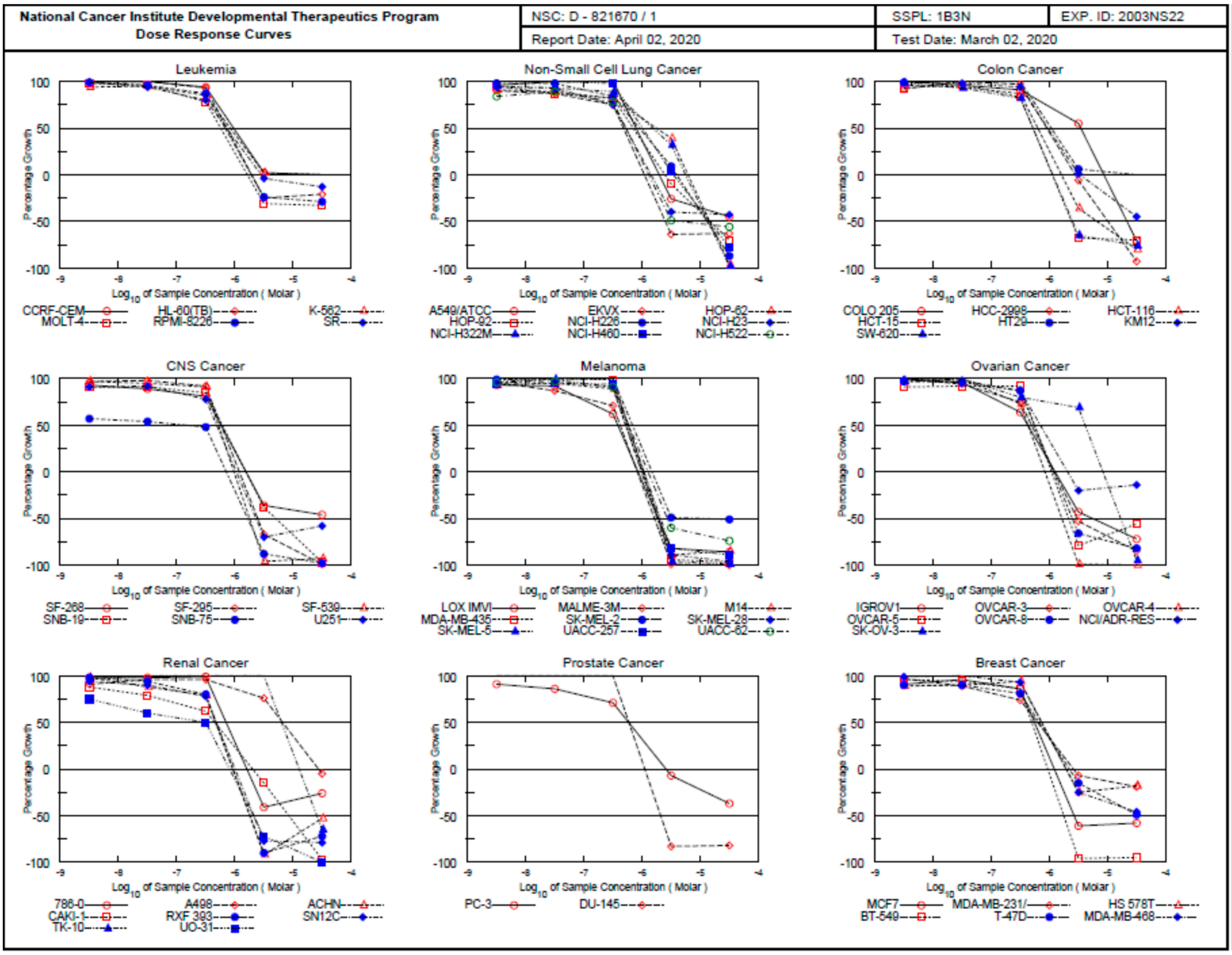


**Figure S36. Dose Response Curves for all cell line for compound 3d**

| National Cancer Institute Developmental Therapeutics Program<br>In-Vitro Testing Results | | | |
|---|---|---|---|
| NSC : D - 821670 / 1 | Experiment ID : 2003NS22 | Test Type : 08 | Units : Molar |
| Report Date : April 02, 2020 | Test Date : March 02, 2020 | QNS : | MC : |
| COMI : LE142 | Stain Reagent : SRB Dual-Pass Related | SSPL : 1B3N | |

| | | | Log10 Concentration | | | | | | | | | | | | |
|---|---|---|---|---|---|---|---|---|---|---|---|---|---|---|---|
| | Time | | Mean Optical Densities | | | | | Percent Growth | | | | | | | |
| Panel/Cell Line | Zero | Ctrl | -8.5 | -7.5 | -6.5 | -5.5 | -4.5 | -8.5 | -7.5 | -6.5 | -5.5 | -4.5 | GI50 | TGI | LC50 |
| Leukemia | | | | | | | | | | | | | | | |
| CCRF-CEM | 0.510 | 2.658 | 2.684 | 2.733 | 2.525 | 0.551 | 0.513 | 101 | 103 | 94 | 2 | . | 9.98E-7 | > 3.33E-5 | > 3.33E-5 |
| HL-60(TB) | 0.825 | 3.183 | 3.154 | 3.177 | 3.012 | 0.622 | 0.650 | 99 | 100 | 93 | -25 | -21 | 7.70E-7 | 2.05E-6 | > 3.33E-5 |
| K-562 | 0.235 | 2.239 | 2.127 | 2.138 | 1.935 | 0.279 | 0.238 | 94 | 95 | 85 | 2 | . | 8.79E-7 | > 3.33E-5 | > 3.33E-5 |
| MOLT-4 | 0.763 | 3.141 | 3.098 | 3.043 | 2.613 | 0.525 | 0.514 | 98 | 96 | 78 | -31 | -33 | 5.99E-7 | 1.72E-6 | > 3.33E-5 |
| RPMI-8226 | 0.711 | 2.867 | 2.841 | 2.789 | 2.591 | 0.537 | 0.507 | 99 | 96 | 87 | -24 | -29 | 7.17E-7 | 2.01E-6 | > 3.33E-5 |
| SR | 0.391 | 1.744 | 1.730 | 1.665 | 1.476 | 0.374 | 0.342 | 99 | 94 | 80 | -4 | -13 | 7.57E-7 | 2.95E-6 | > 3.33E-5 |
| Non-Small Cell Lung Cancer | | | | | | | | | | | | | | | |
| A549/ATCC | 0.425 | 2.293 | 2.221 | 2.332 | 2.306 | 0.313 | 0.233 | 96 | 102 | 101 | -26 | -45 | 8.35E-7 | 2.07E-6 | > 3.33E-5 |
| EKVX | 0.561 | 2.078 | 1.923 | 1.879 | 1.691 | 0.202 | 0.209 | 90 | 87 | 75 | -64 | -63 | 5.01E-7 | 1.15E-6 | 2.64E-6 |
| HOP-62 | 1.398 | 2.939 | 2.805 | 2.765 | 2.668 | 1.998 | 0.061 | 91 | 89 | 82 | 39 | -96 | 1.85E-6 | 6.48E-6 | 1.53E-5 |
| HOP-92 | 1.117 | 1.845 | 1.810 | 1.754 | 1.713 | 1.008 | 0.339 | 95 | 87 | 82 | -10 | -70 | 7.41E-7 | 2.60E-6 | 1.56E-5 |
| NCI-H226 | 0.785 | 2.033 | 2.007 | 2.012 | 1.831 | 0.892 | 0.103 | 98 | 98 | 84 | 9 | -87 | 9.36E-7 | 4.09E-6 | 1.37E-5 |
| NCI-H23 | 0.897 | 2.486 | 2.396 | 2.379 | 2.089 | 0.541 | 0.510 | 94 | 93 | 75 | -40 | -43 | 5.50E-7 | 1.50E-6 | > 3.33E-5 |
| NCI-H322M | 0.676 | 1.772 | 1.712 | 1.697 | 1.646 | 1.030 | 0.016 | 95 | 93 | 89 | 32 | -98 | 1.61E-6 | 5.90E-6 | 1.43E-5 |
| NCI-H460 | 0.317 | 2.930 | 2.962 | 3.018 | 2.867 | 0.390 | 0.070 | 101 | 103 | 98 | 3 | -78 | 1.06E-6 | 3.60E-6 | 1.50E-5 |
| NCI-H522 | 0.874 | 2.183 | 1.979 | 2.038 | 1.890 | 0.446 | 0.384 | 84 | 89 | 78 | -49 | -56 | 5.51E-7 | 1.37E-6 | 4.64E-6 |
| Colon Cancer | | | | | | | | | | | | | | | |
| COLO 205 | 0.722 | 2.900 | 2.893 | 2.813 | 2.709 | 1.915 | 0.208 | 100 | 96 | 91 | 55 | -71 | 3.63E-6 | 9.06E-6 | 2.26E-5 |
| HCC-2998 | 0.774 | 2.533 | 2.394 | 2.612 | 2.473 | 0.728 | 0.052 | 92 | 104 | 97 | -6 | -93 | 9.48E-7 | 2.91E-6 | 1.06E-5 |
| HCT-116 | 0.304 | 2.842 | 2.750 | 2.701 | 2.520 | 0.194 | 0.061 | 96 | 94 | 87 | -36 | -80 | 6.67E-7 | 1.70E-6 | 6.89E-6 |
| HCT-15 | 0.260 | 2.399 | 2.248 | 2.334 | 2.028 | 0.084 | 0.079 | 93 | 97 | 83 | -68 | -70 | 5.49E-7 | 1.18E-6 | 2.54E-6 |
| HT29 | 0.396 | 2.543 | 2.518 | 2.590 | 2.566 | 0.533 | 0.396 | 99 | 102 | 101 | 6 | . | 1.15E-6 | 3.18E-5 | > 3.33E-5 |
| KM12 | 0.643 | 3.135 | 3.098 | 3.093 | 2.997 | 0.676 | 0.354 | 99 | 98 | 94 | 1 | -45 | 9.99E-7 | 3.56E-6 | > 3.33E-5 |
| SW-620 | 0.360 | 2.637 | 2.601 | 2.471 | 2.234 | 0.125 | 0.086 | 98 | 93 | 82 | -65 | -76 | 5.51E-7 | 1.20E-6 | 2.62E-6 |
| CNS Cancer | | | | | | | | | | | | | | | |
| SF-268 | 0.756 | 2.524 | 2.399 | 2.329 | 2.190 | 0.487 | 0.412 | 93 | 89 | 81 | -36 | -46 | 6.15E-7 | 1.65E-6 | > 3.33E-5 |
| SF-295 | 0.953 | 3.229 | 3.163 | 3.177 | 3.058 | 0.314 | 0.012 | 97 | 98 | 92 | -67 | -99 | 6.15E-7 | 1.27E-6 | 2.60E-6 |
| SF-539 | 0.702 | 2.401 | 2.354 | 2.309 | 2.255 | 0.029 | 0.048 | 97 | 95 | 91 | -96 | -93 | 5.54E-7 | 1.02E-6 | 1.89E-6 |
| SNB-19 | 0.901 | 2.530 | 2.381 | 2.391 | 2.293 | 0.558 | 0.029 | 91 | 91 | 85 | -38 | -97 | 6.45E-7 | 1.64E-6 | 5.31E-6 |
| SNB-75 | 1.913 | 2.894 | 2.471 | 2.439 | 2.380 | 0.223 | 0.031 | 57 | 54 | 48 | -88 | -98 | 1.32E-7 | 7.46E-7 | 1.74E-6 |
| U251 | 0.417 | 2.043 | 1.893 | 1.916 | 1.678 | 0.125 | 0.176 | 91 | 92 | 78 | -70 | -58 | 5.12E-7 | 1.12E-6 | 2.43E-6 |
| Melanoma | | | | | | | | | | | | | | | |
| LOX IMVI | 0.342 | 2.445 | 2.302 | 2.267 | 1.655 | 0.063 | 0.049 | 93 | 92 | 62 | -82 | -86 | 4.06E-7 | 9.04E-7 | 2.01E-6 |
| MALME-3M | 0.733 | 1.265 | 1.245 | 1.197 | 1.110 | 0.008 | -0.005 | 96 | 87 | 71 | -99 | -100 | 4.42E-7 | 8.70E-7 | 1.71E-6 |
| M14 | 0.498 | 2.251 | 2.136 | 2.160 | 2.066 | 0.055 | 0.075 | 93 | 95 | 89 | -89 | -85 | 5.54E-7 | 1.06E-6 | 2.01E-6 |
| MDA-MB-435 | 0.572 | 2.542 | 2.447 | 2.595 | 2.526 | 0.034 | 0.016 | 95 | 103 | 99 | -94 | -97 | 5.98E-7 | 1.09E-6 | 1.97E-6 |
| SK-MEL-2 | 1.118 | 2.596 | 2.577 | 2.619 | 2.633 | 0.571 | 0.546 | 99 | 102 | 102 | -49 | -51 | 7.39E-7 | 1.58E-6 | 9.60E-6 |
| SK-MEL-28 | 0.802 | 2.118 | 2.159 | 2.235 | 2.296 | 0.092 | 0.030 | 103 | 109 | 113 | -89 | -96 | 6.87E-7 | 1.21E-6 | 2.15E-6 |
| SK-MEL-5 | 0.878 | 3.175 | 3.113 | 3.150 | 2.966 | 0.034 | 0.011 | 97 | 99 | 91 | -96 | -99 | 5.51E-7 | 1.02E-6 | 1.89E-6 |
| UACC-257 | 1.032 | 2.464 | 2.379 | 2.394 | 2.378 | 0.190 | 0.109 | 94 | 95 | 94 | -82 | -89 | 5.93E-7 | 1.14E-6 | 2.20E-6 |
| UACC-62 | 1.131 | 2.967 | 2.885 | 2.917 | 2.807 | 0.448 | 0.299 | 96 | 97 | 91 | -60 | -74 | 6.23E-7 | 1.33E-6 | 2.84E-6 |
| Ovarian Cancer | | | | | | | | | | | | | | | |
| IGROV1 | 0.483 | 1.984 | 2.032 | 1.931 | 1.438 | 0.275 | 0.135 | 103 | 96 | 64 | -43 | -72 | 4.46E-7 | 1.31E-6 | 5.78E-6 |
| OVCAR-3 | 0.492 | 1.714 | 1.729 | 1.645 | 1.408 | 0.232 | 0.072 | 101 | 94 | 75 | -53 | -85 | 5.22E-7 | 1.28E-6 | 3.16E-6 |
| OVCAR-4 | 1.065 | 1.998 | 1.971 | 2.020 | 1.748 | 0.013 | 0.004 | 97 | 102 | 73 | -99 | -100 | 4.54E-7 | 8.87E-7 | 1.73E-6 |
| OVCAR-5 | 0.797 | 2.041 | 1.926 | 1.946 | 1.941 | 0.169 | 0.349 | 91 | 92 | 92 | -79 | -56 | 5.86E-7 | 1.15E-6 | 2.26E-6 |
| OVCAR-8 | 0.451 | 2.377 | 2.311 | 2.295 | 2.125 | 0.155 | 0.081 | 97 | 96 | 87 | -66 | -82 | 5.81E-7 | 1.24E-6 | 2.63E-6 |
| NCI/ADR-RES | 0.567 | 2.184 | 2.170 | 2.154 | 1.979 | 0.455 | 0.486 | 99 | 98 | 87 | -20 | -14 | 7.43E-7 | 2.17E-6 | > 3.33E-5 |
| SK-OV-3 | 1.041 | 2.044 | 2.012 | 2.070 | 1.842 | 1.734 | 0.048 | 97 | 103 | 80 | 69 | -95 | 4.35E-6 | 8.75E-6 | 1.76E-5 |
| Renal Cancer | | | | | | | | | | | | | | | |
| 786-0 | 1.016 | 3.077 | 3.031 | 3.046 | 3.063 | 0.599 | 0.755 | 98 | 98 | 99 | -41 | -26 | 7.48E-7 | 1.70E-6 | > 3.33E-5 |
| A498 | 1.819 | 2.610 | 2.541 | 2.576 | 2.580 | 2.417 | 1.731 | 91 | 96 | 96 | 76 | -5 | 6.93E-6 | 2.90E-5 | > 3.33E-5 |
| ACHN | 0.368 | 1.804 | 1.770 | 1.632 | 1.503 | 0.030 | 0.173 | 98 | 88 | 79 | -92 | -53 | 4.92E-7 | 9.65E-7 | 1.89E-6 |
| CAKI-1 | 0.530 | 1.911 | 1.751 | 1.620 | 1.389 | 0.452 | 0.019 | 88 | 79 | 62 | -15 | -97 | 4.80E-7 | 2.14E-6 | 8.98E-6 |
| RXF 393 | 0.962 | 1.803 | 1.787 | 1.755 | 1.639 | 0.095 | 0.266 | 98 | 94 | 80 | -90 | -72 | 5.02E-7 | 9.86E-7 | 1.94E-6 |
| SN12C | 0.763 | 2.720 | 2.605 | 2.516 | 2.293 | 0.178 | 0.157 | 94 | 90 | 78 | -77 | -79 | 5.06E-7 | 1.07E-6 | 2.24E-6 |
| TK-10 | 1.162 | 1.919 | 1.893 | 2.112 | 2.435 | 2.380 | 0.404 | 97 | 126 | 168 | 161 | -65 | 1.03E-5 | 1.71E-5 | 2.85E-5 |
| UO-31 | 0.618 | 1.662 | 1.398 | 1.248 | 1.142 | 0.176 | -0.014 | 75 | 60 | 50 | -72 | -100 | 3.34E-7 | 8.60E-7 | 2.21E-6 |
| Prostate Cancer | | | | | | | | | | | | | | | |
| PC-3 | 0.560 | 1.738 | 1.629 | 1.573 | 1.399 | 0.519 | 0.352 | 91 | 86 | 71 | -7 | -37 | 6.20E-7 | 2.69E-6 | > 3.33E-5 |
| DU-145 | 0.439 | 2.029 | 2.079 | 2.144 | 2.060 | 0.076 | 0.080 | 103 | 107 | 102 | -83 | -82 | 6.37E-7 | 1.19E-6 | 2.22E-6 |
| Breast Cancer | | | | | | | | | | | | | | | |
| MCF7 | 0.368 | 2.366 | 2.179 | 2.278 | 2.078 | 0.145 | 0.155 | 91 | 96 | 86 | -61 | -58 | 5.83E-7 | 1.28E-6 | 2.82E-6 |
| MDA-MB-231/ATCC | 0.661 | 1.705 | 1.671 | 1.595 | 1.431 | 0.612 | 0.533 | 97 | 89 | 74 | -7 | -19 | 6.52E-7 | 2.69E-6 | > 3.33E-5 |
| HS 578T | 1.398 | 2.514 | 2.391 | 2.400 | 2.450 | 1.048 | 1.150 | 89 | 90 | 94 | -25 | -18 | 7.82E-7 | 2.05E-6 | > 3.33E-5 |
| BT-549 | 1.363 | 2.471 | 2.428 | 2.403 | 2.311 | 0.049 | 0.074 | 96 | 94 | 86 | -96 | -95 | 5.22E-7 | 9.83E-7 | 1.85E-6 |
| T-47D | 1.004 | 2.375 | 2.232 | 2.245 | 2.115 | 0.849 | 0.514 | 90 | 90 | 81 | -15 | -49 | 6.98E-7 | 2.30E-6 | > 3.33E-5 |
| MDA-MB-468 | 0.883 | 2.186 | 2.175 | 2.184 | 2.089 | 0.662 | 0.479 | 99 | 100 | 93 | -25 | -46 | 7.66E-7 | 2.04E-6 | > 3.33E-5 |

**Figure S37. Log 10 concentration of compound 3d**

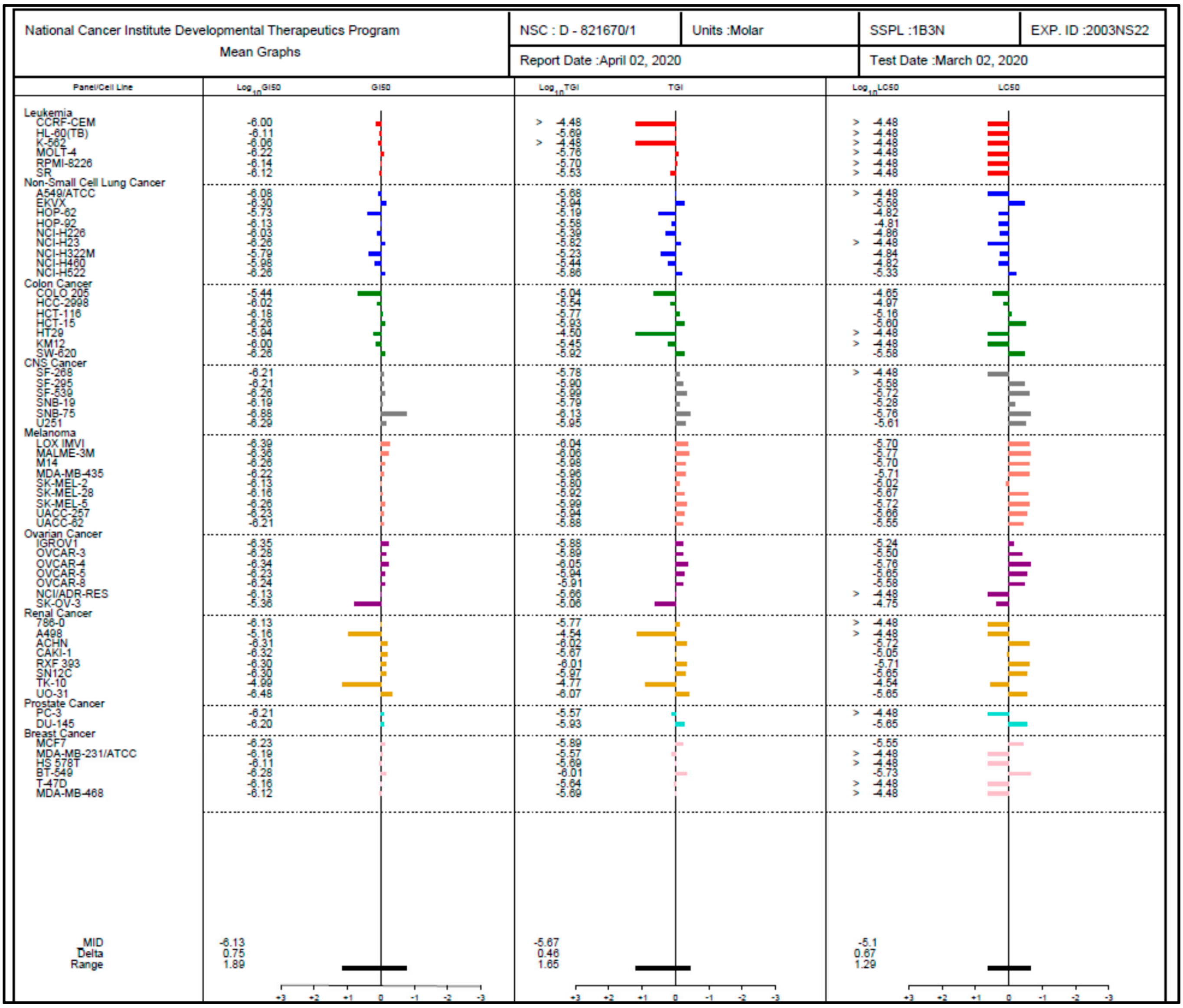


**Figure S38. Log 10 concentration of compound 3d**

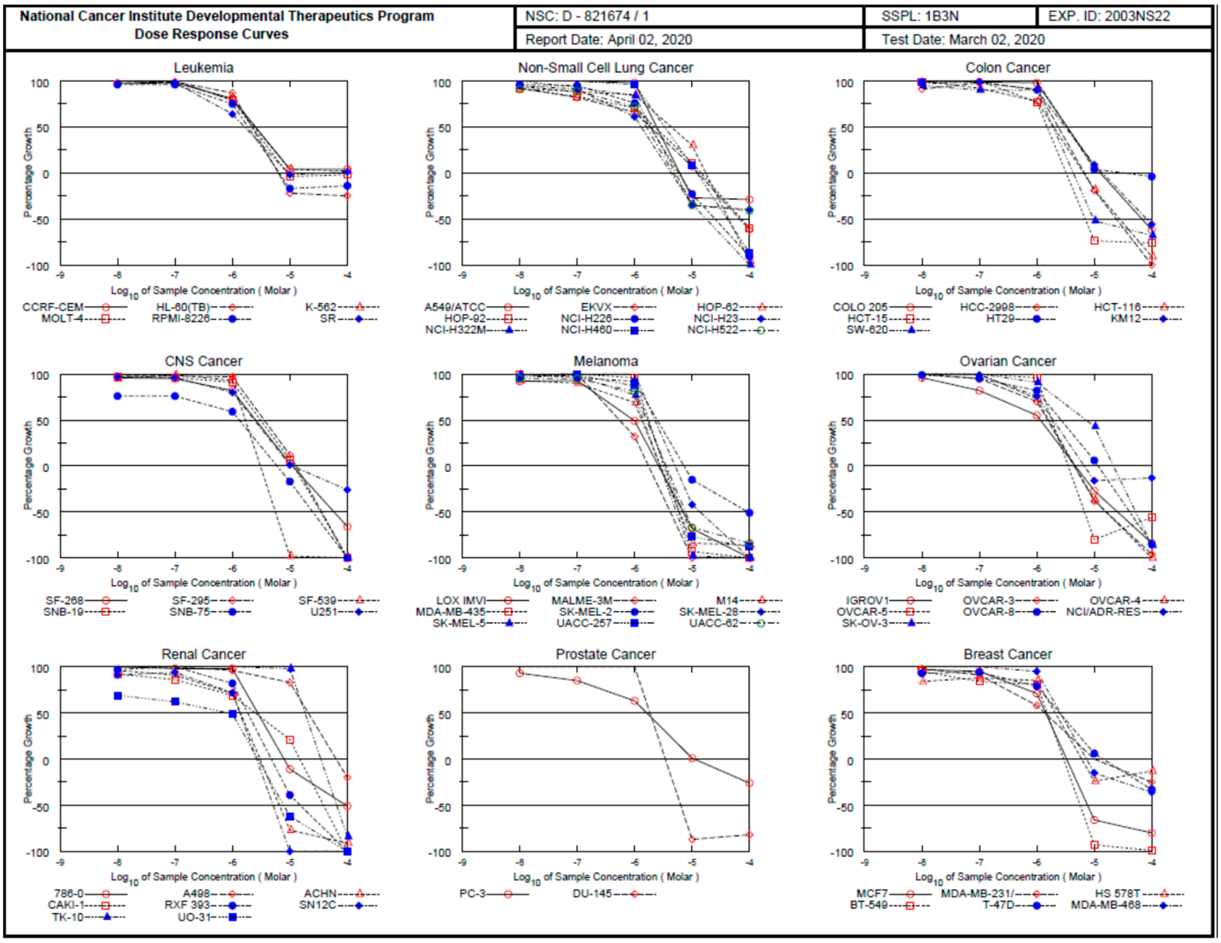


**Figure S39. Dose Response Curves for all cell line for compound 3e**

## National Cancer Institute Developmental Therapeutics Program
## In-Vitro Testing Results

| NSC : D - 821674 / 1 | Experiment ID : 2003NS22 | Test Type : 08 | Units : Molar |
|---|---|---|---|
| Report Date : April 02, 2020 | Test Date : March 02, 2020 | QNS : | MC : |
| COMI : LE 146 | Stain Reagent : SRB Dual-Pass Related | SSPL : 1B3N | |

| | | | Log10 Concentration | | | | | | | | | | | | | |
|---|---|---|---|---|---|---|---|---|---|---|---|---|---|---|---|---|
| | Time | | Mean Optical Densities | | | | | Percent Growth | | | | | | | | |
| Panel/Cell Line | Zero | Ctrl | -8.0 | -7.0 | -6.0 | -5.0 | -4.0 | -8.0 | -7.0 | -6.0 | -5.0 | -4.0 | GI50 | TGI | LC50 |
| Leukemia | | | | | | | | | | | | | | | |
| CCRF-CEM | 0.510 | 2.691 | 2.630 | 2.639 | 2.283 | 0.590 | 0.602 | 97 | 98 | 81 | 4 | 4 | 2.53E-6 | > 1.00E-4 | > 1.00E-4 |
| HL-60(TB) | 0.825 | 3.183 | 3.125 | 3.148 | 2.871 | 0.642 | 0.618 | 98 | 99 | 87 | -22 | -25 | 2.18E-6 | 6.26E-6 | > 1.00E-4 |
| K-562 | 0.235 | 2.184 | 2.183 | 2.210 | 1.773 | 0.315 | 0.273 | 100 | 101 | 79 | 4 | 2 | 2.43E-6 | > 1.00E-4 | > 1.00E-4 |
| MOLT-4 | 0.763 | 3.137 | 3.161 | 3.175 | 2.632 | 0.731 | 0.749 | 101 | 102 | 79 | -4 | -2 | 2.22E-6 | 8.89E-6 | > 1.00E-4 |
| RPMI-8226 | 0.711 | 2.827 | 2.751 | 2.749 | 2.295 | 0.587 | 0.610 | 96 | 96 | 75 | -17 | -14 | 1.86E-6 | 6.47E-6 | > 1.00E-4 |
| SR | 0.391 | 1.715 | 1.751 | 1.686 | 1.241 | 0.383 | 0.405 | 103 | 98 | 64 | -2 | 1 | 1.64E-6 | . | > 1.00E-4 |
| Non-Small Cell Lung Cancer | | | | | | | | | | | | | | | |
| A549/ATCC | 0.425 | 2.366 | 2.391 | 2.502 | 2.329 | 0.310 | 0.303 | 101 | 107 | 98 | -27 | -29 | 2.42E-6 | 6.07E-6 | > 1.00E-4 |
| EKVX | 0.561 | 2.124 | 1.997 | 1.837 | 1.596 | 0.664 | 0.211 | 92 | 82 | 66 | 7 | -62 | 1.87E-6 | 1.25E-5 | 6.60E-5 |
| HOP-62 | 1.398 | 3.018 | 2.921 | 2.826 | 2.776 | 1.877 | 0.052 | 94 | 88 | 85 | 30 | -96 | 4.28E-6 | 1.72E-5 | 4.29E-5 |
| HOP-92 | 1.117 | 1.906 | 1.841 | 1.775 | 1.680 | 1.206 | 0.449 | 92 | 83 | 71 | 11 | -60 | 2.26E-6 | 1.44E-5 | 7.28E-5 |
| NCI-H226 | 0.785 | 2.010 | 1.955 | 1.946 | 1.710 | 0.604 | 0.074 | 96 | 95 | 76 | -23 | -91 | 1.81E-6 | 5.83E-6 | 2.50E-5 |
| NCI-H23 | 0.897 | 2.667 | 2.579 | 2.508 | 1.981 | 0.583 | 0.540 | 95 | 91 | 61 | -35 | -40 | 1.31E-6 | 4.32E-6 | > 1.00E-4 |
| NCI-H322M | 0.676 | 1.848 | 1.854 | 1.771 | 1.655 | 0.445 | -0.036 | 101 | 93 | 84 | -34 | -100 | 1.93E-6 | 5.13E-6 | 1.74E-5 |
| NCI-H460 | 0.317 | 2.879 | 2.947 | 2.919 | 2.775 | 0.533 | 0.041 | 103 | 102 | 96 | 8 | -87 | 3.35E-6 | 1.22E-5 | 4.09E-5 |
| NCI-H522 | 0.874 | 2.268 | 2.141 | 2.122 | 1.874 | 0.567 | 0.515 | 91 | 89 | 72 | -35 | -41 | 1.60E-6 | 4.69E-6 | > 1.00E-4 |
| Colon Cancer | | | | | | | | | | | | | | | |
| COLO 205 | 0.722 | 2.950 | 3.030 | 2.931 | 2.898 | 0.882 | 0.271 | 104 | 99 | 98 | 7 | -62 | 3.36E-6 | 1.27E-5 | 6.62E-5 |
| HCC-2998 | 0.774 | 2.528 | 2.373 | 2.489 | 2.364 | 0.625 | -0.018 | 91 | 98 | 91 | -19 | -100 | 2.34E-6 | 6.67E-6 | 2.40E-5 |
| HCT-116 | 0.304 | 2.858 | 2.862 | 2.658 | 2.286 | 0.249 | 0.027 | 100 | 92 | 78 | -18 | -91 | 1.94E-6 | 6.45E-6 | 2.73E-5 |
| HCT-15 | 0.260 | 2.347 | 2.327 | 2.380 | 1.865 | 0.069 | 0.062 | 99 | 102 | 77 | -74 | -76 | 1.51E-6 | 3.24E-6 | 6.96E-6 |
| HT29 | 0.396 | 2.555 | 2.534 | 2.646 | 2.560 | 0.477 | 0.379 | 99 | 104 | 100 | 4 | -4 | 3.32E-6 | 2.87E-5 | > 1.00E-4 |
| KM12 | 0.643 | 3.093 | 3.023 | 3.070 | 2.856 | 0.857 | 0.286 | 97 | 99 | 90 | 9 | -56 | 3.12E-6 | 1.37E-5 | 8.18E-5 |
| SW-620 | 0.360 | 2.619 | 2.511 | 2.403 | 2.397 | 0.174 | 0.116 | 95 | 90 | 90 | -52 | -68 | 1.92E-6 | 4.32E-6 | 9.71E-6 |
| CNS Cancer | | | | | | | | | | | | | | | |
| SF-268 | 0.756 | 2.512 | 2.449 | 2.422 | 2.194 | 0.808 | 0.260 | 96 | 95 | 82 | 3 | -66 | 2.54E-6 | 1.10E-5 | 5.91E-5 |
| SF-295 | 0.953 | 3.260 | 3.215 | 3.218 | 3.186 | 1.225 | -0.043 | 98 | 98 | 97 | 12 | -100 | 3.55E-6 | 1.27E-5 | 3.57E-5 |
| SF-539 | 0.702 | 2.407 | 2.330 | 2.390 | 2.284 | 0.012 | 0.001 | 95 | 99 | 93 | -98 | -100 | 1.67E-6 | 3.06E-6 | 5.58E-6 |
| SNB-19 | 0.901 | 2.515 | 2.470 | 2.451 | 2.370 | 1.021 | -0.063 | 97 | 96 | 91 | 7 | -100 | 3.09E-6 | 1.17E-5 | 3.42E-5 |
| SNB-75 | 1.913 | 2.863 | 2.631 | 2.632 | 2.476 | 1.596 | -0.020 | 76 | 76 | 59 | -17 | -100 | 1.32E-6 | 6.04E-6 | 2.51E-5 |
| U251 | 0.417 | 2.108 | 2.064 | 2.037 | 1.772 | 0.438 | 0.308 | 97 | 96 | 80 | 1 | -26 | 2.41E-6 | 1.11E-5 | > 1.00E-4 |
| Melanoma | | | | | | | | | | | | | | | |
| LOX IMVI | 0.342 | 2.434 | 2.259 | 2.297 | 1.374 | 0.108 | -0.037 | 92 | 93 | 49 | -68 | -100 | 9.66E-7 | 2.62E-6 | 6.98E-6 |
| MALME-3M | 0.733 | 1.338 | 1.331 | 1.369 | 0.928 | -0.012 | -0.057 | 99 | 105 | 32 | -100 | -100 | 5.70E-7 | 1.75E-6 | 4.19E-6 |
| M14 | 0.498 | 2.275 | 2.142 | 2.095 | 1.724 | 0.082 | 0.071 | 93 | 90 | 69 | -84 | -86 | 1.33E-6 | 2.83E-6 | 6.03E-6 |
| MDA-MB-435 | 0.572 | 2.578 | 2.566 | 2.656 | 2.508 | 0.041 | -0.045 | 99 | 104 | 96 | -93 | -100 | 1.76E-6 | 3.23E-6 | 5.93E-6 |
| SK-MEL-2 | 1.118 | 2.690 | 2.693 | 2.624 | 2.564 | 0.951 | 0.546 | 100 | 96 | 92 | -15 | -51 | 2.47E-6 | 7.25E-6 | 9.29E-5 |
| SK-MEL-28 | 0.802 | 2.155 | 2.218 | 2.299 | 2.237 | 0.468 | -0.024 | 105 | 111 | 106 | -42 | -100 | 2.40E-6 | 5.22E-6 | 1.39E-5 |
| SK-MEL-5 | 0.878 | 3.159 | 3.136 | 3.110 | 2.640 | 0.018 | -0.046 | 99 | 98 | 77 | -98 | -100 | 1.43E-6 | 2.76E-6 | 5.32E-6 |
| UACC-257 | 1.032 | 2.529 | 2.463 | 2.514 | 2.338 | 0.242 | 0.128 | 96 | 99 | 87 | -77 | -88 | 1.69E-6 | 3.41E-6 | 6.88E-6 |
| UACC-62 | 1.131 | 3.034 | 2.942 | 2.942 | 2.675 | 0.371 | 0.186 | 95 | 95 | 81 | -67 | -84 | 1.62E-6 | 3.52E-6 | 7.66E-6 |
| Ovarian Cancer | | | | | | | | | | | | | | | |
| IGROV1 | 0.483 | 2.061 | 1.991 | 1.780 | 1.355 | 0.355 | 0.079 | 96 | 82 | 55 | -27 | -84 | 1.16E-6 | 4.74E-6 | 2.58E-5 |
| OVCAR-3 | 0.492 | 1.683 | 1.682 | 1.622 | 1.325 | 0.303 | 0.022 | 100 | 95 | 70 | -38 | -96 | 1.53E-6 | 4.42E-6 | 1.59E-5 |
| OVCAR-4 | 1.065 | 1.968 | 2.027 | 1.965 | 1.723 | 0.666 | -0.044 | 107 | 100 | 73 | -37 | -100 | 1.61E-6 | 4.58E-6 | 1.59E-5 |
| OVCAR-5 | 0.797 | 2.042 | 2.041 | 2.070 | 1.996 | 0.163 | 0.352 | 100 | 102 | 96 | -80 | -56 | 1.83E-6 | 3.53E-6 | 6.79E-6 |
| OVCAR-8 | 0.451 | 2.338 | 2.311 | 2.237 | 1.994 | 0.562 | 0.066 | 99 | 95 | 82 | 6 | -85 | 2.62E-6 | 1.16E-5 | 4.10E-5 |
| NCI/ADR-RES | 0.567 | 2.195 | 2.204 | 2.182 | 1.806 | 0.479 | 0.495 | 101 | 99 | 76 | -16 | -13 | 1.93E-6 | 6.77E-6 | > 1.00E-4 |
| SK-OV-3 | 1.041 | 2.127 | 2.147 | 2.124 | 2.029 | 1.504 | 0.143 | 102 | 100 | 91 | 43 | -86 | 7.03E-6 | 2.14E-5 | 5.23E-5 |
| Renal Cancer | | | | | | | | | | | | | | | |
| 786-0 | 1.016 | 3.147 | 3.143 | 3.104 | 3.097 | 0.905 | 0.501 | 100 | 98 | 98 | -11 | -51 | 2.75E-6 | 7.93E-6 | 9.61E-5 |
| A498 | 1.819 | 2.582 | 2.581 | 2.617 | 2.548 | 2.449 | 1.448 | 100 | 105 | 96 | 83 | -20 | 2.07E-5 | 6.34E-5 | > 1.00E-4 |
| ACHN | 0.368 | 1.829 | 1.771 | 1.699 | 1.407 | 0.086 | 0.034 | 96 | 91 | 71 | -77 | -91 | 1.39E-6 | 3.03E-6 | 6.60E-6 |
| CAKI-1 | 0.530 | 1.953 | 1.843 | 1.753 | 1.509 | 0.831 | -0.019 | 92 | 86 | 69 | 21 | -100 | 2.48E-6 | 1.49E-5 | 3.87E-5 |
| RXF 393 | 0.962 | 1.743 | 1.716 | 1.754 | 1.606 | 0.584 | -0.029 | 97 | 101 | 82 | -39 | -100 | 1.85E-6 | 4.75E-6 | 1.50E-5 |
| SN12C | 0.763 | 2.699 | 2.521 | 2.585 | 2.148 | -0.005 | -0.051 | 91 | 94 | 72 | -100 | -100 | 1.33E-6 | 2.61E-6 | 5.11E-6 |
| TK-10 | 1.162 | 2.014 | 2.083 | 2.425 | 2.647 | 1.998 | 0.192 | 108 | 148 | 174 | 98 | -84 | 1.84E-5 | 3.47E-5 | 6.54E-5 |
| UO-31 | 0.618 | 1.756 | 1.409 | 1.326 | 1.177 | 0.237 | -0.059 | 69 | 62 | 49 | -62 | -100 | 8.50E-7 | 2.77E-6 | 7.85E-6 |
| Prostate Cancer | | | | | | | | | | | | | | | |
| PC-3 | 0.560 | 1.756 | 1.672 | 1.572 | 1.313 | 0.569 | 0.412 | 93 | 85 | 63 | 1 | -26 | 1.62E-6 | 1.07E-5 | > 1.00E-4 |
| DU-145 | 0.439 | 1.975 | 2.057 | 2.032 | 1.977 | 0.058 | 0.077 | 105 | 104 | 100 | -87 | -82 | 1.85E-6 | 3.43E-6 | 6.36E-6 |
| Breast Cancer | | | | | | | | | | | | | | | |
| MCF7 | 0.368 | 2.302 | 2.246 | 2.204 | 1.737 | 0.125 | 0.073 | 97 | 95 | 71 | -66 | -80 | 1.42E-6 | 3.29E-6 | 7.62E-6 |
| MDA-MB-231/ATCC | 0.661 | 1.660 | 1.639 | 1.568 | 1.241 | 0.658 | 0.497 | 98 | 91 | 58 | . | -25 | 1.37E-6 | 9.82E-6 | > 1.00E-4 |
| HS 578T | 1.398 | 2.565 | 2.379 | 2.430 | 2.386 | 1.065 | 1.210 | 84 | 88 | 85 | -24 | -13 | 2.09E-6 | 6.03E-6 | > 1.00E-4 |
| BT-549 | 1.363 | 2.576 | 2.515 | 2.385 | 2.342 | 0.092 | 0.015 | 95 | 84 | 81 | -93 | -99 | 1.50E-6 | 2.91E-6 | 5.64E-6 |
| T-47D | 1.004 | 2.401 | 2.306 | 2.317 | 2.105 | 1.090 | 0.672 | 93 | 94 | 79 | 6 | -33 | 2.49E-6 | 1.44E-5 | > 1.00E-4 |
| MDA-MB-468 | 0.883 | 2.247 | 2.273 | 2.288 | 2.173 | 0.752 | 0.569 | 102 | 103 | 95 | -15 | -36 | 2.55E-6 | 7.32E-6 | > 1.00E-4 |

**Figure S40. Log 10 concentration of compound 3e**

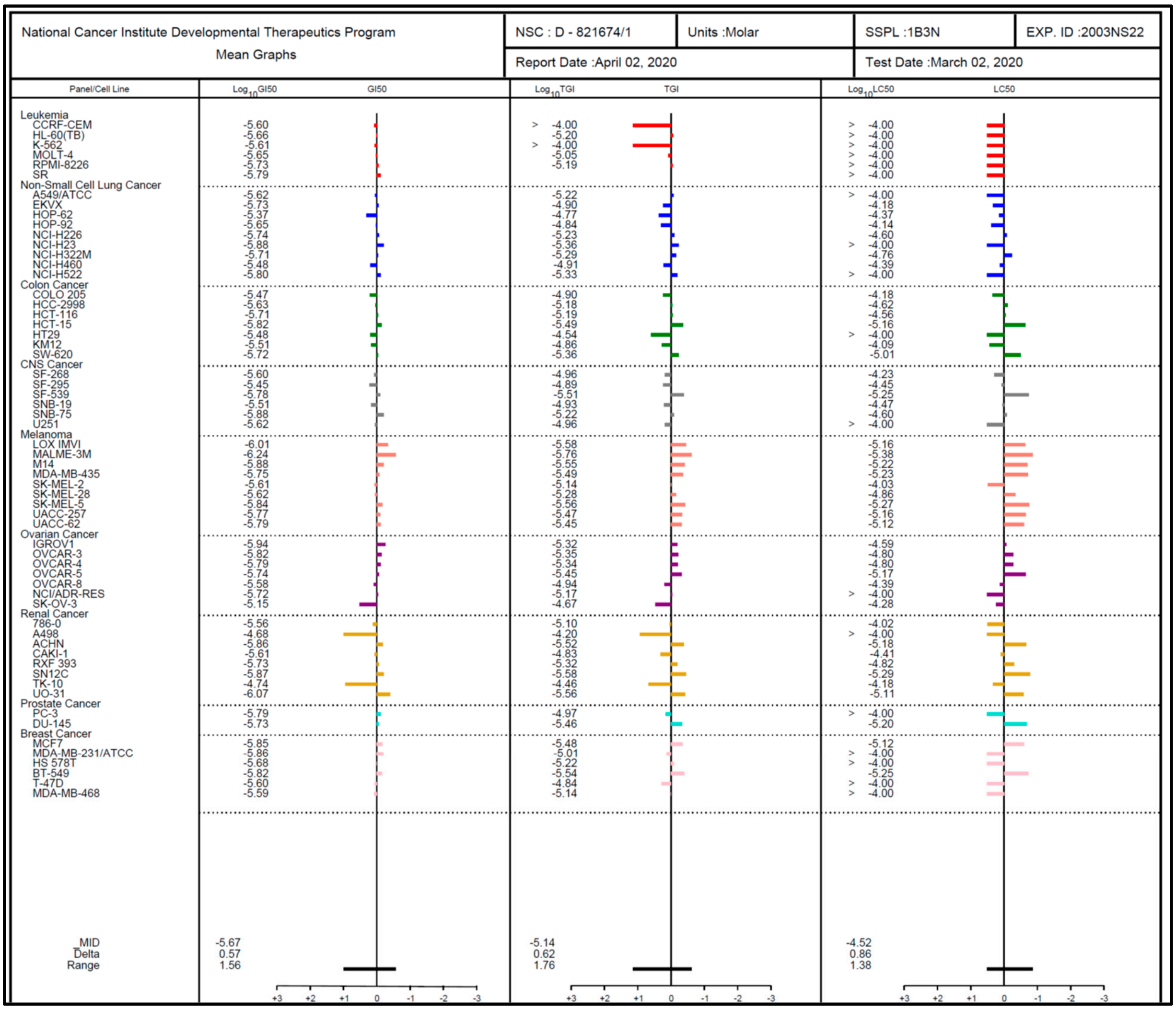


**Figure S41. Log 10 concentration of compound 3e**

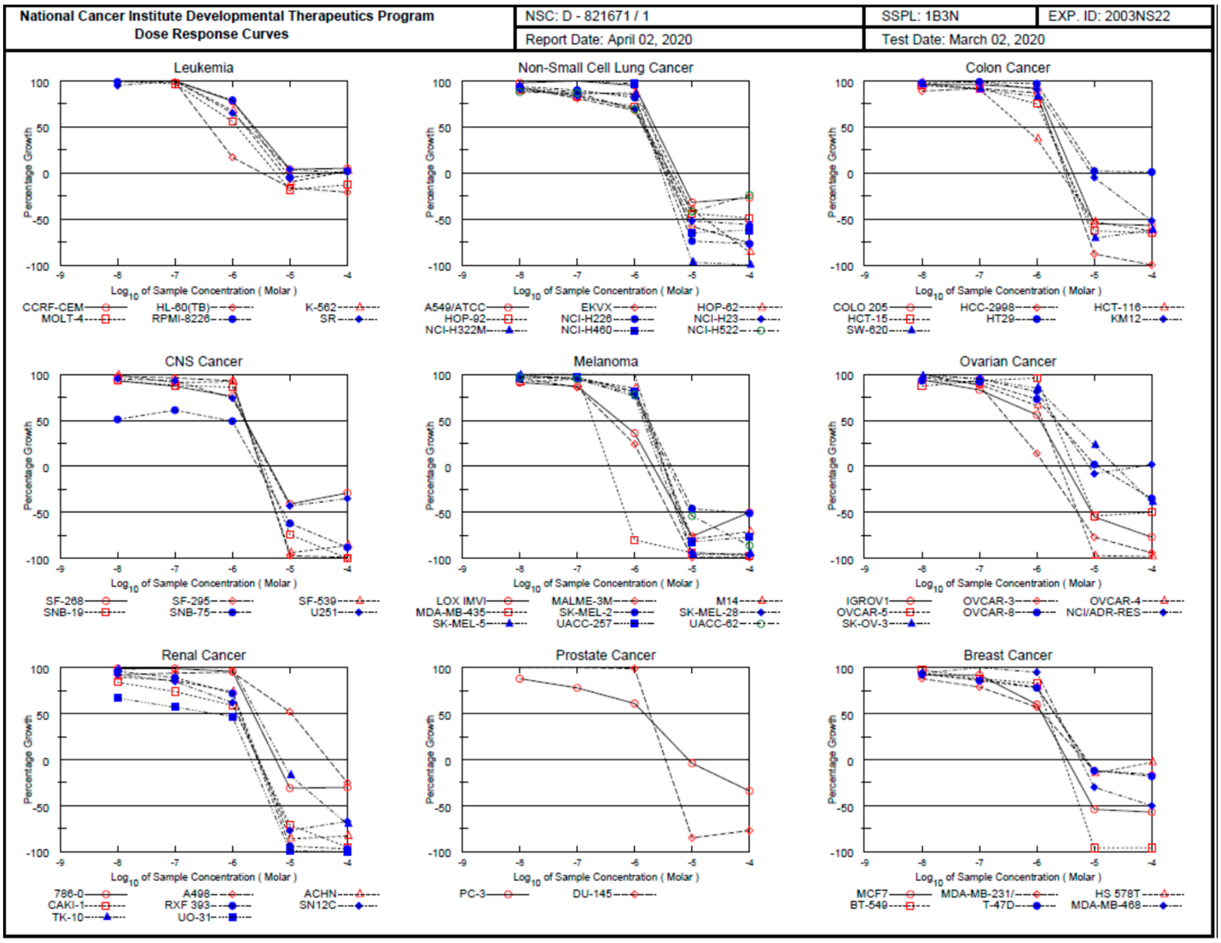


**Figure S42. Dose Response Curves for all cell line for compound 3c**

# National Cancer Institute Developmental Therapeutics Program
## In-Vitro Testing Results

| | | | |
|---|---|---|---|
| NSC : D - 821671 / 1 | Experiment ID : 2003NS22 | Test Type : 08 | Units : Molar |
| Report Date : April 02, 2020 | Test Date : March 02, 2020 | QNS : | MC : |
| COMI : LE 155 | Stain Reagent : SRB Dual-Pass Related | SSPL : 1B3N | |

| | Time | | Log10 Concentration | | | | | | | | | | | | |
|---|---|---|---|---|---|---|---|---|---|---|---|---|---|---|---|
| | | | Mean Optical Densities | | | | | Percent Growth | | | | | | | |
| Panel/Cell Line | Zero | Ctrl | -8.0 | -7.0 | -6.0 | -5.0 | -4.0 | -8.0 | -7.0 | -6.0 | -5.0 | -4.0 | GI50 | TGI | LC50 |
| Leukemia | | | | | | | | | | | | | | | |
| CCRF-CEM | 0.510 | 2.658 | 2.693 | 2.672 | 2.191 | 0.587 | 0.623 | 102 | 101 | 78 | 4 | 5 | 2.39E-6 | > 1.00E-4 | > 1.00E-4 |
| HL-60(TB) | 0.825 | 3.183 | 3.200 | 3.161 | 1.224 | 0.693 | 0.654 | 101 | 99 | 17 | -16 | -21 | 3.95E-7 | 3.26E-6 | > 1.00E-4 |
| K-562 | 0.235 | 2.239 | 2.266 | 2.311 | 1.593 | 0.211 | 0.273 | 101 | 104 | 68 | -10 | 2 | 1.69E-6 | . | > 1.00E-4 |
| MOLT-4 | 0.763 | 3.141 | 3.136 | 3.080 | 2.106 | 0.627 | 0.668 | 100 | 97 | 56 | -18 | -13 | 1.22E-6 | 5.76E-6 | > 1.00E-4 |
| RPMI-8226 | 0.711 | 2.867 | 2.845 | 2.893 | 2.410 | 0.676 | 0.763 | 99 | 101 | 79 | -5 | 2 | 2.21E-6 | . | > 1.00E-4 |
| SR | 0.391 | 1.744 | 1.672 | 1.749 | 1.275 | 0.451 | 0.396 | 95 | 100 | 65 | 4 | . | 1.78E-6 | > 1.00E-4 | > 1.00E-4 |
| Non-Small Cell Lung Cancer | | | | | | | | | | | | | | | |
| A549/ATCC | 0.425 | 2.293 | 2.253 | 2.359 | 2.198 | 0.290 | 0.310 | 98 | 104 | 95 | -32 | -27 | 2.26E-6 | 5.61E-6 | > 1.00E-4 |
| EKVX | 0.561 | 2.078 | 1.966 | 1.791 | 1.596 | 0.234 | 0.135 | 93 | 81 | 68 | -58 | -76 | 1.39E-6 | 3.46E-6 | 8.60E-6 |
| HOP-62 | 1.398 | 2.939 | 2.773 | 2.718 | 2.742 | 0.840 | 0.193 | 89 | 86 | 87 | -40 | -86 | 1.96E-6 | 4.85E-6 | 1.65E-5 |
| HOP-92 | 1.117 | 1.845 | 1.793 | 1.720 | 1.645 | 0.625 | 0.574 | 93 | 83 | 72 | -44 | -49 | 1.56E-6 | 4.19E-6 | > 1.00E-4 |
| NCI-H226 | 0.785 | 2.033 | 1.955 | 1.903 | 1.812 | 0.202 | 0.177 | 94 | 90 | 82 | -74 | -77 | 1.61E-6 | 3.35E-6 | 6.99E-6 |
| NCI-H23 | 0.897 | 2.486 | 2.350 | 2.230 | 2.007 | 0.435 | 0.392 | 91 | 84 | 70 | -52 | -56 | 1.46E-6 | 3.76E-6 | 9.72E-6 |
| NCI-H322M | 0.676 | 1.772 | 1.686 | 1.637 | 1.602 | 0.019 | -0.002 | 92 | 88 | 85 | -97 | -100 | 1.55E-6 | 2.92E-6 | 5.50E-6 |
| NCI-H460 | 0.317 | 2.930 | 2.968 | 2.996 | 2.840 | 0.112 | 0.121 | 101 | 103 | 97 | -65 | -62 | 1.94E-6 | 3.97E-6 | 8.11E-6 |
| NCI-H522 | 0.874 | 2.183 | 2.023 | 2.018 | 1.778 | 0.508 | 0.666 | 88 | 87 | 69 | -42 | -24 | 1.49E-6 | 4.19E-6 | > 1.00E-4 |
| Colon Cancer | | | | | | | | | | | | | | | |
| COLO 205 | 0.722 | 2.900 | 2.815 | 2.819 | 2.732 | 0.327 | 0.311 | 96 | 96 | 92 | -55 | -57 | 1.94E-6 | 4.24E-6 | 9.28E-6 |
| HCC-2998 | 0.774 | 2.533 | 2.347 | 2.385 | 2.299 | 0.093 | -0.001 | 89 | 92 | 87 | -88 | -100 | 1.62E-6 | 3.14E-6 | 6.06E-6 |
| HCT-116 | 0.304 | 2.842 | 2.780 | 2.624 | 1.244 | 0.144 | 0.114 | 98 | 91 | 37 | -53 | -63 | 5.77E-7 | 2.58E-6 | 9.31E-6 |
| HCT-15 | 0.260 | 2.399 | 2.292 | 2.235 | 1.868 | 0.097 | 0.090 | 95 | 92 | 75 | -63 | -65 | 1.52E-6 | 3.50E-6 | 8.07E-6 |
| HT29 | 0.396 | 2.543 | 2.491 | 2.518 | 2.468 | 0.430 | 0.412 | 98 | 99 | 97 | 2 | 1 | 3.09E-6 | > 1.00E-4 | > 1.00E-4 |
| KM12 | 0.643 | 3.135 | 3.139 | 3.108 | 2.922 | 0.609 | 0.309 | 100 | 99 | 91 | -5 | -52 | 2.68E-6 | 8.80E-6 | 9.05E-5 |
| SW-620 | 0.360 | 2.637 | 2.540 | 2.422 | 2.243 | 0.105 | 0.138 | 96 | 91 | 83 | -71 | -62 | 1.63E-6 | 3.45E-6 | 7.30E-6 |
| CNS Cancer | | | | | | | | | | | | | | | |
| SF-268 | 0.756 | 2.524 | 2.400 | 2.302 | 2.107 | 0.446 | 0.533 | 93 | 87 | 76 | -41 | -29 | 1.68E-6 | 4.48E-6 | > 1.00E-4 |
| SF-295 | 0.953 | 3.229 | 3.190 | 3.141 | 3.060 | 0.031 | 0.012 | 98 | 96 | 93 | -97 | -99 | 1.68E-6 | 3.08E-6 | 5.66E-6 |
| SF-539 | 0.702 | 2.401 | 2.390 | 2.249 | 2.270 | 0.041 | 0.099 | 99 | 91 | 92 | -94 | -86 | 1.69E-6 | 3.12E-6 | 5.79E-6 |
| SNB-19 | 0.901 | 2.530 | 2.412 | 2.338 | 2.299 | 0.232 | -0.014 | 93 | 88 | 86 | -74 | -100 | 1.67E-6 | 3.44E-6 | 7.05E-6 |
| SNB-75 | 1.913 | 2.894 | 2.412 | 2.513 | 2.395 | 0.726 | 0.232 | 51 | 61 | 49 | -62 | -88 | 8.43E-7 | 2.76E-6 | 7.79E-6 |
| U251 | 0.417 | 2.043 | 1.954 | 1.921 | 1.616 | 0.238 | 0.272 | 95 | 93 | 74 | -43 | -35 | 1.60E-6 | 4.28E-6 | > 1.00E-4 |
| Melanoma | | | | | | | | | | | | | | | |
| LOX IMVI | 0.342 | 2.445 | 2.262 | 2.165 | 1.102 | 0.082 | 0.171 | 91 | 87 | 36 | -76 | -50 | 5.32E-7 | 2.10E-6 | 5.86E-6 |
| MALME-3M | 0.733 | 1.265 | 1.243 | 1.189 | 0.863 | 0.006 | 0.008 | 96 | 86 | 24 | -99 | -99 | 3.83E-7 | 1.58E-6 | 4.00E-6 |
| M14 | 0.498 | 2.251 | 2.097 | 2.165 | 1.989 | 0.104 | 0.142 | 91 | 95 | 85 | -79 | -71 | 1.63E-6 | 3.30E-6 | 6.65E-6 |
| MDA-MB-435 | 0.572 | 2.542 | 2.535 | 2.592 | 0.112 | 0.034 | 0.015 | 100 | 103 | -80 | -94 | -97 | 1.94E-7 | 3.63E-7 | 6.82E-7 |
| SK-MEL-2 | 1.118 | 2.596 | 2.574 | 2.537 | 2.311 | 0.600 | 0.553 | 98 | 96 | 81 | -46 | -51 | 1.74E-6 | 4.32E-6 | 7.27E-5 |
| SK-MEL-28 | 0.802 | 2.118 | 2.161 | 2.205 | 2.171 | 0.049 | 0.029 | 103 | 107 | 104 | -94 | -96 | 1.87E-6 | 3.35E-6 | 6.00E-6 |
| SK-MEL-5 | 0.878 | 3.175 | 3.157 | 3.055 | 2.620 | 0.031 | 0.045 | 99 | 95 | 76 | -96 | -95 | 1.41E-6 | 2.75E-6 | 5.37E-6 |
| UACC-257 | 1.032 | 2.464 | 2.403 | 2.420 | 2.191 | 0.190 | 0.236 | 96 | 97 | 81 | -82 | -77 | 1.55E-6 | 3.15E-6 | 6.39E-6 |
| UACC-62 | 1.131 | 2.967 | 2.918 | 2.869 | 2.560 | 0.525 | 0.157 | 97 | 95 | 78 | -54 | -86 | 1.63E-6 | 3.91E-6 | 9.38E-6 |
| Ovarian Cancer | | | | | | | | | | | | | | | |
| IGROV1 | 0.483 | 1.984 | 1.898 | 1.725 | 1.324 | 0.219 | 0.112 | 94 | 83 | 56 | -55 | -77 | 1.13E-6 | 3.21E-6 | 9.08E-6 |
| OVCAR-3 | 0.492 | 1.714 | 1.728 | 1.571 | 0.664 | 0.112 | 0.029 | 101 | 88 | 14 | -77 | -94 | 3.28E-7 | 1.43E-6 | 5.03E-6 |
| OVCAR-4 | 1.065 | 1.998 | 1.968 | 1.896 | 1.685 | 0.036 | 0.021 | 97 | 89 | 66 | -97 | -98 | 1.26E-6 | 2.56E-6 | 5.18E-6 |
| OVCAR-5 | 0.797 | 2.041 | 1.878 | 1.950 | 1.994 | 0.368 | 0.398 | 87 | 93 | 96 | -54 | -50 | 2.03E-6 | 4.37E-6 | 9.42E-6 |
| OVCAR-8 | 0.451 | 2.377 | 2.242 | 2.226 | 1.866 | 0.486 | 0.294 | 93 | 92 | 73 | 2 | -35 | 2.13E-6 | 1.12E-5 | > 1.00E-4 |
| NCI/ADR-RES | 0.567 | 2.184 | 2.180 | 2.104 | 1.880 | 0.522 | 0.597 | 100 | 95 | 81 | -8 | 2 | 2.24E-6 | . | > 1.00E-4 |
| SK-OV-3 | 1.041 | 2.044 | 2.026 | 1.990 | 1.899 | 1.272 | 0.632 | 98 | 95 | 85 | 23 | -39 | 3.70E-6 | 2.34E-5 | > 1.00E-4 |
| Renal Cancer | | | | | | | | | | | | | | | |
| 786-0 | 1.016 | 3.077 | 3.047 | 3.067 | 2.993 | 0.701 | 0.711 | 99 | 99 | 96 | -31 | -30 | 2.30E-6 | 5.70E-6 | > 1.00E-4 |
| A498 | 1.819 | 2.610 | 2.548 | 2.561 | 2.574 | 2.229 | 1.360 | 92 | 94 | 95 | 52 | -25 | 1.05E-5 | 4.70E-5 | > 1.00E-4 |
| ACHN | 0.368 | 1.804 | 1.664 | 1.608 | 1.437 | 0.051 | 0.064 | 90 | 86 | 74 | -86 | -83 | 1.42E-6 | 2.91E-6 | 5.96E-6 |
| CAKI-1 | 0.530 | 1.911 | 1.693 | 1.549 | 1.351 | 0.155 | 0.025 | 84 | 74 | 59 | -71 | -95 | 1.18E-6 | 2.86E-6 | 6.92E-6 |
| RXF 393 | 0.962 | 1.803 | 1.770 | 1.708 | 1.567 | 0.058 | 0.026 | 96 | 89 | 72 | -94 | -97 | 1.35E-6 | 2.71E-6 | 5.43E-6 |
| SN12C | 0.763 | 2.720 | 2.573 | 2.434 | 1.971 | 0.178 | 0.253 | 93 | 85 | 62 | -77 | -67 | 1.22E-6 | 2.79E-6 | 6.42E-6 |
| TK-10 | 1.162 | 1.919 | 1.905 | 2.253 | 2.492 | 0.962 | 0.343 | 98 | 144 | 176 | -17 | -70 | 4.48E-6 | 8.14E-6 | 4.13E-5 |
| UO-31 | 0.618 | 1.662 | 1.320 | 1.214 | 1.108 | 0.004 | -0.010 | 67 | 57 | 47 | -99 | -100 | 4.96E-7 | 2.09E-6 | 4.60E-6 |
| Prostate Cancer | | | | | | | | | | | | | | | |
| PC-3 | 0.560 | 1.738 | 1.601 | 1.481 | 1.283 | 0.540 | 0.371 | 88 | 78 | 61 | -4 | -34 | 1.49E-6 | 8.81E-6 | > 1.00E-4 |
| DU-145 | 0.439 | 2.029 | 2.114 | 2.065 | 2.014 | 0.066 | 0.102 | 105 | 102 | 99 | -85 | -77 | 1.85E-6 | 3.45E-6 | 6.46E-6 |
| Breast Cancer | | | | | | | | | | | | | | | |
| MCF7 | 0.368 | 2.366 | 2.206 | 2.206 | 1.576 | 0.168 | 0.159 | 92 | 92 | 60 | -54 | -57 | 1.23E-6 | 3.36E-6 | 9.17E-6 |
| MDA-MB-231/ATCC | 0.661 | 1.705 | 1.580 | 1.485 | 1.257 | 0.581 | 0.558 | 88 | 79 | 57 | -12 | -16 | 1.27E-6 | 6.69E-6 | > 1.00E-4 |
| HS 578T | 1.398 | 2.514 | 2.447 | 2.367 | 2.282 | 1.189 | 1.362 | 94 | 87 | 79 | -15 | -3 | 2.04E-6 | 6.93E-6 | > 1.00E-4 |
| BT-549 | 1.363 | 2.471 | 2.453 | 2.337 | 2.281 | 0.058 | 0.054 | 98 | 88 | 83 | -96 | -96 | 1.53E-6 | 2.91E-6 | 5.54E-6 |
| T-47D | 1.004 | 2.375 | 2.272 | 2.188 | 2.067 | 0.880 | 0.821 | 93 | 86 | 78 | -12 | -18 | 2.03E-6 | 7.29E-6 | > 1.00E-4 |
| MDA-MB-468 | 0.883 | 2.186 | 2.108 | 2.189 | 2.120 | 0.615 | 0.443 | 94 | 100 | 95 | -30 | -50 | 2.28E-6 | 5.72E-6 | > 1.00E-4 |

**Figure S43. Log 10 concentration of compound 3c**

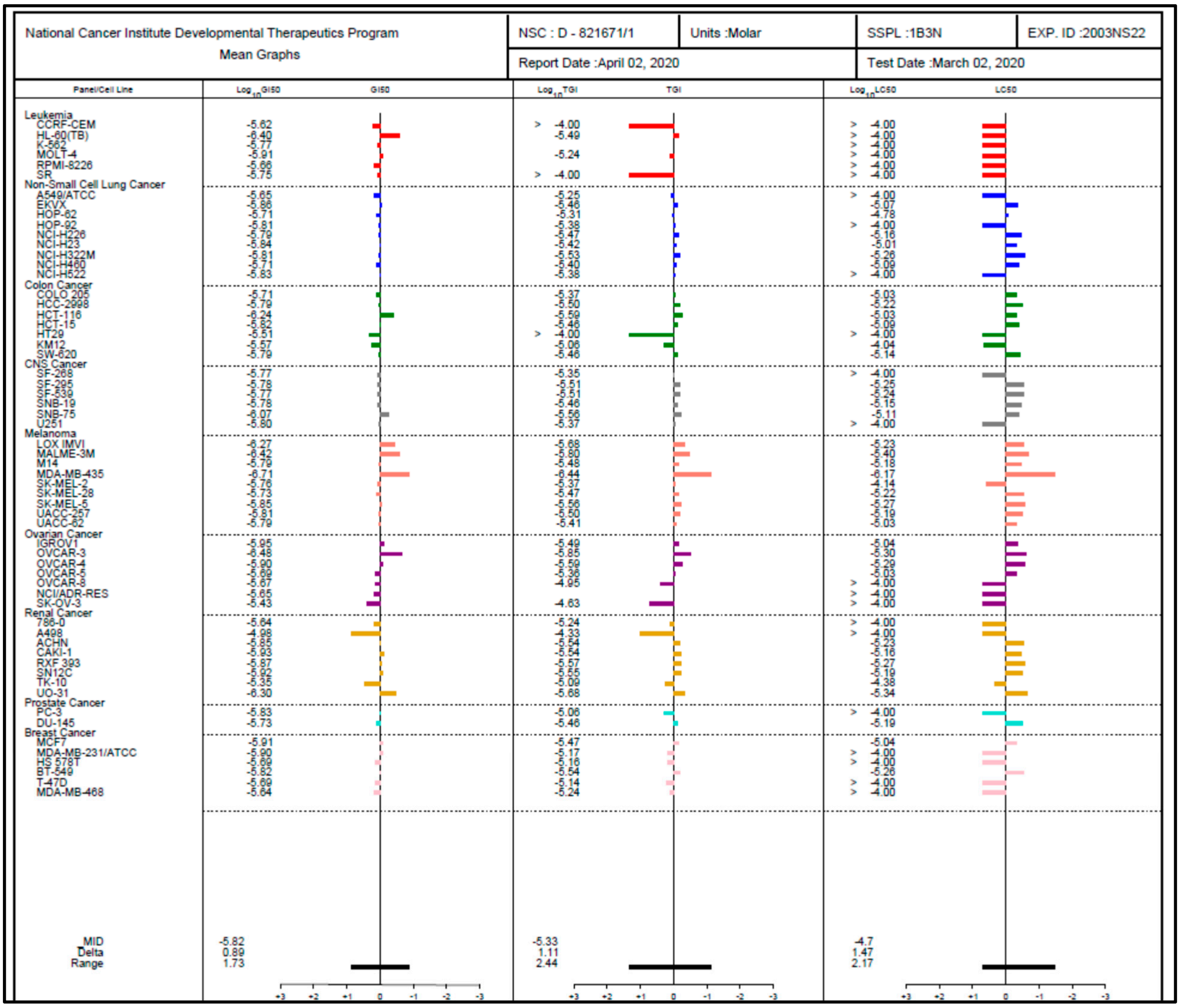


**Figure S44. Log 10 concentration of compound 3c**

# III- Material and methods

### *1. NCI screening assay*

As mentioned, the methodology of the NCI procedure for primary anticancer assay was detailed on their site (http://www.dtp.nci.nih.gov). But briefly, the protocol performed at sixty human tumor cell lines panel derived from different nine neoplastic diseases. NCI-60 testing is performed in two parts: first, a single concentration is tested in all 60 cell lines at a single dose of 10-5 molar or 15 µg/mL in accordance with the protocol of the Drug Evaluation Branch, National Cancer Institute, Bethesda, USA. If the results obtained meet selection criteria, then the compound is tested again in all 60 cell lines in 5 x 10 folds of dilution with the top dose being 10-4 molar or 150 µg/mL. Detailed methods are described in supplementary material related to this article.

### *2. MTT- Cytotoxicity assay method*

The MTT method of monitoring *in vitro* cytotoxicity is well suited for use with multi well plates. For best results, cells in the log phase of growth should be employed and final cell number should not exceed 106 cells/$cm^2$. Each test should include a blank containing complete medium without cells.

1. Remove cultures from incubator into laminar flow hood or other sterile work area.
2. Reconstitute each vial of MTT [M-5655] to be used with 3 ml of medium or balanced salt solution without phenol red and serum. Add reconstituted MTT in an amount equal to 10% of the culture medium volume.
3. Return cultures to incubator for 2-4 h depending on cell type and maximum cell density. (An incubation period of 2 h is generally adequate but may be lengthened for low cell densities or cells with lower metabolic activity.) Incubation times should be consistent when making comparisons.

4. After the incubation period, remove cultures from incubator and dissolve the resulting formazan crystals by adding an amount of MTT Solubilization Solution [M-8910] equal to the original culture medium volume.
5. Gentle mixing in a gyratory shaker will enhance dissolution. Occasionally, especially in dense cultures, pipetting up and down [trituration] may be required to completely dissolve the MTT formazan crystals.
6. Spectrophotometrically measure absorbance at a wavelength of 570 nm. Measure the background absorbance of multi-well plates at 690 nm and subtract from the 450 nm measurement. Tests performed in multi-well plates can be read using the appropriate type of plate reader or the contents of individual wells may be transferred to appropriate size cuvets for spectrophotometric measurement.

## 3. CDK inhibitory assay

### 3.1. Assay Protocol for CDK1/cyclinB

All samples and controls should be tested in duplicate.

1) Thaw 5x Kinase assay buffer 1, ATP and 10x CDK substrate peptide 1.

(Optional: If desired, add DTT to 5x Kinase assay buffer 1 to make a 10 mM concentration, e.g. add 10 µl of 1 M DTT to 1 ml 5x Kinase assay buffer 1)

2)Prepare the master mixture (25 µl per well): N wells x (6 µl 5x Kinase assay buffer 1 + 1 µl ATP (500 µM) + 5 µl 10x CDK substrate peptide 1 + 13 µl distilled water). Add 25 µl to every well.

3)Add 5 µl of Inhibitor solution of each well labeled as “Test Inhibitor”. For the “Positive Control" and “Blank”, add 5 µl of the same solution without inhibitor (Inhibitor buffer).

4) Prepare 3 ml of 1x Kinase assay buffer 1 by mixing 600 µl of 5x Kinase assay buffer 1 with 2400 µl water. 3 ml of 1x Kinase assay buffer 1 is sufficient for 100 reactions.

5) To the wells designated as "Blank", add 20 µl of 1x Kinase assay buffer 1.

6) Thaw CDK1/CyclinB1 enzyme on ice. Upon first thaw, briefly spin tube containing enzyme to recover full content of the tube. Calculate the amount of CDK1/CyclinB1 required for the assay and dilute enzyme to ~1.0 ng/µl with 1x Kinase assay buffer 1. Store remaining undiluted enzyme in aliquots at -80°C. Note: CDK1/CyclinB1 enzyme is sensitive to freeze/thaw cycles. Avoid multiple freeze/thaw cycles. Do not re-use thawed aliquots or diluted enzyme.

7) Initiate reaction by adding 20 µl of diluted CDK1/CyclinB1 enzyme to the wells designated “Positive Control” and "Test Inhibitor Control". Incubate at 30°C for 45 minutes.

8) Thaw Kinase-Glo Max reagent.

### 3.2. Assay Protocol for CDK2

All samples and controls should be tested in duplicate.

1) Thaw 5x Kinase assay buffer 1, ATP and 10x CDK substrate peptide 1. (Optional: If desired, add DTT to 5x Kinase assay buffer 1 to make a 10 mM concentration; e.g. add 10 µl of 1 M DTT to 1 ml 5x Kinase assay buffer 1)

2) Prepare the master mixture (25 µl per well): N wells x (6 µl 5x Kinase assay buffer 1 + 1 µl ATP (500 µM) + 5 µl 10x CDK substrate peptide 1 + 13 µl distilled water). Add 25 µl to every well.

3) Add 5 µl of Inhibitor solution of each well labeled as “Test Inhibitor”. For the “Positive Control" and “Blank”, add 5 µl of the same solution without inhibitor (Inhibitor buffer).

4) Prepare 3 ml of 1x Kinase assay buffer 1 by mixing 600 µl of 5x Kinase assay buffer 1 with 2400 µl water. 3 ml of 1x Kinase assay buffer 1 is sufficient for 100 reactions.

5) To the wells designated as "Blank", add 20 µl of 1x Kinase assay buffer 1.

6) Thaw CDK2/CyclinA2 enzyme on ice. Upon first thaw, briefly spin tube containing enzyme to recover full content of the tube. Calculate the amount of CDK2/CyclinA2 required for the assay and dilute enzyme to ~2.5 ng/µl with 1x Kinase assay buffer 1. Store remaining undiluted enzyme in aliquots at -80°C.

Note: CDK2/CyclinA2 enzyme is sensitive to freeze/thaw cycles. Avoid multiple freeze/thaw cycles. Do not re-use thawed aliquots or diluted enzyme.

### 3.3. Assay Protocol for CDK3

1.Add 100 µl 10 mM ATP to 1.25 ml 6 µM substrate peptide. Dilute the mixture with dH20 to 2.5 ml to make 2X ATP/substrate cocktail ([ATP]=400 µM, [substrate]=3 µm).

2. Transfer enzyme from –80°C to ice. Allow enzyme to thaw on ice.

3. Microcentrifuge briefly at 4°C to bring liquid to the bottom of the vial. Return immediately to ice.

4. Add 1 ml 10X kinase buffer [1 ml 10X Kinase Buffer 250 mM Tris-HCl pH 7.5, 100 mM $MgCl_2$, 1 mM Na3VO4, 50 mM b-glycerophosphate, 20 mM dithiothreitol (DTT)] to 1.5 ml dH20 to make 2.5 ml 4X reaction buffer.

5. Dilute enzyme in 1.25 ml of 4X reaction buffer to make 4X reaction cocktail ([enzyme]=4.0 ng/µl in 4X reaction cocktail).

6. Add 12.5 µl of the 4X reaction cocktail to 12.5 µl/well of prediluted compound of interest (usually around 10 µM) and incubate for 5 minutes at room temperature.

7. Add 25 µl of 2X ATP/substrate cocktail to 25 µl/well preincubated reaction cocktail/compound.

Final Assay Conditions for a 50 µl Reaction

25 mM Tris-HCl (pH7.5),10 mM MgCl2, 5 mM b-glycerophosphate, 0.1 mM Na3VO4, 200 µM ATP, 2 mM DTT, 1.5 µM peptide, 50 ng CDK3/CycE Kinase

8. Incubate reaction plate at room temperature for 30 minutes.

9. Add 50 µl/well Stop Buffer (50 mM EDTA, pH 8) to stop the reaction.

10. Transfer 25 µl of each reaction to a 96-well streptavidin-coated plate containing 75 µl dH2O/well and incubate at room temperature for 60 minutes.

11. Wash three times with 200 µl/well PBS/T.

12. Dilute primary antibody, Phospho-Rb (Ser807/811) Antibody #9308, 1:1000 in PBS/T with 1% BSA. Add 100 µl/well primary antibody.

13. Incubate at 37°C for 120 minutes.

14. Wash three times with 200 µl/well PBS/T.

15. Dilute Europium labeled anti-rabbit antibody 1:1000 in PBS/T with 1% BSA. Add 100 µl/well diluted antibody.

16. Incubate at room temperature for 30 minutes.

17. *Wash five times with 200 µl/well PBS/T.

18. Add 100 µl/well DELFIA® Enhancement Solution.

19. Incubate at room temperature for 5 minutes.

20. Detect 615 nm fluorescence emission with appropriate Time-Resolved Plate Reader.

### 3.4. Assay Protocol for CDK4

All samples and controls should be tested in duplicate.

1) Thaw 5x Kinase assay buffer 1, ATP and 10x CDK4 substrate peptide. (Optional: If desired, add DTT to 5x Kinase assay buffer 1 to make a 10 mM concentration; e.g. add 10 µl of 1 M DTT to 1 ml 5x Kinase assay buffer 1)

2) Prepare the master mixture (25 µl per well): N wells x (6 µl 5x Kinase assay buffer 1 + 1 µl ATP (500 µM) + 5 µl 10x CDK4 substrate peptide + 13 µl distilled water). Add 25 µl to every well.

3) Add 5 µl of Inhibitor solution of each well labeled as "Test Inhibitor". For the "Positive Control" and "Blank", add 5 µl of the same solution without inhibitor (Inhibitor buffer).

4) Prepare 3 ml of 1x Kinase assay buffer 1 by mixing 600 µl of 5x Kinase assay buffer 1 with 2400 µl water. 3 ml of 1x Kinase assay buffer 1 is sufficient for 100 reactions.

5) To the wells designated as "Blank", add 20 µl of 1x Kinase assay buffer 1.

6) Thaw CDK4/CyclinD3 enzyme on ice. Upon first thaw, briefly spin tube containing enzyme to recover full content of the tube. Calculate the amount of CDK4/CyclinD3 required for the assay and dilute enzyme to ~10 ng/µl with 1x Kinase assay buffer 1. Store remaining undiluted enzyme in aliquots at -80°C. Note: CDK4/CyclinD3 enzyme is sensitive to freeze/thaw cycles. Avoid multiple freeze/thaw cycles. Do not re-use thawed aliquots or diluted enzyme.

Positive

7) Initiate reaction by adding 20 µl of diluted CDK4/CyclinD3 enzyme to the wells designated "Positive Control" and "Test Inhibitor Control". Incubate at 30°C for 60 minutes.

8) Thaw Kinase-Glo® Max Luminescence Kinase Assay reagent.

9) After the 60 minutes reaction, add 50 µl of Kinase-Glo® Max reagent to each well. Cover plate with aluminum foil and incubate the plate at room temperature for 10 ~ 15 minutes.

10) Measure luminescence using the microplate reader. "Blank" value is subtracted from all readings.

### 3.5. Assay Protocol for CDK5

1) Thaw 5x Kinase assay buffer 1, ATP and 10x CDK substrate peptide 1. (Optional: If desired, add DTT to 5x Kinase assay buffer 1 to make a 10 mM concentration; e.g. add 10 µl of 1 M DTT to 1 ml 5x Kinase assay buffer 1)

2) Prepare the master mixture (25 µl per well): N wells x (6 µl 5x Kinase assay buffer 1 + 1 µl ATP (500 µM) + 5 µl 10x CDK substrate peptide 1 + 13 µl distilled water). Add 25 µl to every well.

3) Add 5 µl of Inhibitor solution of each well labeled as "Test Inhibitor". For the "Positive Control" and "Blank", add 5 µl of the same solution without inhibitor (Inhibitor buffer).

4) Prepare 3 ml of 1x Kinase assay buffer 1 by mixing 600 µl of 5x Kinase assay buffer 1 with 2400 µl water. 3 ml of 1x Kinase assay buffer 1 is sufficient for 100 reactions.

5) To the wells designated as "Blank", add 20 µl of 1x Kinase assay buffer 1.

6) Thaw CDK5/p25 enzyme on ice. Upon first thaw, briefly spin tube containing enzyme to recover full content of the tube. Calculate the amount of CDK5/p25 required for the assay and dilute enzyme to ~0.75 ng/µl with 1x Kinase assay buffer 1. Store remaining undiluted enzyme in aliquots at -80°C. Note: CDK5/p25 enzyme is sensitive to freeze/thaw cycles. Avoid multiple freeze/thaw cycles. Do not re-use thawed aliquots or diluted enzyme.

7) Initiate reaction by adding 20 µl of diluted CDK5/p25 enzyme to the wells designated "Positive Control" and "Test Inhibitor Control". Incubate at 30°C for 45 minutes.

8) Thaw Kinase-Glo Max reagent.

9) After the 45-minute reaction, add 50 µl of Kinase-Glo Max reagent to each well. Cover plate with aluminum foil and incubate the plate at room temperature for 15 minutes.

10) Measure luminescence using the microplate reader. "Blank" value is subtracted from all readings.

### 3.6. Assay Protocol for CDK6

Dilute enzyme, substrate, ATP and inhibitors in Kinase Buffer.

• Add to the wells of 384 low volume plate:

o 1 µl of inhibitor or (5% DMSO)

o 2 µl of enzyme (defined from table 1)

o 2 µl of substrate/ATP mix

• Incubate at room temperature for 60 minutes.

• Add 5 µl of ADP-Glo™ Reagent

• Incubate at room temperature for 40 minutes.

• Add 10 µl of Kinase Detection Reagent

• Incubate at room temperature for 30 minutes.

• Record luminescence (Integration time 0.5-1second).

### 3.7. Assay Protocol for CDK7

1) Thaw 5x Kinase assay buffer 1, ATP and 10x CDK substrate peptide 2.

(Optional: If desired, add DTT to 5x Kinase assay buffer 1 to make a 10 mM concentration; e.g. add 10 µl of 1 M DTT to 1 ml 5x Kinase assay buffer 1)

2) Prepare the master mixture (12.5 µl per well): N wells x (3 µl 5x Kinase assay buffer 1 + 0.5 µl ATP (500 µM) + 1.25 µl CDK substrate peptide 2 (1 mg/ml) + 7.75 µl distilled water). Add 12.5 µl to every well.

3) Add 2.5 µl of Inhibitor solution of each well labeled as "Test Inhibitor". For the "Positive Control" and "Blank", add 2.5 µl of 10% DMSO in water (Inhibitor buffer).

4) Prepare 3 ml of 1x Kinase assay buffer 1 by mixing 600 µl of 5x Kinase assay buffer 1 with 2400 µl water. 3 ml of 1x Kinase assay buffer 1 is sufficient for 100 reactions.

5) To the wells designated as "Blank", add 10 µl of 1x Kinase assay buffer 1.

6) Thaw CDK7/Cyclin H/MAT1 enzyme on ice. Upon first thaw, briefly spin tube containing enzyme to recover full content of the tube. Calculate the amount of CDK7/Cyclin H/MAT1 required for the assay and dilute enzyme to ~10 ng/µl with 1x Kinase assay buffer 1. Store remaining undiluted enzyme in aliquots at -80°C. Note: CDK7/Cyclin H/MAT1 enzyme is sensitive to freeze/thaw cycles.

Avoid multiple freeze/thaw cycles. Do not re-use thawed aliquots or diluted enzyme.

7) Initiate reaction by adding 10 µl of diluted CDK7/Cyclin H/MAT1 enzyme to the wells designated "Positive Control" and "Test Inhibitor Control". Incubate at 30°C for 60 minutes.

8) Thaw ADP-Glo reagent.

9) After the 60 minute reaction, add 25 µl of ADP-Glo reagent to each well. Cover plate with aluminum foil and incubate the plate at room temperature for 45 minutes.

10) Thaw Kinase-Detection reagent

11) After the 45 minutes incubation, add 50 µl of Kinase Detection reagent to each well. Cover plate with aluminum foil and incubate the plate at room temperature for another 45 minutes.

12) Measure luminescence using the microplate reader. "Blank" value is subtracted from all readings.

### 3.8. Assay Protocol for CDK9

1)5x Kinase assay buffer 1, ATP and 5x CDK substrate peptide 2. (Optional: If desired, add DTT to 5x Kinase assay buffer 1 to make a 10 mM concentration; e.g. add 10 µl of 1 M DTT to 1 ml 5x Kinase assay buffer

2) Prepare the master mixture (25 µl per well): N wells x (6 µl 5x Kinase assay buffer 1 + 1 µl ATP (500 µM) + 10 µl 5x CDK substrate peptide 2 + 8 µl distilled water). Add 25 µl to every well.

3) Add 5 µl of Inhibitor solution of each well labeled as "Test Inhibitor". For the "Positive Control" and "Blank", add 5 µl of the same solution without inhibitor (Inhibitor buffer).

4) Prepare 3 ml of 1x Kinase assay buffer 1 by mixing 600 µl of 5x Kinase assay buffer 1 with 2400 µl water. 3 ml of 1x Kinase assay buffer 1 is sufficient for 100 reactions.

5) To the wells designated as "Blank", add 20 µl of 1x Kinase assay buffer 1.

6) Thaw CDK9/CyclinT enzyme on ice. Upon first thaw, briefly spin tube containing enzyme to recover full content of the tube. Calculate the amount of CDK9/CyclinT required for the assay and dilute enzyme to ~5 ng/µl with 1x Kinase assay buffer 1. Store remaining undiluted enzyme in aliquots at -80°C. Note: CDK9/CyclinT enzyme is sensitive to freeze/thaw cycles. Avoid multiple freeze/thaw cycles. Do not re-use thawed aliquots or diluted enzyme.

7) Initiate reaction by adding 20 µl of diluted CDK9/CyclinT enzyme to the wells designated "Positive Control" and "Test Inhibitor Control". Incubate at 30°C for 45 minutes.

8) Thaw Kinase-Glo Max reagent.

9) After the 45-minute reaction, add 50 µl of Kinase-Glo Max reagent to each well. Cover plate with aluminum foil and incubate the plate at room temperature for 15 minutes.

10) Measure luminescence using the microplate reader. "Blank" value is subtracted from all readings.

### 3.9. Inhibition of Phospho-CDK1 / CDC2 Cell-Based Phosphorylation in SK-MEL-5 cancer cell

The assay was performed according to the following protocol

1) Seed 200 µl of 20,000 adherent cells in culture medium in each well of a 96-well plate. The plates included in the kit are sterile and treated for cell culture. For suspension cells and loosely attached

cells, coat the plates with 100 µl of 10 µg/ml Poly-L-Lysine (not included) to each well of a 96-well plate for 30 minutes at 37°C prior to adding cells.

2) Incubate the cells for overnight at 37°C, 5% CO2.

3) Treat the cells as desired.

4) Remove the cell culture medium and rinse with 200 µl of 1x TBS, twice.

5) Fix the cells by incubating with 100 µl of Fixing Solution for 20 minutes at room temperature. The 4% formaldehyde is used for adherent cells and 8% formaldehyde is used for suspension cells and loosely attached cells. During the incubation, the plates should be sealed with Parafilm. Note: Fixing Solution is volatile.Wear appropriate personal protection equipment (mask, gloves and glasses) when using this chemical.

6) Remove the Fixing Solution and wash the plate 3 times with 200 µl 1x Wash Buffer for five minutes each time with gentle shaking on the orbital shaker. The plate can be stored at 4°C for a week.

Note: For all wash steps, tap the plate gently on absorbent papers to remove the solution completely.

7) Add 100 µl Quenching Buffer and incubate for 20 minutes at room temperature.

8) Wash the plate 3 times with 1x Wash Buffer for 5 minutes at a time, with gentle shaking on the shaker.

9) Add 200 µl of Blocking Buffer and incubate for 1 hour at room temperature.

10) Wash 3 times with 200 µl of 1x Wash Buffer for 5 minutes at a time, with gentle shaking on the shaker.

11) Add 50 µl of 1x primary antibodies (Anti-CDC2 (Phospho-Tyr15) Antibody, Anti-CDC2 Antibody and/or Anti-GAPDH Antibody) to the corresponding wells, cover with Parafilm and incubate for 16 hours (overnight) at 4°C. If the target expression is known to be high, incubate for 2 hours at room temperature with gentle shaking on the shaker.

12) Wash 3 times with 200 µl of 1x Wash Buffer for 5 minutes at a time, with gentle shaking on the shaker.

13) Add 50 µl of 1x secondary antibodies (HRP-Conjugated Anti- Rabbit IgG Antibody and/or HRP-Conjugated Anti-Mouse IgG Antibody) to corresponding wells and incubate for 1.5 hours at room temperature with gentle shaking on the shaker. Note: Add HRP-Conjugated Anti-Rabbit IgG Antibody to the wells incubated with Anti-CDC2 (Phospho-Tyr15) Antibody (rabbit, polyclonal) and/or Anti-CDC2 (rabbit, polyclonal) and add HRP-Conjugated Anti-Mouse IgG Antibody to the wells incubated with Anti-GAPDH Antibody (mouse, monoclonal).

14) Wash 3 times with 200 µl of 1x Wash Buffer for 5 minutes at a time, with gentle shaking on the shaker.

15) Add 50 µl of Ready-to-Use Substrate to each well and incubate for 30 minutes at room temperature in the dark with gentle shaking on the shaker. Note: Ready-to-Use Substrate is a light-sensitive

reagent. Keep away from light.

16) Add 50 µl of Stop Solution to each well and read OD at 450 nm immediately using the microplate reader.

### 3.10. Caspase-3 activation assay

Caspase assay is performed according to the following procedures:

1. Allowing all reagents to reach room temperature before use. Gently mix all liquid reagents prior to use (Note: A standard curve must be run with each assay).
2. Determine the number of 8-well strips needed for the assay. Insert these in the frame(s) for current use. (Re-bag extra strips and frame. Store these in the refrigerator for future use).
3. Add 100 µL of the Standard Diluent Buffer to the zero standard wells. Well(s) reserved for chromogen blank should be left empty.
4. Add 100 µL of standards and controls or diluted samples to the appropriate microtiter wells. The sample dilution chosen should be optimized for each experimental system. Tap gently on side of plate to mix.
5. Cover wells with plate cover and incubate for 2 hours at room temperature.
6. Thoroughly aspirate or decant solution from wells and discard the liquid. Wash wells 4 times. See Directions for Washing.
7. Pipette 100 µL of Caspase-3 (Active) Detection Antibody solution into each well except the chromogen blank(s). Tap gently on the side of the plate to mix.
8. Cover plate with plate cover and incubate for 1 h at room temperature.
9. Thoroughly aspirate or decant solution from wells and discard the liquid.
10. Wash wells 4 times. See Directions for Washing.

11. Add 100 µL Anti-Rabbit IgG HRP Working Solution to each well except the chromogen blank(s). Prepare the working dilution as described in Preparing IgG HRP.
12. Cover wells with the plate cover and incubate for 30 minutes at room temperature.
13. Thoroughly aspirate or decant solution from wells and discard the liquid. Wash wells 4 times. See Directions for Washing.
14. Add 100 µL of Stabilized Chromogen to each well. The liquid in the wells will begin to turn blue.
15. Incubate for 30 min at room temperature and in the dark. Note: Do not cover the plate with aluminum foil or metalized mylar. The incubation time for chromogen substrate is often determined by the microtiter plate reader used. Many plate readers have the capacity to record a maximum optical density (O.D.) of 2.0. The O.D. values should be monitored and the substrate reaction stopped before the O.D. of the positive wells exceeds the limits of the instrument. The O.D. values at 450 nm can only be read after the Stop Solution has been added to each well. If using a reader that records only to 2.0 O.D., stopping the assay after 20 to 25 minutes is suggested.
16. Add 100 µL of Stop Solution to each well. Tap side of plate gently to mix. The solution in the wells should change from blue to yellow.
17. Read the absorbance of each well at 450 nm having blanked the plate reader against a chromogen blank composed of 100 µL each of Stabilized Chromogen and Stop Solution. Read the plate within 2 hours after adding the Stop Solution.
18. Use a curve fitting software to generate the standard curve. A four-parameter algorithm provides the best standard curve fit.

19. Read the concentrations for unknown samples and controls from the standard curve. Multiply value(s) obtained for sample(s) by the appropriate dilution factor to correct for the dilution in step 3. Samples producing signals greater than that of the highest standard should be diluted in Standard Diluent Buffer and reanalyzed.

### 3.11. Protocol of Docking Studies

The automated docking simulation study is performed using Molecular Operating Environment (MOE®) version 2014.09, at Assiut University Faculty of Pharmacy, Chemical Computing Group Inc., and Montreal, Canada. The X-ray crystallographic structure of the target kinase (PDB: ID 4YC3) was obtained from Protein data bank. The target compounds were constructed into a 3D model using the builder interface of the MOE program. After checking their structures and the formal charges on atoms by 2D depiction, the following steps were carried out:

• The target compounds were subjected to a conformational search.

• All conformers were subjected to energy minimization, all the minimizations were performed with MOE until an RMSD gradient of 0.01 Kcal/mole and an RMS distance of 0.1 Å with MMFF94X force-field and the partial charges were automatically calculated.

The enzyme was prepared for docking studies by:

• Hydrogen atoms were added to the system with their standard geometry.

• The atoms connection and type were checked for any errors with automatic correction.

• Selection of the receptor and its atoms potential were fixed.

• The MOE® Alpha Site Finder was used for the active site search in the enzyme structure using all default items. Dummy atoms were created from the obtained alpha Spheres.
